# DNA Noncovalent Interactions of Dinuclear η^6^‐Arene Ru(II) Complexes: Influence of Complex Charge and Bridging Ligand Length on DNA Binding Mode and Cytotoxic Activity

**DOI:** 10.1002/chem.202502680

**Published:** 2025-11-10

**Authors:** Dimitrios Thomos, Theodoros Tsolis, John C. Plakatouras, Ioannis‐Michail Chronakis, Angeliki Magklara, Achilleas Garoufis

**Affiliations:** ^1^ Department of Chemistry, Laboratory of Inorganic Chemistry University of Ioannina Ioannina GR‐45110 Greece; ^2^ Department of Clinical Chemistry, Faculty of Medicine, School of Health Sciences Ioannina, Greece, Institution University of Ioannina Ioannina GR‐45110 Greece; ^3^ Biomedical Research Institute‐Foundation for Research and Technology Ioannina GR‐45110 Greece; ^4^ Institute of Biosciences University Research Center of Ioannina (URCI) Ioannina GR‐45110 Greece

**Keywords:** crystal structures, cytotoxicity, dinuclear ru(ii)‐η(6) arene complexes, intercalation, noncovalent DNA interactions

## Abstract

A series of dinuclear η⁶‐arene Ru(II) complexes with the general formulae {[(η⁶‐p‐cym)Ru(L1)]_2_(μ‐BL‐i)}^2+^ (**1**)–(**3**) and {[(η⁶‐p‐cym)_2_Ru_2_(L1)(L2)](μ‐BL‐i)}^3+^ (**4**)–(**6**), [L1 = benzo[h]quinoline (**bq**), L2 = 1,10‐phenanthroline (**phe**) and BL‐i the bridging ligands with i = 1 ‐3, 4,4′‐bipyridine (BL‐1), 1,2‐bis(4‐pyridyl)ethane (BL‐2) and 1,3‐bis(4‐pyridyl)propane (BL‐3)] were synthesized and fully characterized by high‐resolution mass spectrometry and NMR spectroscopy. The crystal structures of the complexes (**1**)–(**3**) were determined by single‐crystal X‐ray diffraction. DNA binding interactions of the water‐soluble chloride salts of the complexes (**4**)–(**6**) were investigated by NMR spectroscopy using the B‐type DNA model oligonucleotide duplex d(5′‐CGCGAATTCGCG‐3′)_2_. ^1^H NMR chemical shift changes revealed that the chelating ligands (**phe** or **bq**) partially intercalate and/or bind in the helix minor groove. Among these, complex {[(η⁶‐p‐cym)_2_Ru_2_(phe)(bq)](μ‐BL‐1)}^3+^, (**4**), induced the most pronounced perturbations, including partial helix unwinding. Fluorescence quenching experiments yielded the Stern–Volmer constants (*K*_sv_) and the binding constants (*K*_b_) in the moderate‐to‐high affinity range, consistent with the NMR observations. Preliminary cytotoxicity studies showed that the chloride salts of the complexes (**1**)–(**6**) exhibit low‐micromolar IC_50_ values against MCF‐7, A2780, and cisplatin‐resistant A2780cis‐resistant cell lines, while showing moderate selectivity for cancer cells over normal fibroblasts.

## Graphical abstract

A library of cationic dinuclear Ru(II)‐η⁶‐arene analogues was developed to assess how overall charge and Ru–Ru distance influence DNA binding noncovalent interactions and cytotoxicity. NMR studies showed multiple binding modes with the B‐form DNA oligonucleotide d(5΄‐CGCGAATTCGCG‐3΄)_2_, while the complexes displayed micromolar IC_50_ values against MCF‐7, A2780, and A2780cis‐resistant cell lines.

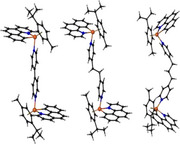

## 1 Introduction

In cancer chemotherapy, three globally approved Pt(II)‐based drugs, cisplatin^[^ ^1^, ^2^ ^]^ oxaliplatin^[^ ^3^ ^]^ and carboplatin,^[^ ^4^ ^]^ have been extensively employed in clinical practice. However, their therapeutic efficacy is frequently limited by dose‐dependent toxicities and low responsiveness in certain tumor types, underscoring the need for the development of novel chemotherapeutic agents. Among the most promising alternatives are metal‐based compounds,^[^ ^5^ ^]^ which are being designed to provide enhanced efficacy and reduced toxicity compared to Pt(II)‐based drugs.

Ruthenium complexes generally exhibit lower toxicity toward healthy cells lines compared to platinum complexes,^[^ ^6^ ^]^ while maintaining potent cytotoxic activity against cancer cells. This favorable therapeutic profile has positioned them as promising chemotherapeutics and has attracted considerable research interest over the past two decades^[^ ^7^ ^]^. Among these, organometallic Ru(II) complexes featuring η⁶‐coordinated arenes, adopting a piano‐stool geometry, have garnered considerable attention^[^ ^8^, ^9^ ^]^. Notable examples include the extensively studied cytotoxic Ru(II)‐η⁶‐arene with chelated ethylenediamine complex (RAED), developed by Sadler,^[^ ^10^, ^11^ ^]^ and the antimetastasis complex triaza‐phosphaadamantane (PTA) Ru(II) η^6^‐arene (RAPTA), introduced by Dyson^[^ ^12^, ^13^ ^]^. The RAED complex interacts with the DNA of chromatin, while the RAPTA preferentially with the histone proteins^[^ ^14^ ^]^; in certain cases, the former mimicking the mechanism of Pt(II)‐based‐drugs, whereas the latter, through interactions with alternative cellular components, may give rise to distinct therapeutic activities.^[^ ^15^, ^16^ ^]^. Thus, beyond their cytotoxic effects, many of these compounds display a broad spectrum of biological activities, including antimetastatic^[^ ^17^, ^18^ ^]^ and antiangiogenic properties,^[^ ^19^, ^20^, ^21^ ^]^ which are highly dependent on the nature of the coordinated ligands, a key factor that continues to drive innovation and research in the field. In addition to the synthesis and screening of hundreds of mononuclear complexes analogous to RAPTA and RAED, considerable efforts have been directed toward the development of polynuclear derivatives^[^ ^18^, ^22^, ^23^, ^24^, ^25^, ^26^, ^27^, ^28^, ^29^, ^30^ ^]^ with dinuclear complexes attracting particular interest^[^ ^31^ ^]^.

Typically, dinuclear systems consist of two Ru–η⁶‐arene units linked either by a bidentate bridging ligand (BL) or by a multidentate ligand. A key structural feature that distinguishes these complexes is the number of kinetically labile ligands on the ruthenium center, which governs the availability of reactive sites for interactions with biomolecular targets. Based on this characteristic, dinuclear Ru–arene complexes can be classified into three categories: bifunctional complexes (RAPTA‐type dinuclear),^[^ ^31^, ^32^, ^33^, ^34^, ^35^, ^36^, ^37^ ^]^ monofunctional complexes (RAED‐type dinuclear),^[^ ^38^, ^39^, ^40^, ^41^, ^42^, ^43^, ^44^, ^45^, ^46^, ^47^, ^48^, ^49^, ^50^, ^51^, ^52^, ^53^, ^54^, ^55^, ^56^, ^57^, ^58^, ^59^, ^60^ ^]^ and fully substituted with kinetically inert ligands. The majority of the latter are based on thiosemicarbazone derivatives,^[^ ^50^, ^61^, ^62^, ^63^, ^64^, ^65^ ^]^ thiolato‐bridged complexes,^[^ ^66^, ^67^, ^68^, ^69^, ^70^, ^71^, ^72^ ^]^ while complexes featuring polypyridyl ligands are less commonly reported^[^ ^73^, ^74^ ^]^. The cytotoxic mechanism of these complexes remains poorly understood and may involve several pathways. These include mitochondrial accumulation,^[^ ^66^, ^68^, ^70^, ^71^ ^]^ catalytic oxidation of glutathione, to glutathione disulfide,^[^ ^71^ ^]^ binding to proteins,^[^ ^64^, ^66^ ^]^ and noncovalent interactions with DNA^[^ ^61^, ^62^, ^63^, ^73^ ^]^ which result in structural alterations of the DNA and lead to cell death^[^ ^75^ ^]^. However, a major advantage of dinuclear complexes, over their mononuclear counterparts, is the presence of two Ru(II)‐η⁶‐arene units, which can simultaneously bind to spatially distant sites on DNA, defined by the linker length, thereby inducing long‐range structural distortions that impede the DNA repair mechanisms^[^ ^76^, ^77^ ^]^. Moreover, the lipophilic and positively charged polynuclear complexes are capable of traversing the cell membrane in response to the membrane potential^[^ ^78^, ^79^ ^]^. In many cancer cell types, the membrane exhibits a negatively charged surface potential,^[^ ^80^ ^]^ thereby promoting the cellular uptake of these cationic species. Furthermore, the positively charged complexes exhibit a strong affinity toward the negatively charged DNA phosphate backbone^[^ ^81^, ^82^, ^83^ ^]^.

Building on these insights, we have designed dinuclear η⁶‐arene‐Ru(II) complexes bearing polypyridyl ligands, capable of engaging in noncovalent interactions with two spatially separated sites on the DNA through the polypyridine ligands, with their binding distance governed by the linker length. In general, the study of noncovalent interactions between metal complexes and DNA represents an important topic of inorganic medicinal chemistry, covering aspects such as DNA binding modes, sequence selectivity, DNA‐binding affinity, and cytotoxicity^[^ ^84^, ^85^, ^86^ ^]^.

In our previous study,^[^ ^73^ ^]^ we explored the influence of the BL length, on the cytotoxic activity and DNA‐binding properties of a series of tetracationic dinuclear η⁶‐arene‐Ru(II) complexes with the general formula {[(η⁶‐p‐cym)Ru(L)]_2_(μ‐BL)}^4+^, where L = bpy or phe, and BL = 4,4′‐bipyridine, 1,2‐bis(4‐pyridyl)ethane and 1,3‐bis(4‐pyridyl)propane. The complexes exhibited distinct DNA‐binding affinities and binding modes toward the B‐form DNA‐model oligonucleotide d(5′‐CGCGAATTCGCG‐3′)_2_. Furthermore, they demonstrated good cytotoxic activity against both the A2780 ovarian cancer cell line and its cisplatin‐resistant variant (A2780cis‐res), showing selectivity indices (SI) in the range of 3.0 to 5.9. In our next study,^[^ ^87^ ^]^ we attempted to better understand the mechanism of interaction of the same complexes with DNA, and more specifically, we tried to determine if there is any sequence specificity regarding the complex‐DNA interactions.

**Scheme 1:**
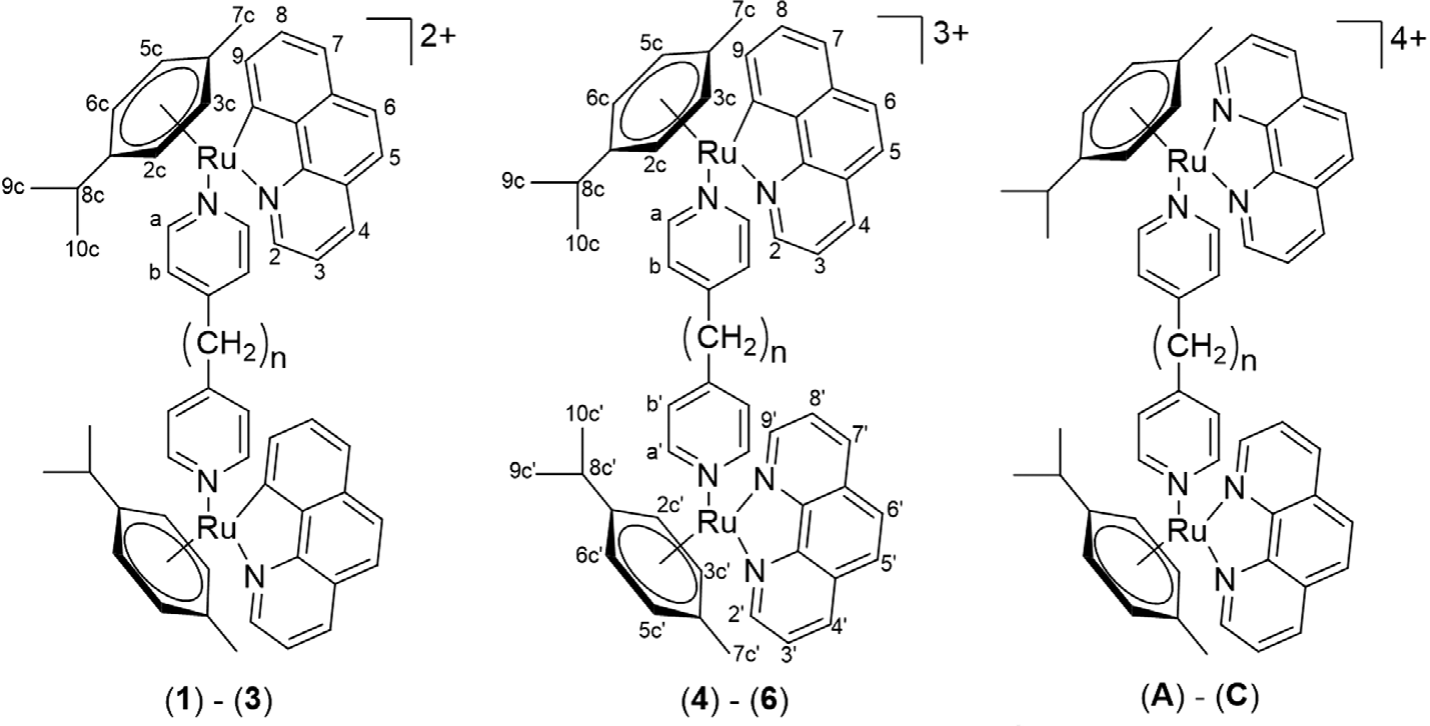
Structures and numbering of the studied complexes, (**1**)–(**6**), *n* = 0, 2, 3. Complexes (**A**)–(**C**) are derived from our previous studies^[^ ^73^, ^87^ ^]^.

In the present study, to investigate the influence of the overall charge on the DNA‐binding properties and cytotoxicity of analogous dinuclear η⁶‐arene‐Ru(II) complexes (scheme 1), we replaced the neutral ligand **phe** with its monoanionic analogue **bq**, thereby synthesizing the complexes (1)–(3) with an overall charge of + 2. In addition, we synthesized the mixed‐ligand **bq‐phe** complexes (4)–(**6**) bearing a total charge of + 3. For consistency and comparative analysis, we employed the same DNA sequence, BLs, and cancer cell lines used in our previous study,^[^ ^73^ ^]^ enabling a systematic comparison of structurally related cationic complexes across a series with overall charges of + 4, +3, and + 2.

## 2 Results and Discussion

### 2.1 Synthesis of the Complexes (1)–(**6**)

**Scheme 2:**
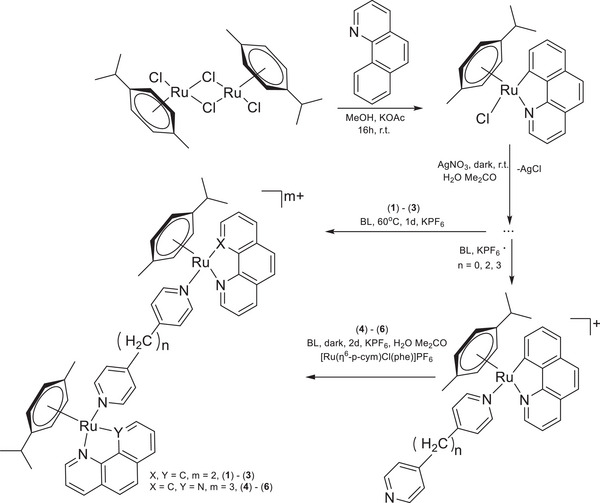
Synthetic procedure for complexes (**1**)–(**6**).

The complexes (**1**)–(**6**) were synthesized according to the procedure depicted in Scheme 2. Specifically, the dimeric precursor [Ru(η⁶‐p‐cymene)Cl_2_]_2_ was cleaved by **bq** under alkaline conditions, as previously described^[^ ^88^ ^]^. The resulting complex [Ru(η⁶‐p‐cymene)Cl(bq)], was then treated in situ with AgNO_3_ to remove the chloride ligand and the precipitated AgCl was filtered off. Without isolating the intermediate, the appropriate BLs were added. Complexes (**1**)–(**3**) were obtained by precipitation as [PF_6_]^−^ salts upon addition of KPF_6_ to the reaction mixture. For the mixed‐ligand complexes (**4**)–(**6**), the respective BLs were added following chloride abstraction, and the complexes of the formula [Ru(η⁶‐p‐cymene)(bq)(BL)]PF_6_ were isolated. Subsequent reaction with [Ru(η⁶‐p‐cymene)Cl(phe)]PF_6_, afforded the final bimetallic complexes, which were also isolated as [PF_6_]^−^ salts.

### 2.2 Solution Characterization of the Complexes (**1**)–(**6**)

In the ^1^H NMR spectra of complexes (1)–(3) one set of signals is observed, consistent with symmetrical molecular structures. The ligand **bq** coordinates in a bidentate N,C‐chelating fashion through the nitrogen atom N1 and the deprotonated carbon atom C1, acting as anionic ligand. The **bq**H2 and **bq**H9, located adjacent to the coordination sites, exhibit significant downfield shifts relative to the free ligand. The absence of a signal corresponding to the C11 proton further supports deprotonation and coordination through this carbon atom. Regarding the BLs, a single set of signals is also observed, indicating both their symmetrical nature and their bridging coordination, while the p‐cymene remains η^6^‐coordinated. Notably, four distinct signals are observed for the cymene aromatic protons, highlighting the loss of equivalence caused by restricted rotation or asymmetry in the ruthenium coordination sphere (Figures S7–S9). In contrast, the similar complexes (**A**)–(**C**), bearing the symmetrical ligand **phe** instead of **bq,** display only two signals (H2c/H6c and H3c/H5c), as reported previously^[^ ^73^ ^]^. Furthermore, in complexes (1)–(3), these signals appear significantly upfield, (‐0.4 to ‐1.0 ppm) compared to the corresponding (**A**)–(**C**), which is consistent with a close spatial proximity between the cymene and **bq** aromatic rings, likely arising from hydrophobic interactions.

To gain insight into the relative arrangement of the coordinated ligands, a series of 2D Nuclear Overhauser Effect Spectroscopy (NOESY) experiments was performed. The observed interligand NOE cross‐peaks between the p‐cymene, BLs, and **bq** are summarized in Tables S2–S3 and Figures S19–S24.

**Figure 1:**
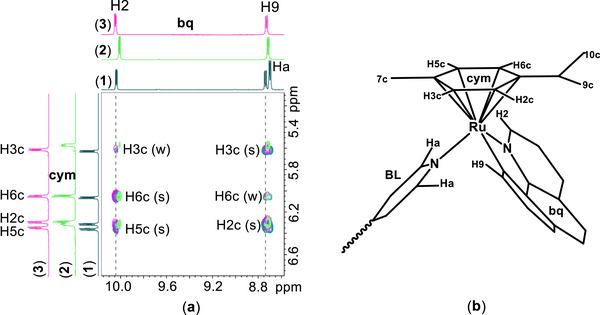
a) Selected part of the superimposed NOESY spectra of the complexes (1)–(3) recorded in acetone‐d_6_ with a *t*_mix_ = 600 ms, 298 K, showing proton assignments and qualitative assessment of the cross‐peak intensities: (s) = strong, (m) = medium, and (w) = weak. b) Partial molecular structure of complexes (1)–(3) highlighting the protons involved in the NOESY correlations shown in (a).

In the NOESY spectra of complexes (1)–(3), strong intensity cross‐peaks were observed between **bq**H9 and the p‐cymene protons H2c and H3c, as well as between **bq**H2 and p‐cymene H5c and H6c (Figure 1), indicating a defined spatial orientation of these ligands. These correlations suggest that the p‐cymene is oriented with its isopropyl group positioned above the ligand **bq**, while its methyl group lies on the opposite side. This arrangement may be stabilized by CH–π interactions between the isopropyl group and the electron‐rich aromatic ring system of **bq**. Such interactions may also explain the pronounced upfield shifts observed for the isopropyl protons, which are consistent with shielding effects induced by the ligand **bq**. Notably, these shielding effects differ between the pyridine‐like ring and the fused phenyl ring of **bq,** leading to the splitting of the H10c and H9c signals into two distinct doublets. Furthermore, the restricted rotation of the arene in this geometry likely contributes to the observed nonequivalence of the cymene aromatic protons. In this conformation, H7c of cymene is positioned in proximity to the pyridine ring of the BLs, which is supported by the presence of strong NOE cross‐peaks between H7c and the BL protons Ha and Hb.

The ^1^H NMR spectra of complexes (4)–(6) are more complex than (1)–(3), due to their reduced symmetry. While the protons of the **phe** ligand appeared equivalent, the **bq** protons exhibit distinct signals reflecting its nonsymmetric environment. Accordingly, the protons of the two p‐cymene moieties become chemically nonequivalent. Thus, at the ruthenium center coordinated to **phe**, the aromatic protons of cymene (**cym′**) give rise to two signals corresponding to H2c′H6c′ and H3c′H5c′, respectively. In contrast, at the ruthenium center coordinated to **bq**, the aromatic protons of **cym** appear as four distinct signals reflecting the asymmetric coordination environment. (Figures S10–S12)). Moreover, these signals appear significantly upfield compared to those of the **cym′**, suggesting a stronger CH‐π interaction between the **bq‐cym** than **phe‐cym′**. Alternatively, this difference could reflect variations in the strengths of the Ru─C and Ru─N bonds, which modulate the electron density at the η⁶‐coordinated cymene.

**Figure 2:**
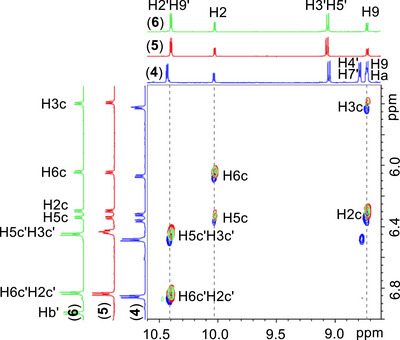
Selected part of the superimposed NOESY spectra of the complexes (4)–(6) recorded in acetone‐d_6_ with *t*_mix_ = 600 ms, 298 K, showing proton assignments and the corresponding cross‐peaks.

Regarding the spatial orientation of **cym** relative to the **bq**, the NOE patterns observed in complexes (**4**)–(**6**)are consistent with those of (1)–(3), indicating that the isopropyl group of **cym** is positioned above the **bq** aromatic ring (Figure 2).

**Figure 3:**
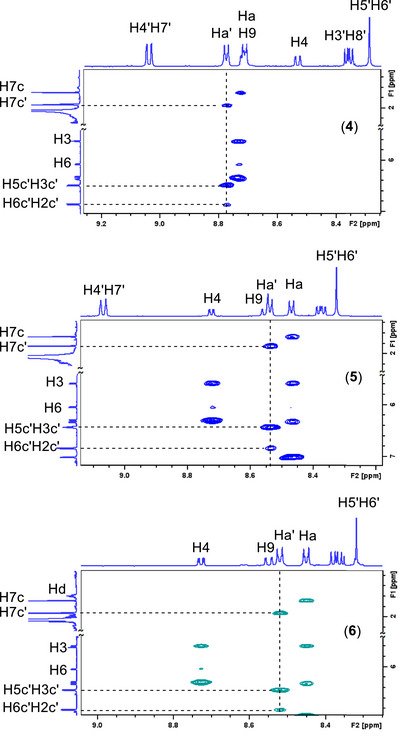
Selected part of the NOESY spectra of the complexes (4)–(6) recorded in acetone‐d6 with a *t*_mix_ = 600 ms, at 298 K, showing interligand cross‐peaks between the cymene aromatic protons H5c΄H3c΄ / H6c΄H2c΄ and the Ha΄ of BLs.

However, in the case of **cym′** the chemical equivalence of H2c′H6c′ and H3c′H5c′ prevents determination of its orientation based on the same NOE patterns used for **cym**. To address this, the relative intensities of the NOESY cross‐peaks between the **BL**Ha′ and the **cym′** protons were examined. The presence of weak cross‐peaks between **BL**Ha′ and H2c′H6c′, combined with a significantly stronger cross‐peak between H3c′H5c′ and H7c′, suggests that the methyl group of **cym′** is oriented toward the BL, while the isopropyl group is positioned toward the aromatic ring system of **phe** (Figure 3). Overall, this arrangement closely resembles the orientation observed for **cym** relative to **bq**, indicating a similar positioning across both metal centers for all the complexes (4)–(6).

### 2.3 Crystal Structures of the Complexes (**1**)–(**3**)

**Figure 4:**
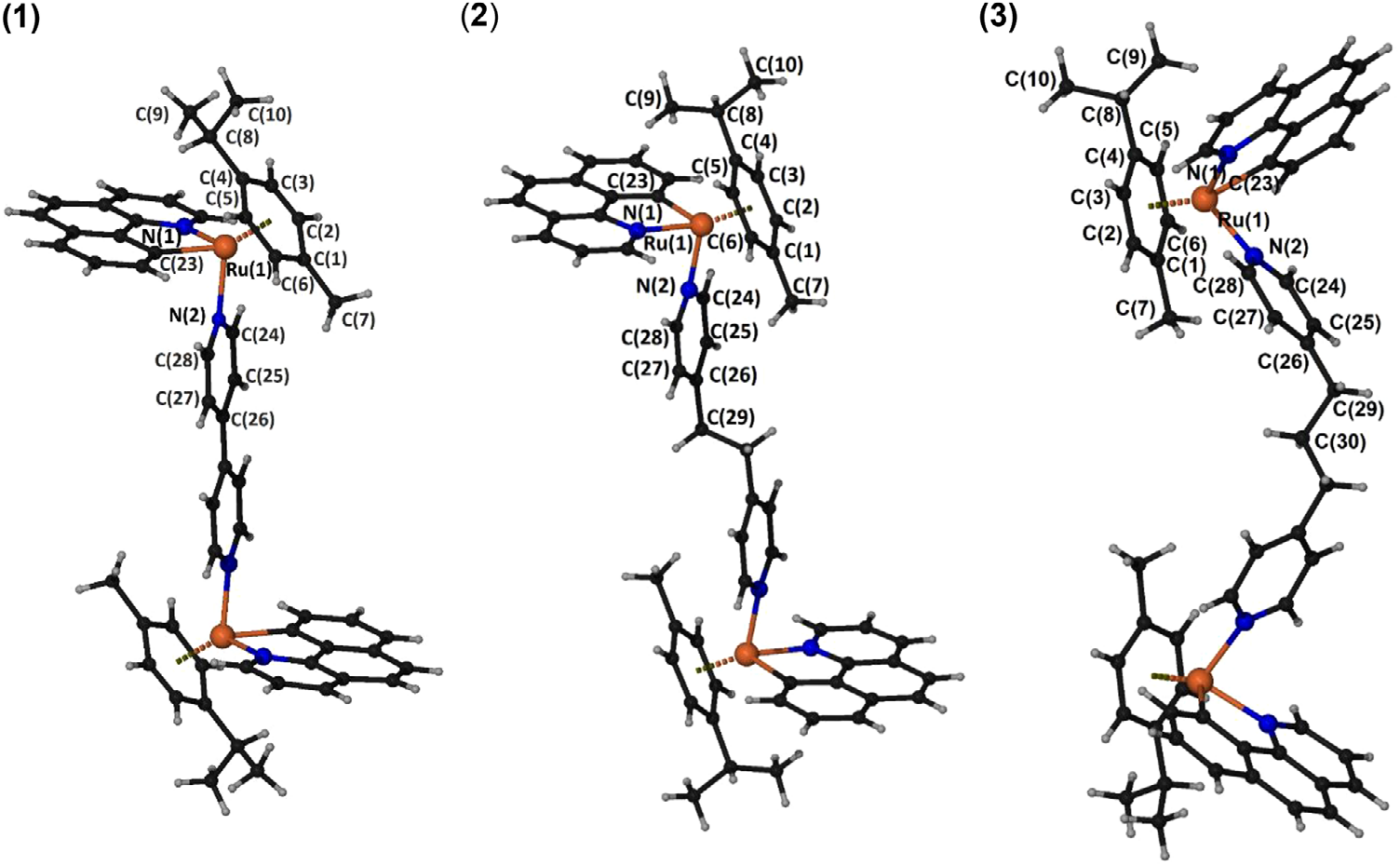
Ball‐and‐stick presentations of the cations of (**1**), (**2**), and (**3**). Symmetry operations to generate equivalent atoms: (**1**), ‐*x* + 2, ‐*y* + 1, ‐*z* + 2; (**2**), ‐*x* + 1, ‐*y*, ‐*z* + 1; (**3**), ‐*x*, *y*, ‐*z* + 1/2.

Suitable crystals for X‐ray single‐crystal analysis of the compounds were grown in a closed vessel through slow diffusion of diethyl ether vapors into acetonitrile/chloroform solutions of the complexes. Compounds (1) and (2) crystallize in the monoclinic space groups *P*2_1_/*c* and *P2_1_/n* while **3** is orthorhombic P*bcn*. Details on the structure's solution and refinement can be found in the supplementary material (Table S1). Here, we comment on the structures of the cations present in the unit cells; their structures are shown in Figure 4 while selected bond lengths and angles are presented in Table 1.

**Table 1:** Selected bond distances (Å) and angles (^o^) for compounds (**1**)–(**3**).

	(1)	(2)	(3)
Ru(1)–cym C_6_ centroid	1.722(1)	1.702(1)	1.696(1)
Ru(1)–C(23)	2.089(12)^[^ ^a^ ^]^	2.053(6)	2.083(6)
Ru(1)–N(1)	2.099(9)^[^ ^a^ ^]^	2.080(6)	2.088(6)
Ru(1)–N(2)	2.133(7)	2.103(5)	2.102(6)
Ru(1)–Ru(1)	11.361(2)	13.133(2)	12.522(1)
cym C_6_ centroid–Ru(1)–N(1)	131.24(4)^[^ ^a^ ^]^	130.63(2)	130.69(2)
cym C_6_ centroid–Ru(1)–C(23)	127.88(5)^[^ ^a^ ^]^	131.38(2)	129.97(2)
cym C_6_ centroid–Ru(1)–N(2)	128.15(2)	124.64(3)	127.34(2)
N(1)–Ru(1)–C(23)	77.1(5)^[^ ^a^ ^]^	79.0(2)	77.9(2)
N(1)–Ru(1)–N(2)	88.8(4)	88.0(2)	86.8(2)
N(2)–Ru(1)–C(23)	86.8(6)	87.5(2)	87.5(2)

^[a]^ The **bq** ligand is disordered and refined in two positions. The data from the part with the highest occupancy values are used.

In all the compounds the cations lay on symmetry elements; inversion centers in the middle of the BLs in (**1**) and (**2**), and a twofold axis (the bisect of the angle C(29)–C(30)–C(29)[‐*x*, *y*, ‐*z* + 1/2]) in (**3**). The metallic centers of the complexes adopt the expected piano stool (pseudo‐octahedral) geometry with minor differences from each other.

The ruthenium atoms are surrounded by six carbon atoms of the π‐bonded cymene, one σ‐bonded carbon and one nitrogen atom belonging to the chelated **bq** ligand and a nitrogen atom belonging to the BL.

To the best of our knowledge there are no structurally characterized Ru(II) complexes with a six‐membered aromatic ligand, bq and a second heterocycle in the coordination sphere. The closest examples are the mononuclear compounds [Ru(η^6^‐benzene)(bq)Cl],^[^ ^89^ ^]^ {Ru(η^6^‐p‐cymene)(bq)[p‐C_6_H_4_(CF_3_)]},^[^ ^90^ ^]^ and [Ru(η^6^‐p‐cymene)(bq)Cl]^[^ ^91^, ^92^ ^]^. The bond distances and angles around the ruthenium centers in compounds (1)–(3) are similar to the corresponding of those mononuclear complexes.

There are a couple of features worthy to be mentioned in the structures of the cations in (1)–(3). In all cations the cymene's propyl group is directed toward the **bq** ligand, while the methyl group toward the pyridine ring of the BL. While in complexes (1) and (2) the planes defined by the **bq** ligands are parallel to each other as induced by symmetry, and the conformation of the dinuclear cations can be considered “*trans*,” the twofold axis mentioned in the beginning of this discussion for (3), leads to “cis” configuration and an angle between the **bq** planes in the same cation being 82.1(1)°. This affects the Ru─Ru distances in the dinuclear cations, positioning the Ru(1)–Ru(1) distance in (3) between the corresponding distances in (1) and (2).

### 2.4 NMR Studies on the Interactions of Complexes (4)–(6) Chloride Salts With the DNA Duplex D(5′‐CGCGAATTCGCG‐3′)_2_

**Figure 5:**
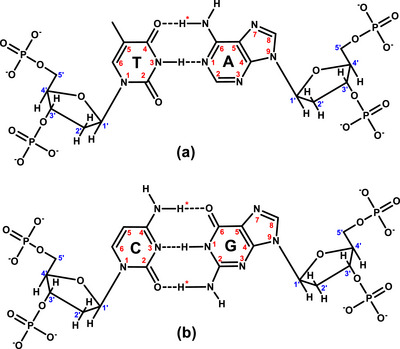
Structures and numbering for a) A·T and b) C·G base pairs.

To investigate the DNA binding mode of the synthesized complexes, NMR titration experiments were performed using the B‐type DNA model duplex d(5′‐CGCGAATTCGCG‐3′)_2_ (Figure 5) at increasing molar ratios. The spectra were recorded in H_2_O:D_2_O (9:1) with a 100 mM phosphate buffer (pH 7.0) at 298 K. Due to the limited water solubility of complexes (1)–(3), NMR studies could only be conducted for the + 3 charged complexes (4)–(**6**), which were converted to their chloride salts, designated as (**4a**)–(**6a**).^[^ ^93^, ^94^ ^]^

To elucidate the mode of interaction between complexes (**4a**)–(**6a**) and the oligonucleotide, three main aspects were investigated: (a) changes in the proton signals of the complexes compared to their free form upon addition to d(5′‐CGCGAATTCGCG‐3′)_2_, (b) changes in the signals of the nonexchangeable protons of d(5′‐CGCGAATTCGCG‐3′)_2_, and (c) changes in its exchangeable protons, with particular emphasis on the imino and amino protons of the nucleobases (Figures S25–S59 and Tables S4–S12).

**Figure 6:**
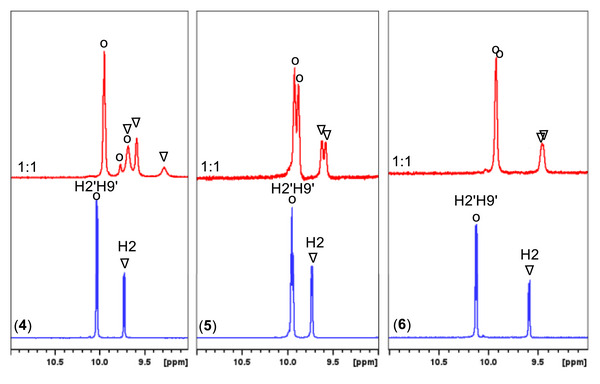
Part of ^1^H NMR spectra (9.0–11.0 ppm) of complexes (4a)–(6a) (blue) and their corresponding mixtures with the d(5′‐CGCGAATTCGCG‐3′)2 at a 1:1 ratio (red), under the same conditions (H2O:D2O, 9:1, phosphate buffer 100 mM, pH = 7.0, 298 K). Circles denote pheH2’H9’ and triangles bqH2 signals.

**Figure 7:**
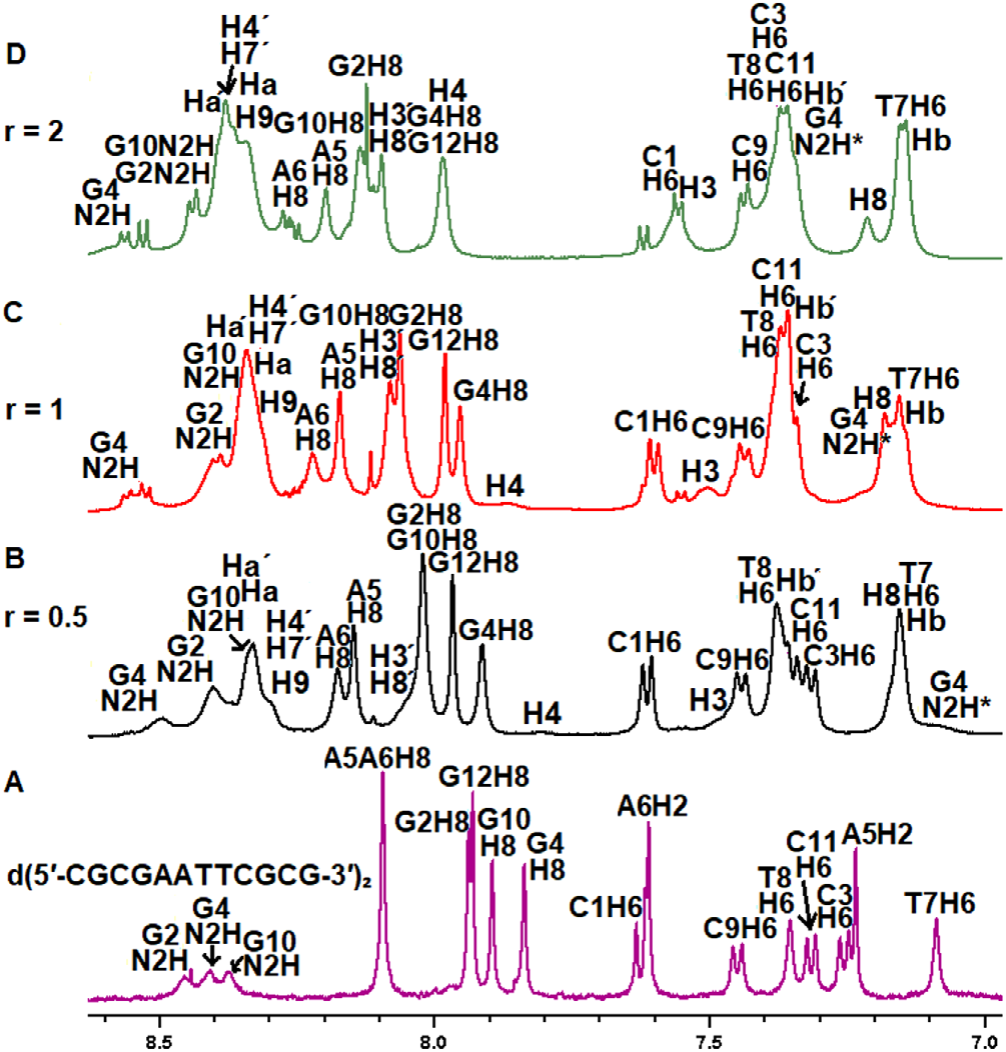
Part of  1H NMR spectra (H_2_O:D_2_O, 9 :1, 298 K, phosphate buffer 100 mM, pH = 7.0) with proton assignments, recorded during the titration of d(5′‐CGCGAATTCGCG‐3′)2 with complex (4a) at three different ratios: a) free oligonucleotide, b) *r* = 0.5, c) *r* = 1, and d) *r* = 2.

a) Changes in the proton signals of the complexes (4a)–(6a) (Figure 6)
  Titration of d(5′‐CGCGAATTCGCG‐3′)_2_ with complex (**4a**) induces pronounced changes in the NMR spectra. At a molar ratio of *r* = 0.5, the proton signals of both **phe** and **bq** exhibit significant broadening, upfield shifts, and splitting into multiple signals. Notably, **bq**H2 resolves into three separate upfield‐shifted signals at about 0.48, 0.14, and 0.13 ppm, indicating the existence of three chemically distinct environments. The remaining protons of the **bq** follow similar trends, with **bq**(H3, H8, H4, H7) showing upfield shifts of up to 0.50 ppm, when observable. The magnitude of these shifts, along with the intermediate exchange kinetics observed, suggest that **bq** engages in at least two binding modes with the oligonucleotide duplex. The species corresponding to the upfield shift of 0.50 ppm is consistent with intercalative binding, whereas the signals corresponding to 0.10 and 0.14 ppm upfield shifts are attributed to groove binding. Similarly, the **phe**H2′H9′ signal splits into three signals, shifted upfield by 0.10, 0.32 and 0.39 ppm, indicative of a binding pattern comparable to that of **bq**.
  Upon the addition of the complex (**5a**), two distinct new upfield signals arise for both the **bq**H2 (0.12 and 0.16 ppm) and **phe**H2′H9′ (0.05 and 0.08 ppm) with a more pronounced shifts observed for **bq** protons. These shifts fall within the range of 0.16–0.30 ppm. However, the **phe**H5′H6′ and **bq**H5H6 of (**5a**), located at the outer face of their aromatic ring system, shifted upfield by 0.25–0.30 ppm. The magnitude of these shifts suggest that (**5a**) interacts with the oligonucleotide through both **phe** and **bq** moieties, likely involving the front face of their ring system. These chemical shift changes are comparable to those observed for complex (**4a**), further supporting a groove‐binding interaction.
  Similarly to complex (**5a**), titration with (**6a**) results in two distinct sets of signals for most proton signals, along with broadening and upfield shifts of the **bq**H2 and **phe**H2′H9′. As before, the most pronounced changes are observed for the **phe** and **bq** protons, indicating their involvement in the binding with the oligonucleotide. However, unlike complex (**5a**), which interacts through the front face of the **phe** and **bq**, complex (**6a**) appears to engage the oligonucleotide through the lateral side of their aromatic ring system, specifically the ring bearing the nitrogen heteroatom. This is supported by the larger shifts, observed for the H3′H8′ (0.29 ppm) and H4′H7′ (0.35 ppm) of **phe**, and H3′ (0.41 ppm) of **bq**. Notably, these shifts are slightly more pronounced than those induced by (**5a**) but remain smaller than those observed for (**4a**).
  In conclusion, all three chloride salts of the complexes (**4**)–(**6**) bind to the oligonucleotide duplex through their **bq** and **phe** ligands, by multiple binding modes. Similar conclusions were drawn from the interaction of the analogous symmetric complexes {[(η⁶‐p‐cym)Ru(phe)]_2_(μ‐BL‐i)}^4+^, with the same oligonucleotide sequence. In that case, both **phe** ligands participated in the binding, each interacting with the d(5′‐CGCGAATTCGCG‐3′)_2_ in a distinct manner^[^ ^73^ ^]^. The binding of (**4a**)–(**6a**) induced conformational changes in the complexes, evidenced by moderate upfield shifts in both the BLs and cymene moieties.
b) Changes in the signals of the nonexchangeable protons of d(5′‐CGCGAATTCGCG‐3′)_2_ (Figure 7):
  Upon addition of (**4a**) to the d(5′‐CGCGAATTCGCG‐3′)_2_ at *r* = 0.5, downfield shifts of up to 0.14 ppm (e.g., **G10**H8) were observed for the guanine H8 proton signals. Additionally, the signals of **C3**H6, **T7**H6, and the **A(5**,**6)**H8, which are no longer chemically equivalent, showed downfield shifts > 0.05 ppm suggesting a base‐destacking effect of the oligonucleotide helix. Given that intercalation of a molecule between stacked base pairs typically disrupts base stacking by forcing them apart to accommodate the intercalator,^[^ ^95^ ^]^ these observations support the hypothesis that one of the aromatic rings (**bq** or **phe)** of (**4a**) intercalates between the central base pairs of the sequence. This intercalation likely occurs via the minor groove, as evidenced by significant broadening or upfield shifts of protons located within the minor groove, such as **A5**H2 and **A6**H2. Additionally, the sugar protons **G4**H1′ and **A5**H1′ exhibited notable upfield shifts (0.17 ppm and 0.19 ppm, respectively), while **A5**H4′ also shifted upfield by 0.10 ppm, further supporting the involvement of the minor groove in the binding interaction. Assuming that the chelating ligand on the one metal center in complex (**4a**) interacts with the central part of the sequence, and considering that, (i) the distance separating it from the second metal center is about 10 Å,^[^ ^73^ ^]^ and (ii) the average rise between adjacent base pairs in the helix is approximately 3.4 Å,^[^ ^96^ ^]^ the second metal center could be positioned either in the G2C3G4 region (toward the 5′ end) or in the T7T8C9 region (toward the 3′ end). NMR data support both possibilities, as significant proton signals changes are observed in bases in both regions. These include downfield shifts of **T7**CH_3_ and **T8**CH_3_ (0.09 and 0.13 ppm, respectively), **C3**H5 (0.15 ppm), **C9**H5 (0.24 ppm), as well as shifts in **G2**H2′ (–0.13 ppm) and **C3**H2′ (+0.17 ppm), suggesting that both regions are involved in the binding interaction. Increasing the ratio notable downfield shifts were observed for the bases’ aromatic protons following the initial trends for the binding mode.
  The interaction of (**5a**) with the d(5′‐CGCGAATTCGCG‐3′)_2_ differs significantly from that of (**4a**), as it induces considerably smaller downfield shifts in the aromatic proton signals of the oligonucleotide bases (0.02–0.06 ppm), which are localized at the sequence termini, specifically in the G2C3G4 and C9G10C11 regions (Tables S7). At higher molar ratios, further shifts of these aromatic proton signals (H6/H8) were observed. In particular, the pronounced downfield shifts of **C3**H5 and **C9**H5 (0.09 ppm) support interaction of (**5a**) at these sites. Given the substantial spatial separation between the two regions, it is unlikely that a single (**5a**) molecule could simultaneously interact with both. This suggests that at least two (**5a**) molecules bind independently to the G2C3G4 and C9G10C11 segments at the sequence ends. Additionally, moderate upfield shifts (0.05 ppm) of the (**G2, C11, G12)** H1′ (*r* = 0.5) further support a minor groove binding mode for (**5a**).
  Finally, the addition of complex (**6a**) to d(5′‐CGCGAATTCGCG‐3′)_2_ induces significant changes in the oligonucleotide's proton signals. Downfield shifts in the aromatic H6/H8 proton signals of **G2**, **C3**, **G10**, and **C11** were observed, ranging from 0.06 to 0.12 ppm (*r* = 2), while the corresponding protons of the remaining bases exhibited only minor shifts (<0.04 ppm) (Table S9). Notably, from *r* = 1 onward, the **A5**H8 and **A6**H8 signals lost their chemical equivalence, suggesting that (**6a**) binds at the central region of the duplex. Additional downfield shifts were observed for the **C3**H5 and **C11**H5 (0.26 and 0.19 ppm, respectively), further supporting binding of (**6a**) within the central segment of the sequence, extending toward the G2C3 and G10C11 bases of the sequence. Significant upfield shifts were also observed in the H1′ sugar proton signals, particularly those of terminal bases, consistent with minor groove interaction in these regions. In some cases, the shifts exceeded 0.25 ppm, as seen for **G2**H1′ (0.28 ppm) and **G12**H1′ (0.27 ppm). Upfield shifts ranging from 0.10 to 0.16 ppm were also observed for the H4′ sugar protons of **G4**, **A5**, **A6**, **G10**, and **G12**, suggesting that the complex extends along the minor groove of the duplex. Similar NMR perturbations have been reported for the interaction of d(5′‐CCGAGAATTCCGG‐3′)_2_ with dinuclear ruthenium complexes, which have been attributed to minor groove binding mode^[^ ^97^ ^]^.
c) Changes in the signals of the exchangeable amino and imino protons of d(5′‐CGCGAATTCGCG‐3′)_2_:
  Exchangeable imino and amino protons of DNA bases can be detected in an H_2_O:D_2_O (9:1) solvent mixture, and their chemical shifts provide information about the Watson–Crick (W.‐C.) hydrogen bonds between DNA strands. Specifically, the (**G10**, **G4**, **G2**)NH1 participate in hydrogen bonds with the nitrogen atoms of (**C3, C9, C11**)N1, respectively, while the (**T8, T7**)N3H form hydrogen bonds with the nitrogen atom (**A5**, **A6**)N1. The terminal bases of the sequence, **C1** and **G12** undergo fast proton exchange with solvent rendering their imino and amino protons are not observable at 298 K^[^ ^98^ ^]^. Additionally, hydrogen bonds are formed by one proton (H*) of the exocyclic amino group of guanines (**G10**, **G4**, **G2**)N2H or cytosines (**C3, C9, C11**)N4Η with the carbonyl groups of cytosines (**C3, C9, C11**)O2 or guanines (**G10**, **G4**, **G2**)O6, respectively, (Figure 5).

**Figure 8:**
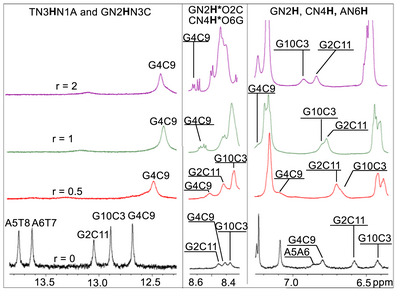
Regions of 1H NMR spectra of the mixtures of (4a) and the d(5΄‐CGCGAATTCGCG‐3΄)2 at *r* = 0, 0.5, 1 and 2, showing the imino and amino protons of the bases with proton assignments (H_2_O:D_2_O, 9:1, phosphate buffer 100 mM, pH = 7.0, 298 K).

Upon addition of (**4a**) to d(5′‐CGCGAATTCGCG‐3′)_2_ at *r* = 0.5, the signals corresponding to the hydrogen bonded (**T8, T7**)N3H as well as (**G2, G10**)N2H, are significantly broadened or nearly disappear. At *r* = 1, this effect extends to the hydrogen bonded amino protons of (**G2, G10**)N2H* or (**C11, C3**)N4H*. In contrast, the signals of **G4**N1H, **G4**N2H*, and **C9**N4H* remain largely unaffected, showing only marginal chemical shift changes (Figure 8). Significant downfield shifts are observed for the nonhydrogen‐bonding amino protons of (**G2**, **G4**, **G10**)N2H/(**C3, C9, C11**)N4Η. Together, these findings are consistent with a localized disruption of W.‐C. hydrogen bonds at the central region of the duplex, specifically in the sequence A5A6T7T8. In particular, the pronounced upfield shift of the **G4**N1H imino proton (0.36 ppm at *r* = 2) indicates a weakening and elongation of the hydrogen bond in which it participates. However, this hydrogen bond remains intact between the **G4·C9** base pair, continuing to stabilize the duplex by holding the two strands together.

**Figure 9:**
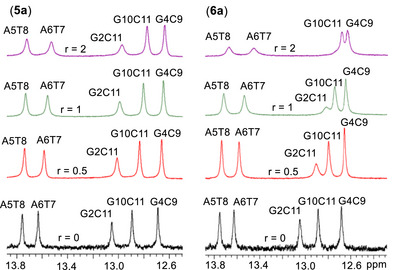
Imino protons regions of the ^1^H NMR spectra (H_2_O : D_2_O, 9 : 1, 298 K, phosphate buffer 100 mM, pH = 7.0) with proton assignments, recorded during the titration of d(5′‐CGCGAATTCGCG‐3′)2 with complexes (5a) and (6a) at three different ratios *r*.

The addition of complex (**5a**) induces comparatively smaller changes in the imino and amino oligonucleotide proton signals exhibiting moderate upfield shifts, indicating that these hydrogen bonds are not disrupted. At *r* = 0.5, slight upfield shifts were observed for **T7**N3H and **G10**N1H (+0.03 and + 0.04 ppm, respectively), while the hydrogen‐bonded **G10**N2H* or **C3**N4H* shifted downfield by 0.07 ppm (Table S11). At r = 1, further perturbations were observed across all imino protons, with those of the central base pairs (**G4·C9** and **A5·T8**) being the least affected, suggesting a binding preference at the terminal regions of the duplex rather than the center. At *r* = 2, broadening of the **G2**N1H and **T8**N3H **T7**N3H imino signals became evident, indicative of intermediate exchange kinetics, consistent with a groove‐binding interaction (Figure 9). Notably, an upfield shift of –0.16 ppm for **G12**H1′ further supports duplex perturbation at the terminal regions.

In the case of (**6a**) significant changes were observed primarily for the imino proton signals, as well as for the N2H*, involved in W.‐C. base pairing. At a ratio of *r* = 0.5, the **G2**N1H and **G10**N1H exhibited the most pronounced upfield shifts (0.13 and 0.08 ppm, respectively), indicating an initial binding preference at the duplex termini. As the concentration of (**6a**) increased, these shifts continued to increase (up to 0.30 ppm), while in addition upfield shift of 0.15 ppm was observed for the **Τ7**N3H corresponding to the **A6·T7** base pair. These observations confirm that (**6a**) engages both the terminal and central regions of the d(5′‐CGCGAATTCGCG‐3′)_2_. In addition to these chemical shift changes, pronounced signal broadening was observed for the **G2**N1H **Τ7**N3H and **Τ8**N3H, suggesting significant elongation of the corresponding hydrogen bonds and potential local unwinding, particularly at the duplex terminus where the **G2**N1H signal nearly disappears at *r* = 2. (Figure 9). Furthermore, all N2H and N2H* signals showed notable downfield shifts of up to 0.26 ppm, indicating substantial perturbation of the helical structure.

Taken together, the NMR data indicate that complexes (**4a**)–(**6a**) bind to the d(5′‐CGCGAATTCGCG‐3′)_2_ through their chelating ligands, either simultaneously through both metal centers or independently through one of the ligands. Complex (**4a**) binds at the central region of the duplex through intercalation from the minor groove, leading to local base‐pair disruption and partial helix unwinding. Although less common than major groove intercalation, minor groove intercalation is a structurally supported mode of DNA binding. For instance, the ruthenium complex Δ‐[Ru(phe)_2_dpq]^2^⁺ (dpq = dipyrido[3,2‐d:2′,3′‐*f*]quinoxaline) has been shown by NMR spectroscopy to intercalate into the hexanucleotide d(5′‐GTCGAC‐3′)_2_ via the minor groove^[^ ^99^ ^]^. Likewise, dinuclear Ru(II) complexes such as Λ, Λ‐[μ‐(11,11′‐bidppz)(phe)_2_Ru_2_]^4+^ (11,11′‐bidppz = 11,11′‐bis(dipyrido[3,2‐*a*:2′,3′‐*c*]phenazinyl)) have been reported to deeply intercalate through the minor groove^[^ ^100^ ^]^. A comparison between complex (**4a**) and complex (**A**) reveals that both interact with the d(5′‐CGCGAATTCGCG‐3′)_2_ through similar binding modes, initially targeting the central region and extending in both directions along the helix^[^ ^73^ ^]^. However, despite this common binding motif, the two complexes differ substantially in their impact on duplex stability. NMR data show that (**4a**) induces more pronounced upfield shifts, up to 0.17 ppm, for aromatic protons H6/H8 of the nucleobases, compared to those observed with complex (**A**). Moreover, while the imino proton signals remain clearly resolved up to *r* = 1 in the presence of (**A**), they almost disappear as early as *r* = 0.5 in the case of (**4a**), indicating greater disruption of base pairing and helical structure (Figure S52). These observations indicate that (**4a**) destabilizes the duplex at lower ratios than (**A**), despite its lower positive charge (+3 vs. +4). Even though electrostatic attraction typically favors higher‐charged species, stronger DNA binding is not necessarily correlated with charge alone. Previous studies have shown that lower‐charged metal complexes can exhibit comparable or even enhanced intercalative binding. Ahmad et al. demonstrated that structurally similar metal complexes differing only in charge can switch between groove binding and intercalation, with the lower‐charged analogues displaying stronger intercalative behavior^[^ ^101^ ^]^. Similarly, Thomas and coworkers reported that tetracationic Ru(II) polypyridyl complexes preferentially bind in the groove, whereas dicationic counterparts intercalate and induce greater DNA structural perturbations^[^ ^102^ ^]^. In our previous work, we also have suggested that sizable multicharged cationic complexes can exhibit strong DNA affinity driven largely by electrostatic interactions^[^ ^73^ ^]^. However, even though these studies offer valuable insights, they are primarily based on spectrophotometric or fluorescence data using calf thymus DNA, which lack sequence specificity and cannot identify precise binding sites and modes. In contrast, detailed NMR investigations, such as those presented in the present study, provide atomic‐level resolution, enabling unambiguous elucidation of DNA binding modes and structural consequences.

In complex (**5a**) the two metal centers are connected through a longer and more flexible BL than (**4a**), which potentially allows the complex to partially adapt to the local geometry of the d(5′‐CGCGAATTCGCG‐3′)_2_ access to distinct binding sites. Related studies on dinuclear ruthenium complexes have demonstrated that linker flexibility and length can significantly influence groove recognition and binding mode^[^ ^103^ ^]^. In the case of (**5a**), NMR data sets that two distinct molecules bind independently at opposite termini of the DNA duplex, engaging the G2C3G4 and C9G10C11 regions, but orient toward the sequence center. This arrangement allows them to induce perturbations that extend beyond the primary binding sites, affecting both the sugar‐phosphate backbone and base pairing along the helix. The observed pattern of H1′ upfield shifts, combined with the downfield shifts of H6/H8 and N2H* signals, points to interactions involving both the minor and major groove.

Similarly, two molecules of complex (**6a**) bind to the d(5′‐CGCGAATTCGCG‐3′)_2_ oligonucleotide simultaneously, each initiating at opposite ends of the helix and extending toward the center. The observed chemical shift changes and signal broadening suggest interactions involving both the major and minor grooves, with localized base pair disruption and partial destabilization at the termini and the central A·T‐rich region. Unlike BL‐2 in (**5a**), the BL‐3 BL in complex (**6a**) provides greater conformational freedom, enabling better adaptation of the two ruthenium centers to the DNA duplex supporting interaction with both grooves. This increased flexibility likely explains the more pronounced shift changes and signals broadening observed in the NMR spectra of (**6a**), particularly in regions proximal to the binding sites. These findings are in line with previous reports on dinuclear ruthenium complexes, where flexible linkers were shown to promote effective groove binding by allowing each metal center to accommodate in local DNA topology^[^ ^104^ ^]^.

A comparison between complexes (**6a**) and (**C**) reveals similar binding modes and targeted regions of the sequence d(5′‐CGCGAATTCGCG‐3′)_2_. Following the same trend observed for (**4a**), complex (**6a**), despite its lower overall charge (3+) compared to (**C**), induces more pronounced shift changes in the oligonucleotide proton signals (Figure S63). By contrast, the comparison between complexes (**5a**) and (**B**) reveals a more balanced interaction pattern. Although the tetracationic (**B**) induces slightly higher shifts in most of the oligonucleotide's proton signals, the difference in spectral perturbation compared to the tricationic (**5a**) is relatively modest. Notably, several aromatic protons, such as the H8 signals of G2, A5, and A6, are affected to a similar level by both complexes (Figure S62).

### 2.5 Fluorescence Quenching Studies on the Interactions of Complexes (**1a**)–(**6a**) With the DNA Oligonucleotide Duplex D(5′‐CGCGAATTCGCG‐3′)_2_

The results of the fluorescence quenching study, performed to determine the binding parameters for complexes (**1a**)–(**6a**) with the oligonucleotide d(5′‐CGCGAATTCGCG‐3′)_2_, are summarized in Table 2.

**Table 2:** DNA binding parameters for complexes (1a)–(6a) with the DNA oligonucleotide d(5΄‐CGCGAATTCGCG‐3΄)2. For comparison, the binding parameters of the complexes (A)–(C) are also presented 73.

Complexes	*K*_SV_ (× 10^4^ M^−1^)	*K*_b_ (× 10^3^ M^−1^)
(**1a**)	2.314 ± 0.215	807.6 ± 19.9
(**2a**)	1.418 ± 0.352	298.4 ± 4.3
(**3a**)	0.331 ± 0.064	602.8 ± 76.1
(**4a**)	0.690 ± 0.040	5.59 ± 0.21
(**5a**)	0.904 ± 0.065	28.51 ± 0.97
(**6a**)	0.296 ± 0.049	6.70 ± 0.58
(**A**)	16.65 ± 0.080	12.13 ± 0.01
(**B**)	0.286 ± 0.018	2.333 ± 0.01
(**C**)	0.273 ± 0.027	3.336 ± 0.01

Upon addition to DNA, the fluorescence intensity of the mixture increases progressively until all available binding sites are saturated^[^ ^105^ ^]^. When a DNA‐binder is added to the system and acts competitively, it displaces EB from the DNA, resulting in a decrease in fluorescence intensity (Figure S64). The values of the Stern–Volmer quenching constant (*K*_SV_) were calculated from the slope of the plots *F*_0_/*F* = *f*[*Q*] (Figure S65). Comparing complexes (**1a**–**6a**) with each other, as well as with the tetracationic complexes (**A**)–(**C**), we can conclude that all complexes with BL‐3 have very low *K*_SV_ values in comparison to their BL‐1 and BL‐2 counterparts for all the possible complex charges. For the three complexes bearing BL‐1 the highest *K*_SV_ value is observed for the (**Α**), significantly higher among all those complexes. Finally, for the complexes bridged by BL‐2, the lowest *K*_SV_ value was calculated for the + 4 complex, while for the + 3 and + 2 complexes comparable *K*_SV_ values were obtained.

The binding constant (*K*_b_) for each complex was estimated from the fluorescence titration data using the double‐log plot log(*F*_0_‐*F*/*F*) = *f*(log[*Q*]) (Figure S66). All three + 2 complexes, (**1a**)–(**3a**), exhibited higher *K*_b_ values compared to the + 3, (**4a**)–(**6a**), and + 4, (**A**)–(**C**) counterparts, indicating stronger binding affinities. Among the complexes containing the BL‐1 (**A**) and (**4a**) showed comparable *K*_b_ values, while (**1a**) exhibited significantly higher *K*_b_. In the case of BL‐2 bridged series, a clear trend emerged: the binding affinity increased as the overall charge decreased. Specifically, the complex (**5a**) displayed a *K*_b_ value approximately one order of magnitude higher, compared to tetracationic complex (**B**), and similarly, the dicationic complex (**2a**) surpassed complex (**5a**) in binding strength.

Taken together, these fluorescence results clearly demonstrate that the binding affinity of the complexes is inversely correlated with their overall charge and is influenced by the nature and length of the BL. In addition to supporting these affinity trends, the fluorescence displacement experiments with EB further support the proposed binding modes. Since EB can act both as an intercalator^[^ ^106^ ^]^ and as a groove binder,^[^ ^107^ ^]^ its displacement pattern confirms that complex (**4a**) binds to DNA through intercalation, whereas complexes (**5a**) and (**6a**) interact with DNA through the minor groove.

### 2.6 Cytotoxic Activity

The complexes (**1a**)–(**3a**) showed IC_50_ values ​​in the range 5–10 µM against A2780, and 7–15 µM against A2780cisR cell line, slightly lower than their + 3 charged analogues (**4a**)–(**6a**), indicating that decrease of the overall charge from + 3 to + 2 exerts minimal influence on cytotoxicity. In contrast, their + 4 analogues (**A**)–(**C**) exhibited reduced cytotoxicity (IC_50_ = 35–200 µM)^[^ ^73^ ^]^ suggesting that increasing the charge beyond + 3, exerts negative effect on cytotoxic activity, probably due to reduced cellular uptake.

Nevertheless, the low cytotoxicity of the complexes with a 4 + charge is accompanied by low toxicity to healthy NIH‐3T3 cells (IC_50_ = 150–200 µM) as well, yielding (SI) slightly higher than (**1a**)–(**6a**). Also, of interest is the correlation of cytotoxicity with BL length, where it seems not to differ significantly although in general complexes bridged with BL1 and BL3 show better cytotoxicity than BL2. As the length differences of BL are limited to 1–2 Å, long‐range DNA damages should be sought at a longer BL lengths. Notably, the + 4 charged complexes also showed reduced toxicity toward healthy NIH‐3T3 fibroblasts (IC_50_ = 150–200 µM), with SI values up to 6. In contrast, the more cytotoxic + 2/+3 complexes exhibited reduced selectivity, underscoring a balance between cytotoxicity and selectivity across this series. In the context of the present study, at Ru─Ru separations of 10–12 Å, cytotoxicity appears to correlate with the intermetallic distance, with shorter Ru─Ru separations associated with higher activity. The Ru─Ru distance in BL‐3 lies between those of BL‐1 and BL‐2, as confirmed by crystallographic data.

In the MCF‐7 breast cancer cells, +4 charged complexes (**A**)–(**C**) were largely inactive (IC_50_ > 300 µM; SI ≈ 1–2), whereas complexes (**1a**)–(**6a**) demonstrated potent activity (IC_50_ = 8–37 µM), with SI values comparable to the + 4 series. Notably, the complexes (**1a**) and (**4a**) bridged by BL‐1, showed lower IC_50_ values than cisplatin in both MCF‐7 and A2780cisR cells. In addition, the complexes (**1a**) and (**4a**) bridged by BL‐1, showed lower IC_50_ values than cisplatin in MCF‐7 as well as in A2780cisR cells. The findings are summarized in Table 3.

**Table 3:** IC50 (µM) values selectivity and resistance factor for (1a)–(6a) and (A)–(C) taken from the literature^[^ ^73^ ^]^

Complexes	A2780	A2780cis‐res	MCF‐7	NIHT‐3T3	Selectivity index^[a]^	Resistant factor^[b]^
(**1**)	7.52 ± 0.34	7.58 ± 0.83	8.53 ± 1.03	18.69 ± 3.62	2.5 ‐ 2.5 ‐ 2.2	1
(**2**)	10.81 ± 3.22	14.64 ± 0.47	14.73 ± 1.65	34.75 ± 2.09	3.2 ‐ 2.4 ‐ 2.4	1.4
(**3**)	5.63 ± 1.28	11.83 ± 1.42	18.22 ± 1.30	14.54 ± 0.61	2.6 ‐ 1.2 ‐ 0.8	2.1
(**4**)	10.18 ± 0.76	9.76 ± 0.82	11.19 ± 1.24	17.22 ± 1.22	1.7 ‐ 1.8 ‐ 1.5	1
(**5**)	10.91 ± 2.93	27.66 ± 2.28	37.59 ± 8.87	46.32 ± 5.59	4.2 ‐ 1.7 ‐ 1.2	2.5
(**6**)	8.82 ± 1.35	17.64 ± 3.32	16.42 ± 1.04	25.14 ± 1.51	2.9 ‐ 1.4 ‐ 1.5	2.0
(**A**)	117.3 ± 26.92	193.0 ± 33.15	302.7 ± 26.62	192.3 ± 25.38	1.6 ‐ 1.0 ‐ 0.6	1.6
(**B**)	35.57 ± 9.75	46.00 ± 6.13	275.8 ± 37.43	137.6 ± 18.45	3.9 ‐ 3.0 ‐ 0.5	1.3
(**C**)	34.77 ± 1.71	47.42 ± 3.51	337.5 ± 0.16	184.9 ± 13.07	5.3 ‐ 4.0 ‐ 0.5	1.4
cisplatin	2.54 ± 0.11	18.43 ± 1.59	14.62 ± 1.25	3.02 ± 0.36	1.2 ‐ 0.2 ‐ 0.2	7.3

^[a]^ Selectivity Index = IC_50_(NIHT‐3T3)/IC_50_(MCF‐7)–IC_50_(NIHT‐3T3)/IC_50_(A2780)–IC_50_(NIHT‐3T3)/IC_50_(A2780cis‐res).

^[b]^ Resistance Factor = IC_50_ (A2780cisR)/IC_50_(A2780)

Taken together, these findings place the studied series of (**1a**)–(**6a**) above the average reported in the literature for this type of complexes with saturated of the ruthenium coordination sphere with kinetically inert ligands, typically results in similar cytotoxic profiles^[^ ^64^, ^68^ ^]^. Although certain complexes with lower IC_50_ and limited selectivity have been described,^[^ ^69^ ^]^ others have achieved higher selectivity despite their moderate cytotoxicity^[^ ^50^ ^]^. However, the most notable cytotoxicity remains that of trithiolato‐bridged, with IC_50_ values in the low nM scale^[^ ^70^ ^]^.

In general, bifunctional and monofunctional dinuclear Ru(II)‐arene complexes interact covalently with the biomolecular targets following the hydrolysis of their chloride ligands^[^ ^34^, ^37^ ^]^. In all cases, they exhibit enhanced cytotoxic activity across various cancer cell lines compared to their mononuclear analogues^[^ ^31^, ^32^, ^33^, ^34^, ^35^, ^36^, ^37^, ^38^, ^39^, ^40^, ^41^, ^42^, ^43^, ^44^, ^45^, ^46^, ^47^, ^48^, ^49^, ^50^, ^51^, ^52^, ^53^, ^54^, ^55^, ^56^, ^57^, ^58^, ^59^, ^60^ ^]^ a trend often attributed to the increased lipophilicity provided by the bridging linker^[^ ^36^ ^]^. In some cases, however, their cytotoxicity is associated with their ability to form crosslinks with biological macromolecules.

Bifunctional complexes generally display significantly higher cytotoxicity^[^ ^31^, ^32^, ^33^, ^34^, ^35^, ^36^, ^37^ ^]^ than the monofunctional,^[^ ^38^, ^39^, ^43^, ^44^, ^45^, ^46^, ^47^, ^48^, ^57^ ^]^ whose activity is often moderate^[^ ^49^, ^50^, ^51^, ^52^, ^53^, ^54^, ^55^, ^56^, ^58^ ^]^ or even low^[^ ^57^, ^58^, ^59^ ^]^. However, dinuclear complexes forming noncovalent DNA adducts typically exhibit low cytotoxicity but notable selectivity^[^ ^62^, ^73^ ^]^. In contrast, those containing trithiolato BLs show high cytotoxicity but lack selectivity in most cases^[^ ^66^, ^67^, ^68^, ^69^, ^70^, ^71^, ^72^ ^]^.

Interestingly, studies performed on both cisplatin‐sensitive and cisplatin‐resistant cell lines (e.g., A2780/ A2780cis‐res ^[^ ^31^, ^32^, ^35^ ^]^ and MCF‐7/MCF‐7cis‐res^[^ ^34^ ^]^) revealed comparable activity of the dinuclear complexes in both models. This may suggest the ability of these complexes to bypass several resistance mechanisms, such as reduced cellular uptake, enhanced DNA repair enzyme activity, or pump‐out the complex through enzyme‐mediated pathways (e.g., glutathione‐ and methionine‐related pumps). With respect to DNA repair, both the extended spacing between DNA binding sites and the strength of DNA adduct formation, may impair the efficiency of the cellular repair machinery,^[^ ^76^ ^]^ providing an additional basis for the potency of dinuclear over mononuclear complexes.

## 3 Conclusion

In this work, we synthesized and comprehensively characterized a series of dinuclear η⁶‐arene Ru(II) complexes incorporating benzo[h]quinoline (bq) and/or 1,10‐phenanthroline (phe) ligands, bridged by bis‐pyridyl linkers of varying length. This library of dinuclear Ru(II) arene complexes, spanning overall charges of + 4 (A–C),^[^ ^73^ ^]^ +3 (4–6), and + 2, ^[^ ^1^, ^2^, ^3^ ^]^ enabled systematic evaluation of how charge and Ru–Ru separation modulate DNA binding and cytotoxic activity.

NMR data reveal multiple binding modes, partial intercalation of phe or bq ligands and minor‐groove binding, sometimes accompanied by localized helix unwinding. Tricationic complexes (4)–(6) induce stronger DNA perturbations than the tetracationic analogues (A)–(D), ^[^ ^73^ ^]^ highlighting the influence of charge on binding strength. Fluorescence quenching experiments yielded Stern–Volmer and binding constants consistent with moderate‐to‐high affinities, confirming the NMR trends and the dependence on ligand nature and linker length.

Cytotoxicity studies demonstrated that complexes (1a)–(6a) exhibit micromolar IC_50_ values against MCF‐7, A2780, and A2780cis‐resistant cell lines, with some degree of selectivity towards cancer cells over normal fibroblasts.

It is important to note that the cytotoxicity of these complexes does not arise from direct coordination of the metal with biomolecules, since the ruthenium centers are fully saturated with kinetically inert ligands. Their biological activity could possibly arise from reversible ligand interactions which, in the case of DNA, induce distortions of the helix that may play a crucial role in triggering cell apoptosis^[^ ^75^, ^108^, ^109^ ^]^. However, the possibility of interactions with other biomolecules contributing to cell death cannot be excluded. These findings point to a distinct mechanism of action for metal‐based drugs, expanding the paradigm beyond direct metal coordination.

## Supporting Information

The authors have used additional citations within the Supporting Information, which are referenced on the “References” segment of the main text.

## Conflict of Interest

The authors declare no conflict of interest.

## Supporting information



Supporting Information

Supporting Information

## Data Availability

The data that support the findings of this study are available from the corresponding author upon reasonable request.
