# Supplementary material for: DNA Noncovalent Interactions of Dinuclear η6‐Arene Ru(II) Complexes: Influence of Complex Charge and Bridging Ligand Length on DNA Binding Mode and Cytotoxic Activity
Source: Chemistry. 2025 Nov 10;31(71):e02680. doi: 10.1002/chem.202502680 (PMC12734683; doi:10.1002/chem.202502680)
Supplement: Supplementary file 1 — Supporting Information [file CHEM-31-e02680-s001.docx]

**SUPPLEMENTARY MATERIAL**

**DNA Non-Covalent Interactions of Dinuclear η^6^-Arene Ru(II) Complexes: Influence of Complex Charge and Bridging Ligand Length on DNA Binding Mode and Cytotoxic Activity**

Dimitrios Thomos^[a]^, Theodoros Tsolis* ^[a]^, John C. Plakatouras^[a]^, Ioannis-Michail Chronakis^[a],[b]^, Angeliki Magklara^[b]^ and Achilleas Garoufis* ^[a]^

[a] D. Thomos, Dr. T. Tsolis, Prof. J.C. Plakatouras, I.-M. Chronakis, Prof. A. Magklara, Prof. A. Garoufis

Department of Chemistry, Laboratory of Inorganic Chemistry,
University of Ioannina
GR-45110 Ioannina, Greece
E-mail: [agaroufi@uoi.gr](mailto:agaroufi@uoi.gr) (A. Garoufis), [t.tsolis@uoi.gr](mailto:t.tsolis@uoi.gr) (T. Tsolis)

[b] I.-M. Chronakis, Prof. A. Magklara,

Department of Clinical Chemistry, Faculty of Medicine, School of Health Sciences Ioannina, Greece
Institution, University of Ioannina,
GR-45110 Ioannina, Greece

Biomedical Research Institute-Foundation for Research and Technology,

GR-45110 Ioannina, Greece

Institute of Biosciences, University Research Center of Ioannina (URCI),

GR-45110 Ioannina, Greece

**Table of Contents**

**S1. Experimental**

S1.1. Materials and methods

S1.2. Crystal structure determination

S1.3. Interactions of (4a) (5a) and (6a) with the oligonucleotide d(5′-CGCGAATTCGCG-3′)_2_.

S1.4. ^1^H NMR assignments of d(5′-CGCGAATTCGCG-3′)_2_.

S1.5. Fluorescence measurements

S1.6. Cell culture

S1.7. Cell growth assay

S1.8. Synthesis of the complexes

**Figure S1**: Part of HR-ESI-MS of the complex (**1**) in acetonitrile at 298 K.

**Figure S2**: Part of HR-ESI-MS of the complex (**2**) in acetonitrile at 298 K.

**Figure S3**: Part of HR-ESI-MS of the complex (**3**) in acetonitrile at 298 K.

**Figure S4**: Part of HR-ESI-MS of the complex (**4**) in acetonitrile at 298 K.

**Figure S5**: Part of HR-ESI-MS of the complex (**5**) in acetonitrile at 298 K.

**Figure S6:** Part of HR-ESI-MS of the complex (**6**) in acetonitrile at 298 K.

**Figure S7**: ^1^H NMR spectrum of the complex (**1**) in acetone-d6 at 298 K.

**Figure S8**: ^1^H NMR spectrum of the complex (**2**) in acetone-d6 at 298 K.

**Figure S9**: ^1^H NMR spectrum of the complex (**3**) in acetone-d6 at 298 K.

**Figure S10**: ^1^H NMR spectrum of the complex (**4**) in acetone-d6 at 298 K.

**Figure S11**: ^1^H NMR spectrum of the complex (**5**) in acetone-d6 at 298 K.

**Figure S12**: ^1^H NMR spectrum of the complex (**6**) in acetone-d6 at 298 K.

**Figure S13**: ¹H–¹H COSY NMR spectrum of complex (**1**) in acetone-d₆ at 298 K, showing proton–proton cross-peak assignments.

**Figure S14**: ¹H–¹H COSY NMR spectrum of complex (**2**) in acetone-d₆ at 298 K, showing proton–proton cross-peak assignments.

**Figure S15**: ¹H–¹H COSY NMR spectrum of complex (**3**) in acetone-d₆ at 298 K, showing proton–proton cross-peak assignments.

**Figure S16**: ¹H–¹H COSY NMR spectrum of complex (**4**) in acetone-d₆ at 298 K, showing proton–proton cross-peak assignments.

**Figure S17**: ¹H–¹H COSY NMR spectrum of complex (**5**) in acetone-d₆ at 298 K, showing proton–proton cross-peak assignments.

**Figure S18**: ¹H–¹H COSY NMR spectrum of complex (**6**) in acetone-d₆ at 298 K, showing proton–proton cross-peak assignments.

**Figure S19**: ¹H–¹H NOESY NMR spectrum of complex (**1**) in acetone-d₆ at 298 K, showing proton–proton cross-peak assignments. (A) aromatic region; (B) aliphatic region.

**Figure S20**: ¹H–¹H NOESY NMR spectrum of complex (**2**) in acetone-d₆ at 298 K, showing proton–proton cross-peak assignments. (A) aromatic region; (B) aliphatic region.

**Figure S21**: ¹H–¹H NOESY NMR spectrum of complex (**3**) in acetone-d₆ at 298 K, showing proton–proton cross-peak assignments. (A) aromatic region; (B) aliphatic region.

**Table S2**: Interligand NOE cross-peaks were observed between protons of the **bq,** **cym**, and BL ligands in complexes (**1**) – (**3**) in acetone-d₆ at 298 K.

**Figure S22**: ¹H–¹H NOESY NMR spectrum of complex (**4**) in acetone-d₆ at 298 K, showing proton–proton cross-peak assignments. (A) aromatic region; (B) aliphatic region.

**Figure S23**: ¹H–¹H NOESY NMR spectrum of complex (**5**) in acetone-d₆ at 298 K, showing proton–proton cross-peak assignments. (A) aromatic region; (B) aliphatic region.

**Figure S23**: ¹H–¹H NOESY NMR spectrum of complex (**5**) in acetone-d₆ at 298 K, showing proton–proton cross-peak assignments. (A) aromatic region; (B) aliphatic region.

**Figure S24**: ¹H–¹H NOESY NMR spectrum of complex (**6**) in acetone-d₆ at 298 K, showing proton–proton cross-peak assignments. (A) aromatic region; (B) aliphatic region.

**Table S3**: Interligand NOE cross-peaks were observed between protons of the **bq**, **phe**, **cym**, **cym΄**and BL ligands in complexes (**4**) – (**6**) in acetone-d₆ at 298 K.

**Figure S25**: ^1^H NMR spectrum of d(5′-CGCGAATTGGCC-3′)_2_ upon addition of complex (**4a**) at r = 0.5 in H_2_O/ D_2_O 9:1 (buffer phosphate 100 mM, pH = 7.0) at 298 K, 500 MHz.

**Figure S26**: ^1^H–^1^H COSY spectrum of d(5′-CGCGAATTGGCC-3′)_2_ upon addition of complex (**4a**) at r = 0.5 in H_2_O/ D_2_O 9:1 (buffer phosphate 100 mM, pH = 7.0) at 298 K, 500 MHz.

**Figure S27**: ^1^H–^1^H NOESY spectrum of d(CGCGAATTCGCG)_2_ upon addition of complex (**4a**) at r = 0.5 in H_2_O/ D_2_O 9:1 (buffer phosphate 100 mM, pH = 7.0) at 298 K, 500 MHz.

**Figure S28**: ^1^H NMR spectrum of d(5′-CGCGAATTGGCC-3′)_2_ upon addition of complex (**4a**) at r = 1 in H_2_O/ D_2_O 9:1 (buffer phosphate 100 mM, pH = 7.0) at 298 K, 500 MHz.

**Figure S29**: ^1^H–^1^H COSY spectrum of d(5′-CGCGAATTGGCC-3′)_2_ upon addition of complex (**4a**) at r = 1 in H_2_O/ D_2_O 9:1 (buffer phosphate 100 mM, pH = 7.0) at 298 K, 500 MHz.

**Figure S30**: ^1^H–^1^H NOESY spectrum of d(5′-CGCGAATTGGCC-3′)_2_ upon addition of complex (**4a**) at r = 1 in H_2_O/ D_2_O 9:1 (buffer phosphate 100 mM, pH = 7.0) at 298 K, 500 MHz.

**Figure S31**: ^1^H NMR spectrum of d(5′-CGCGAATTGGCC-3′)_2_ upon addition of complex (**4a**) at r = 2 in H_2_O/ D_2_O 9:1 (buffer phosphate 100 mM, pH = 7.0) at 298 K, 500 MHz.

**Figure S32**: ^1^H–^1^H COSY spectrum of d(5′-CGCGAATTGGCC-3′)_2_ upon addition of complex (**4a**) at r = 2 in H_2_O/ D_2_O 9:1 (buffer phosphate 100 mM, pH = 7.0) at 298 K, 500 MHz.

**Figure S33**: ^1^H–^1^H NOSEY spectrum of d(5′-CGCGAATTGGCC-3′)_2_ upon addition of complex (**4a**) at r = 2 in H_2_O/ D_2_O 9:1 (buffer phosphate 100 mM, pH = 7.0) at 298 K, 500 MHz.

**Figure S34**: ^1^H NMR spectrum of d(5′-CGCGAATTGGCC-3′)_2_ upon addition of complex (**5a**) at r = 0.5 in H_2_O/ D_2_O 9:1 (buffer phosphate 100 mM, pH = 7.0) at 298 K, 500 MHz.

**Figure S35**: ^1^H–^1^H COSY spectrum of d(5′-CGCGAATTGGCC-3′)_2_ upon addition of complex (**5a**) at r = 0.5 in H_2_O/ D_2_O 9:1 (buffer phosphate 100 mM, pH = 7.0) at 298 K, 500 MHz.

**Figure S36**: ^1^H–^1^H NOESY spectrum of d(5′-CGCGAATTGGCC-3′)_2_ upon addition of complex (**5a**) at r = 0.5 in H_2_O/ D_2_O 9:1 (buffer phosphate 100 mM, pH = 7.0) at 298 K, 500 MHz.

**Figure S37**: ^1^H NMR spectrum of d(5′-CGCGAATTGGCC-3′)_2_ upon addition of complex (**5a**) at r = 1 in H_2_O/ D_2_O 9:1 (buffer phosphate 100 mM, pH = 7.0) at 298 K, 500 MHz.

**Figure S38**: ^1^H–^1^H COSY spectrum of d(5′-CGCGAATTGGCC-3′)_2_ upon addition of complex (**5a**) at r = 1 in H_2_O/ D_2_O 9:1 (buffer phosphate 100 mM, pH = 7.0) at 298 K, 500 MHz.

**Figure S39**: ^1^H–^1^H NOESY spectrum of d(5′-CGCGAATTGGCC-3′)_2_ upon addition of complex (**5a**) at r = 1 in H_2_O/ D_2_O 9:1 (buffer phosphate 100 mM, pH = 7.0) at 298 K, 500 MHz.

**Figure S40**: ^1^H NMR spectrum of d(5′-CGCGAATTGGCC-3′)_2_ upon addition of complex (**5a**) at r = 2 in H_2_O/ D_2_O 9:1 (buffer phosphate 100 mM, pH = 7.0) at 298 K, 500 MHz.

**Figure S41**: ^1^H–^1^H COSY spectrum of d(5′-CGCGAATTGGCC-3′)_2_ upon addition of complex (**5a**) at r = 2 in H_2_O/ D_2_O 9:1 (buffer phosphate 100 mM, pH = 7.0) at 298 K, 500 MHz.

**Figure S42**: ^1^H–^1^H NOESY spectrum of d(5′-CGCGAATTGGCC-3′)_2_ upon addition of complex (**5a**) at r = 2 in H_2_O/ D_2_O 9:1 (buffer phosphate 100 mM, pH = 7.0) at 298 K, 500 MHz.

**Figure S43**: ^1^H NMR spectrum of d(5′-CGCGAATTGGCC-3′)_2_ upon addition of complex (**6a**) at r = 0.5 in H_2_O/ D_2_O 9:1 (buffer phosphate 100 mM, pH = 7.0) at 298 K, 500 MHz.

**Figure S44**: ^1^H–^1^H COSY spectrum of d(5′-CGCGAATTGGCC-3′)_2_ upon addition of complex (**6a**) at r = 0.5 in H_2_O/ D_2_O 9:1 (buffer phosphate 100 mM, pH = 7.0) at 298 K, 500 MHz.

**Figure S45**: ^1^H–^1^H NOESY spectrum of d(5′-CGCGAATTGGCC-3′)_2_ upon addition of complex (**6a**) at r = 0.5 in H_2_O/ D_2_O 9:1 (buffer phosphate 100 mM, pH = 7.0) at 298 K, 500 MHz.

**Figure S46**: ^1^H NMR spectrum of d(5′-CGCGAATTGGCC-3′)_2_ upon addition of complex (**6a**) at r = 1 in H_2_O/ D_2_O 9:1 (buffer phosphate 100 mM, pH = 7.0) at 298 K, 500 MHz.

**Figure S47**: ^1^H–^1^H COSY spectrum of d(5′-CGCGAATTGGCC-3′)_2_ upon addition of complex (**6a**) at r = 1 in H_2_O/ D_2_O 9:1 (buffer phosphate 100 mM, pH = 7.0) at 298 K, 500 MHz.

**Figure S48**: ^1^H–^1^H NOESY spectrum of d(5′-CGCGAATTGGCC-3′)_2_ upon addition of complex (**6a**) at r = 1 in H_2_O/ D_2_O 9:1 (buffer phosphate 100 mM, pH = 7.0) at 298 K, 500 MHz.

**Figure S49**: ^1^H NMR spectrum of d(5′-CGCGAATTGGCC-3′)_2_ upon addition of complex (**6a**) at r = 2 in H_2_O/ D_2_O 9:1 (buffer phosphate 100 mM, pH = 7.0) at 298 K, 500 MHz.

**Figure S50**: ^1^H–^1^H COSY spectrum of d(5′-CGCGAATTGGCC-3′)_2_ upon addition of complex (**6a**) at r = 2 in H_2_O/ D_2_O 9:1 (buffer phosphate 100 mM, pH = 7.0) at 298 K, 500 MHz.

**Figure S51**: ^1^H–^1^H NOESY spectrum of d(5′-CGCGAATTGGCC-3′)_2_ upon addition of complex (**6a**) at r = 2 in H_2_O/ D_2_O 9:1 (buffer phosphate 100 mM, pH = 7.0) at 298 K, 500 MHz.

**Figure S52**: ^1^H NMR spectrum of the complex (**4a**) in D_2_O at 298 K.

**Figure S53**: ^1^H NMR spectrum of the complex (**5a**) in D_2_O at 298 K.

**Figure S54**: ^1^H NMR spectrum of the complex (**6a**) in D_2_O at 298 K.

**Figure S55**: H5 and H1΄region of ¹H NMR spectra (H_2_O:D_2_O, 9 :1, 298 K, phosphate buffer 100 mM, pH = 7.0) with proton assignments, recorded during the titration of d(5′-CGCGAATTGGCC-3′)_2_ with complex (**4a**) at three different ratios: (A) free oligonucleotide, (B) r = 0.5, (C) r = 1, and (D) r = 2.

**Figure S56**: Part of ¹H NMR spectra (H_2_O:D_2_O, 9 :1, 298 K, phosphate buffer 100 mM, pH = 7.0) with proton assignments, recorded during the titration of d(5′-CGCGAATTGGCC-3′)_2_ with complex (**5a**) at three different ratios: (A) free oligonucleotide, (B) r = 0.5, (C) r = 1, and (D) r = 2.

**Figure S57**: H5 and H1΄region of ¹H NMR spectra (H_2_O:D_2_O, 9 :1, 298 K, phosphate buffer 100 mM, pH = 7.0) with proton assignments, recorded during the titration of d(5′-CGCGAATTGGCC-3′)_2_ with complex (**5a**) at three different ratios: (A) free oligonucleotide, (B) r = 0.5, (C) r = 1, and (D) r = 2.

**Figure S58**: Part of ¹H NMR spectra (H_2_O:D_2_O, 9 :1, 298 K, phosphate buffer 100 mM, pH = 7.0) with proton assignments, recorded during the titration of d(5′-CGCGAATTGGCC-3′)_2_ with complex (**6a**) at three different ratios: (A) free oligonucleotide, (B) r = 0.5, (C) r = 1, and (D) r = 2.

**Figure S59**: H5 and H1΄region of ¹H NMR spectra (H_2_O:D_2_O, 9 :1, 298 K, phosphate buffer 100 mM, pH = 7.0) with proton assignments, recorded during the titration of d(5′-CGCGAATTGGCC-3′)_2_ with complex (**6a**) at three different ratios: (A) free oligonucleotide, (B) r = 0.5, (C) r = 1, and (D) r = 2.

**Table S4** ^1^H NMR chemical shifts of the (**4a**) (H_2_O : D_2_O, 9 : 1, 298 K, buffer phosphates 100 mM, pH = 7.0) free, and upon the addition to the d(5′-CGCGAATTGGCC-3′)_2_ at r = 0.5, 1, and 2. Shifts are denoting in parenthesis (negative sign upfield and positive sign downfield shifts). A, B and C denotes the signals of the three moieties of (**4a**). n.o. = not observed.

**Table S5**: Differences in ^1^H chemical shifts of the d(5′-CGCGAATTGGCC-3′)_2_ (buffer phosphate 100 mM, pH = 7.0) upon the addition of complex (**4a**) in various [Ru]/nucleotide ratios at 298 K, 500 MHz. Values in parenthesis denote upfield (-) or downfield (+) shifts from the free oligonucleotide under the same conditions.

**Table S6** ^1^H NMR chemical shifts of the (**5a**) (H_2_O : D_2_O, 9 : 1, 298 K, buffer phosphates 100 mM, pH = 7.0) free, and upon the addition to the d(5′-CGCGAATTCGCG-3′)_2_ at r = 0.5, 1, and 2. Shifts are denoting in parenthesis (negative sign upfield and positive sign downfield shifts). A and B denotes the signals of the two moieties of (**5a**). n.o. = not observed.

**Table S7**: Differences in ^1^H chemical shifts of the d(5′-CGCGAATTCGCG-3′)_2_ (buffer phosphate 100 mM, pH = 7.0) upon the addition of complex (**5a**) in various [Ru]/nucleotide ratios at 298 K, 500 MHz. Values in parenthesis denote upfield (-) or downfield (+) shifts from the free oligonucleotide under the same conditions.

**Table S8** ^1^H NMR chemical shifts of the (**6a**) (H_2_O : D_2_O, 9 : 1, 298 K, buffer phosphates 100 mM, pH = 7.0) free, and upon the addition to the d(5′-CGCGAATTGGCC-3′)_2_ at r = 0.5, 1, and 2. Shifts are denoting in parenthesis (negative sign upfield and positive sign downfield shifts). A and B denotes the signals of the two moieties of (**6a**). n.o. = not observed.

**Table S9**: Differences in ^1^H chemical shifts of the d(5′-CGCGAATTGGCC-3′)_2_ (buffer phosphate 100 mM, pH = 7.0) upon the addition of complex (**6a**) in various [Ru]/nucleotide ratios at 298 K, 500 MHz. Values in parenthesis denote upfield (-) or downfield (+) shifts from the free oligonucleotide under the same conditions.

**Table S10**: ^1^H NMR chemical shifts of the exchangeable imino and amino protons of the free d(5′-CGCGAATTGGCC-3′)_2_ (H_2_O : D_2_O, 9 : 1, 298 K, buffer phosphates 100 mM, pH = 7.0), and induced shifts upon the addition (**4a**) at r = 0.5, 1, and 2. Negative sign for upfield shifts and positive sign for downfield shifts (in parenthesis). In bold indicated shifts which are higher than 0.05 ppm. n.o. = not observed.

**Table S11**: ^1^H NMR chemical shifts of the exchangeable imino and amino protons of the free d(5′-CGCGAATTGGCC-3′)_2_ (H_2_O : D_2_O, 9 : 1, 298 K, buffer phosphates 100 mM, pH = 7.0), and induced shifts upon the addition (**5a**) at r = 0.5, 1, and 2. Negative sign for upfield shifts and positive sign for downfield shifts (in parenthesis). In bold indicated shifts which are higher than 0.05 ppm. n.o. = not observed.

**Table S12**: ^1^H NMR chemical shifts of the exchangeable imino and amino protons of the free d(5′-CGCGAATTGGCC-3′)_2_ (H_2_O : D_2_O, 9 : 1, 298 K, buffer phosphates 100 mM, pH = 7.0), and induced shifts upon the addition (**6a**) at r = 0.5, 1, and 2. Negative sign for upfield shifts and positive sign for downfield shifts (in parenthesis). In bold indicated shifts which are higher than 0.05 ppm. n.o. = not observed.

**Figure S60.** Comparison of chemical shift differences (Δδ, ppm) for the H6/H8 protons of the d(5′-CGCGAATTGGCC-3′)₂ duplex upon titration with complexes (**4**)Cl₃ (blue) and (**7**)Cl₄ (orange) at three different ratios: (left) r = 0.5, (middle) r = 1, and (right) r = 2.

**Figure S61.** Imino protons regions of the ¹H NMR spectra (H₂O : D₂O, 9 : 1, 298 K, phosphate buffer 100 mM, pH = 7.0) with proton assignments, recorded during the titration of d(5′-CGCGAATTGGCC-3′)₂ with: (A) complex (**4**)Cl₃ and (B) complex (**7**)Cl₄.

**Figure S62.** Comparison of chemical shift differences (Δδ, ppm) for the H6/H8 protons of the d(5′-CGCGAATTCGCG-3′)₂ duplex upon titration with complexes (**5**)Cl₃ (blue) and (**8**)Cl₄ (orange) at three different ratios: (left) r = 0.5, (middle) r = 1, and (right) r = 2.

**Figure S63.** Comparison of chemical shift differences (Δδ, ppm) for the H6/H8 protons of the d(5′-CGCGAATTCGCG-3′)₂ duplex upon titration with complexes (**6**)Cl₃ (blue) and (**9**)Cl₄ (orange) at three different ratios: (left) r = 0.5, (middle) r = 1, and (right) r = 2.

**Figure S64.** Fluorescence emission spectra of DNA–EtBr titrated with (**1a**)–(**6a**) at 298 K. [DNA] = 20 μM, [EB] = 5.2 μM, and [complex] = 0 to 30.10 μM. (A) (**1a**), (B) (**2a**), (C) (**3a**), (D) (**4a**), (E) (**5a**) and (F) (**6a**).

**Figure S65.** Stern–Volmer plots for the interaction of complexes with DNA–EB at 298 K. (A) (**1a**), (B) (**2a**), (C) (**3a**), (D) (**4a**), (E) (**5a**) and (F) (**6a**).

**Figure S66.** The double-log plot of complexes quenching effect on d(CGCGAATTCGCG)_2_-EB system fluorescence at 298 K. (A) (**1a**), (B) (**2a**), (C) (**3a**), (D) (**4a**), (E) (**5a**) and (F) (**6a**).

**S1. Experimental**

S1.1 Materials and Μethods

Hydrated ruthenium trichloride, RuCl_3_·3H_2_O was purchased from Precious Chemical Company (Pittsburgh, USA). 1,10-phenanthroline, 4,4′- bipyridine, 1,2-bis(4-pyridyl)ethane, 1,3-bis(4-pyridyl)propane, α-phellandrene were purchased from Sigma-Aldrich. Benzo[h]quinolone was purchased from fluorochem. The deoxynucleotide d(5′-CGCGAATTCGCG-3′) (DNA) was purchased from Eurogentec and purified by standard purification option. DNA concentrations were quantified by measuring the absorbance at 260 nm. The complexes [(η^6^-cym)Ru(μ-Cl)Cl]_2_^110^ , [(η^6^-cym)Ru(phe)Cl]PF_6_^111^ and [(η^6^-cym)Ru(bq)Cl]^112^ were synthesized according to the literature methods. All solvents were of analytical grade and were used without further purification. High resolution electrospray ionization mass spectra (HR-ESI-MS) were were acquired using a Thermo Scientific, LTQ Orbitrap XL™ system (Figs S1-S6). NMR spectra were recorded on Bruker Avance spectrometers operating at 400.13 and 500.13 MHz for ¹H, and processed using Topspin 4.1.1 (Bruker Analytik GmbH). ^1^H NMR spectra of the synthesized complexes were recorded in acetone (Figs S7-S12), while the corresponding chloride salt complexes were measured in H₂O/D₂O (9:1, 100 mM phosphate buffer, pH 7.0) at 298 K (Figs S52-S54). Under identical conditions, DNA spectra were recorded as follows: One-dimensional ¹H NMR spectra were acquired from samples with DNA concentrations of approximately 0.5 mM, whereas two-dimensional NMR experiments were performed with more concentrated samples (∼2 mM). Two-dimensional COSY and NOESY experiments were employed to assist in the assignment of ¹H signals. NOESY experiments were conducted with a mixing time of 300 ms, using data sets acquired with 4096 × 512 complex points and a sweep width of 8 kHz in both dimensions.

S1.2 Crystal structure determination

Suitable crystals of compounds {[Ru(cym)(bq)]_2_(*μ*-BL-1)}(PF_6_)_2_, (**1**), {[Ru(cym)(bq)]_2_(*μ*-BL-2)}(PF_6_)_2_, (**2**) and {[Ru(cym)(bq)]_2_(*μ*-BL-3)}(PF_6_)_2_, (**3**) were glued to a thin glass fiber with cyanoacrylate adhesive and placed on the goniometer head. Diffraction data were collected on a Bruker D8 Quest Eco diffractometer, equipped with a Photon II detector and a TRIUMPH (curved graphite) monochromator utilizing Mo Ka radiation (λ = 0.71073 Å) using the APEX 3 software package^113^. The collected frames were integrated with the Bruker SAINT software using a wide-frame algorithm. Data were corrected for absorption effects using the Multi-Scan method (SADABS)^114^. The structures were solved using the Bruker SHELXT Software Package and refined by full-matrix least squares techniques on F2 (SHELXL 2018/3)^115^ via the ShelXle interface^116^. The non-H atoms were treated anisotropically, whereas the organic H atoms were placed in calculated, ideal positions and refined as riding on their respective carbon atoms. In all cases the PF_6_^-^ counter anions were refined as disordered in two positions with occupancies from approximately 51 to 79 %. Some cross-peaks in 2D NMR spectra prompt us to refine the phenylquinoline moieties as disordered in two positions with alternating carbon and nitrogen donor atoms. Only in the case of **1** the refinement led to improved R factors with occupancies 63.9 and 36.1 %. In the crystal structure of (**3**) there is void space (approximately 24 % of the unit cell volume) filled with electron density that belongs to solvated molecules and could not be modelled. It was treated with the SQUEEZE routine incorporated in PLATON^117^. SQUEEZE suggested 557 electrons in the solvent accessible void space which correspond to 9 – 10 solvated chloroform molecules or solvent mixtures per unit cell. PLATON was used for geometric calculations, and X-Seed^118^ for molecular graphics. Details on data collection and refinement are presented in Table S1. Full details on the structures can be found in the CIF files. **CCDC 2480256-2480258** contain the supplementary crystallographic data for this paper. These data can be obtained free of charge via www.ccdc.cam.ac.uk/data_request/cif.

**Table S1.** Crystal data and structure refinement for the prepared compounds.

| Compound | (**1**) | (**2**) | (**3**)⋅solvents |
| --- | --- | --- | --- |
| Empirical formula | C_56_H_52_F_12_N_4_P_2_Ru_2_ | C_58_H_56_F_12_N_4_P_2_Ru_2_ | C_59_H_58_F_12_N_4_P_2_Ru_2_ |
| Formula | {[Ru(cym)(pq)]_2_(bpy)}(PF_6_)_2_ | {[Ru(cym)(pq)]_2_(ebp)} (PF_6_)_2_ | {[Ru(cym)(pq)]_2_(pbp)} (PF_6_)_2_ |
| Formula weight | 1273.09 | 1301.14 | 1315.17 |
| Temperature (K) | 296(2) | | |
| Wavelength (Å) | 0.71073 | | |
| Crystal system | Monoclinic | Monoclinic | Orthorhombic |
| Space group | P2_1_/*c* | *P2_1_/n* | *Pbcn* |
| Unit cell dimensions  *a*, *b*, *c* (Å)  *α*, *β*, *γ* (^o^) | 15.0334(18), 12.2099(13), 14.9357(16)  90, 99.617(4), 90 | 12.0285(18), 14.1760(19), 16.545(3)  90, 97.378(7), 90 | 12.0886(6), 15.2050(8), 37.266(2)  90, 90, 90 |
| Volume (Å^3^) | 2703.0(5) | 2797.8(8) | 6849.8(6) |
| Z | 2 | 2 | 4 |
| Density (g/cm^3^) (calculated) | 1.564 | 1.544 | 1.275 |
| Absorption coefficient (mm^-1^) | 0.701 | 0.679 | 0.555 |
| F(000) | 1284 | 1316 | 2664 |
| Crystal size (mm^3^) | 0.30 x 0.28 x 0.06 | 0.35 x 0.25 x 0.05 | 0.20 x 0.18 x 0.17 |
| θ range for data collection (^o^) | 2.439 to 24.995 | 2.869 to 24.997 | 2.414 to 25.000 |
| Index ranges | -17 ≤ *h* ≤ 17, -14 ≤ *k* ≤ 14, -17 ≤ *l* ≤ 17 | -14 ≤ *h* ≤ 14, -16 ≤ *k* ≤ 16, -19 ≤ *l* ≤ 19 | -14 ≤ *h* ≤ 14, -18 ≤ *k* ≤ 18, -44 ≤ *l* ≤ 44 |
| Reflections collected | 34804 | 32595 | 102068 |
| Independent reflections [R_int_] | 4733 [0.1164] | 4923 [0.1140] | 6020 [0.2062] |
| Completeness to *θ* | 99.8% | 99.8% | 99.9% |
| Refinement method | Full-matrix least-squares on F^2^ | | |
| Data / restraints / parameters | 4733 / 472 / 453 | 4923 / 210 / 419 | 6020 / 327 / 412 |
| Goodness-of-fit | 1.189 | 1.048 | 0.949 |
| Final R indices [I > 2σ(I)]  (R_obs,_ wR_obs_) | 0.0908, 0.1820 | 0.0656, 0.1518 | 0.0674, 0.1630 |
| R indices [all data]  (R_all_, wR_all_) | 0.1260, 0.1935 | 0.1155, 0.1712 | 0.1473, 0.1867 |
| Largest diff. peak and hole (e·Å^-3^) | 1.093 and -0.829 | 1.069 and -0.972 | 0.392 and -0.526 |

R = Σ||F_o_|-|F_c_|| / Σ|F_o_|, wR = {Σ[w(|F_o_|^2^ - |F_c_|^2^)^2^] / Σ[w(|F_o_|^4^)]}^1/2^ and w=1/[σ^2^(Fo^2^)+(0.0335P)^2^+23.0550P]

where P=(Fo^2^+2Fc^2^)/3. **1**(PF_6_)_2_ a = 0.0335, b = 23.0550; **2**(PF_6_)_2_ a = 0.0927, b = 0.2116; **3**(PF_6_)_2_⋅solvents

a = 0.0965, b = 0

S1.3 Interactions of (**4a**), (**5a**) and (**6a**) with the oligonucleotide d(5′-CGCGAATTCGCG-3′)_2_.

1D ^1^H NMR spectra were recorded at three different ratios of [complex]:[oligonucleotide duplex], r = 0.5, 1 and 2. Signal assignments were assisted by 2D COSY and NOESY experiments (Figs S25 – S51). Additionally, 2D NOESY spectra were recorded to determine inter-proton contacts within the DNA duplexes, and between the protons of the complexes (**4a**) – (**6a**). Weak NOE inter-proton interactions between the DNA duplexes and (**4a**) – (**5a**) were detected; however, these could not be assigned unambiguously. The results are summarized in Tables S4 – S12.

S1.4 ^1^H NMR assignments of d(5′-CGCGAATTCGCG-3′)_2_.

Assignments of exchangeable and non-exchangeable protons were performed according to the protocol described previously^119^. For the hexameric oligonucleotide, it is known that the signals of the H6 and H5 protons of cytosines are double and the chemical shifts οf these protons are in the aromatic region and in the H1' region of the sugars respectively. The C1H6 resonance assigned as the lowest field peak of the three H6 peaks of cytosines. Additionally, this proton, as a 5'-terminal residue proton, will have only one base-to-H1’ crosspeak for a given base proton, in the NOESY spectrum. Then, following the path C(n)/G(n)H1' → G(n+1)H8/C(n+1)H6, G(n+1)H8/C(n+1)H6 → G (n+1)/C(n+1)H1' etc., in the NOE map, all aromatic protons (H6 and H8) and H1' of the sugars were assigned. From the COSY crosspeaks with the corresponding H6 peaks can easily identify the H5 double peaks of cytosines.

The ^1^H NMR spectrum at the imino region exhibits two signals at 13.11 ppm and 13.14 ppm assigned to G2H1 and G4H1 correspondingly, indicating that either the terminal C1-G6 base pairs of the duplex do not form a Watson-Crick (W-C) hydrogen bond, or the G6-H1 is rapidly exchanged with the solvent. Following the NOE connectivities for the exchangeable oligonucleotide protons, the proton signals of the exocyclic amino groups of the bases G2, G4 and G6 were assigned (Fig. 4). The proton signals of the C1, C3 and C5 amino groups, are matching with the signals of the complementary guanine bases. The correlation with the non-exchangeable oligonucleotide protons was achieved through intra-strand NOE connectivities G(n)H2(n.b.) → C(n-1)H1′ and inter-strand NOE connectivities GH2(n.b.) → C(n-2)H5. The non-exchangeable protons were assigned either from COSY experiments (C(n)H5 → C(n)H6, C(n)/G(n)H1′ → C(n)/G(n)H2′ and Η2″, C(n)/G(n)H2′ → C(n)/G(n)H3′ etc.) or NOESY (G(n)/C(n)H8/H6 → G(n + 1)/C(n + 1)H1′, G(n + 1)/ C(n + 1)H1′ → G(n + 1)/C(n + 1)H8/H6, etc.).

S1.5 Fluorescence measurements

Fluorescence emission study was carried out using a Jasco FP-8300 fluorimeter equipped with xenon lamp source. All the experiments were done by using a 10 mm path length cuvette in a 100 mM phosphate buffer at pH 7.0. Successive amounts of each complex (75 – 750 μL) from a stock solution of 1 mM were added to a 20 μM of d(5′-CGCGATCGCG-3′)_2_ saturated with ethidium bromide EtBr (5.2 μM)^120^, which is an important DNA probe for competitive studies with other DNA binders^121^. A DNA–EB sample was titrated with (**1a**) – (**6a**) and the emission spectra were recorded at wavelength of 510 – 850 nm with excitation at 480 nm in a 1 cm quartz cell. The excitation and emission slit widths were kept at 5 nm. All the measurements were recorded after 15 min of incubation at 298 K. The calculations of Ksv and Kb were performed as described below.

Fluorescence quenching property described by the Stern-Volmer equation^122^.

F_0_/F = 1 + K_sv_[Q]

where F_0_ and F are the fluorescence intensities in the absence and the presence of quencher, respectively. K_sv_ is the Stern-Volmer quenching constant and [Q] is the concentration of complex. The slope of the linear plot of F_0_/F versus [Q] shows the value of Ksv.

The binding constant (K_b_) between the complexes and d(CGCGAATTCGCG)_2_ determined using the following double logarithmic equation^123^.

log[(F_0_-F)/F] = nlog[Q] + logKb

The plot log[F_0_-F/F] versus log[Q] is a straight line and the values of n and Kb can be found from slope and the intercept of the plot respectively.

S1.6 Cell culture

The human breast adenocarcinoma cell line MCF-7 and the mouse embryonic fibroblast cell line NIH-3T3 were cultured in high- glucose Dulbecco’s modified Eagle’s medium (DMEM) supplemented with 10% fetal bovine serum and 1% penicillin/ streptomycin. The human ovarian cancer cell line A2780 and its cisplatin resistant derivative (A2780cis-res) were maintained in RPMI-1640 medium supplemented with 10% fetal bovine serum and 1% penicillin/streptomycin. All cell lines were routinely passaged every 2 – 3 days, and incubated at 37 °C in a humidified atmosphere containing 5% CO_2_.

S1.7 Cell growth assay

Cell growth and the cytotoxic effects of the complexes were monitored using the IncuCyte Zoom system and software(Essen BioScience, Hertfordshire, United Kingdom), as previously described^84^. The IC_50_ values of cisplatin and of the complexes were determined by fitting a log(concentration) versus normalized response curve using GraphPad Prism, version 8.01.

S1.8 Synthesis of the complexes

The complexes (**1**) – (**3**) were synthesized as follows:

In a 20 mL round-bottom flask, 0.06 mmol of [(*η^6^*-cym)Ru(bq)Cl] was dissolved in 8 mL of a mixture of H_2_O:Me_2_CO (7:1) and 0.055 mmol of AgNO_3_ was then added. The yellow solution was stirred at room temperature for 32 h in the dark and the precipitated AgCl was removed by centrifugation. 0.03 mmol of BL-i (i = 1, 2, 3) was added, and the mixture was stirred at room temperature for 16 hours. Subsequently, 0.3 mmol of KPF_6_ was added to the clear green solution and the mixture was cooled in the refrigerator, resulting in a microcrystalline dark green solid. This solid was collected by filtration, washed with H_2_O (3 × 3 mL) and dried under vacuum over P_2_O_5_.

The complexes (**4**) – (**6**) were synthesized in two steps:

In the first step, 0.04 mmol of [(η⁶-cym)Ru(phen)Cl]PF₆ was dissolved in 8 mL of a 7:1 H₂O:Me₂CO mixture in a 20 mL round-bottom flask, followed by the addition of 0.035 mmol of AgNO₃. The solution was stirred at 75 ^ο^C for 3 h in the dark and the precipitated AgCl was removed by centrifugation. To this solution, 0.06 mmol of BL-i (i = 1, 2, 3) was added and the mixture was stirred at 75 ^ο^C for 16 hours. After cooling, 0.1 mmol of KPF_6_ was added to the solution and the monometallic complex [(*η^6^*-cym)Ru(phen)(BL-i)](PF_6_)_2_ was formed, which was collected by filtration, washed with H_2_O (3 × 3 mL) and dried under vacuum over P_2_O_5_.

In a separate 20 mL round-bottom flask containing 8 mL of a mixture of H_2_O:Me_2_CO (10:1), 0.04 mmol [(*η^6^*-cym)Ru(bq)Cl] and 0.035 mmol of AgNO_3_ were added. The mixture was stirred at room temperature for 36 h in the dark and the precipitated AgCl was removed by centrifugation. The resulting solution and the 2 ml Me_2_CO solution of the monometallic complex [(*η^6^*-cym)Ru(phen)(BL-i)](PF_6_)_2_ were mixed and stirred at room temperature for 16 h. After that, 10 mL hydrated solution of 1 mmol KPF_6_ was added and the desired green bimetallic complex was formed. The complex was isolated, washed with H_2_O (3 × 5 mL) and dried under vacuum over P_2_O_5_.

**{[(η^6^-cym)Ru(bq)]_2_(μ-BL-1)}(PF_6_)_2_,** (**1**). Yield 74%. **^1^H NMR** (400 MHz, 298 K, acetone-d_6_, *δ* in ppm): H_2c_: 6.31 (d, 2H), H_3c_: 5.62 (d, 2H), H_5c_: 6.36 (d, 2H), H_6c_: 6.06 (d, 2H), H_7c_: 1.69 (s, 6H), H_8c_: 2.19 (m, 2H), H_9c_: 0.71 (d, 6H), H_10c_: 0.63 (d, 6H), H_a_: 8.70 (d, 4H), H_b_: 7.41 (d, 4H), H_2_: 10.04 (d, 2H), H_3_: 7.80 (dd, 2H), H_4_: 8.54 (d, 2H), H_5_: 7.71 (s, 2H), H_6_: 7.70 (s, 2H), H_7_: 7.84 (d, 2H), H_8_: 7.71 (dd, 2H), H_9_: 8.74 (d, 2H). **HR-ESI-MS** *m/z* = 1129.2076, calc. 1129.1915 for [C_56_H_54_F_6_N_4_P^101^Ru_2_]^+^, {[[(η^6^-cym)Ru(bq)]_2_(μ-BL-1)](PF_6_)}^+^.

**{[(*η^6^*-cym)Ru(bq)]_2_(μ-BL-2)}(PF_6_)_2_,** (**2**). Yield 71%. **^1^H NMR** (400 MHz, 298 K, acetone-d_6_, *δ* in ppm): H_2c_: 6.29 (d, 2H), H_3c_: 5.57 (d, 2H), H_5c_: 6.31 (d, 2H), H_6c_: 6.05 (d, 2H), H_7c_: 1.60 (s, 6H), H_8c_: 2.18 (m, 2H), H_9c_: 0.71 (d, 6H), H_10c_: 0.63 (d, 6H), H_a_: 8.42 (d, 4H), H_b_: 6.96 (d, 4H), H_c_: 2.66 (s, 4H), H_2_: 10.01 (d, 2H), H_3_: 7.81 (dd, 2H), H_4_: 8.55 (d, 2H), H_5_: 7.72 (s, 2H), H_6_: 7.70 (s, 2H), H_7_: 7.87 (d, 2H), H_8_: 7.71 (dd, 2H), H_9_: 8.72 (d, 2H). **HR-ESI-MS**; *m/z* = 1157.2358, calc. 1157.2228 for [C_58_H_58_F_6_N_4_P^101^Ru_2_]^+^, {[[(η^6^-cym)Ru(bq)]_2_(μ-BL-2)](PF_6_)}^+^.

**{[(η^6^-cym)Ru(bq)]_2_(μ-BL-3)}(PF_6_)_2_,** (**3**). Yield 73%. **^1^H NMR** (400 MHz, 298 K, acetone-d_6_, *δ* in ppm): H_2c_: 6.29 (d, 2H), H_3c_: 5.61 (d, 2H), H_5c_: 6.34 (d, 2H), H_6c_: 6.04 (d, 2H), H_7c_: 1.68 (s, 6H), H_8c_: 2.20 (m, 2H), H_9c_: 0.72 (d, 6H), H_10c_: 0.64 (d, 6H), H_a_: 8.46 (d, 4H), H_b_: 6.96 (d, 4H), H_c_: 2.39 (s, 4H), H_d_: 1.58 (s, 4H), H_2_: 10.05 (d, 2H), H_3_: 7.81 (dd, 2H), H_4_: 8.54 (d, 2H), H_5_: 7.71 (s, 2H), H_6_: 7.71 (s, 2H), H_7_: 7.86 (d, 2H), H_8_: 7.72 (dd, 2H), H_9_: 8.73 (d, 2H). **HR-ESI-MS**; *m/z* = 1171.2424, calc. 1171.2385 for [C_59_H_60_F_6_N_4_P^101^Ru_2_]^+^, {[[(η^6^-cym)Ru(bq)]_2_(μ-BL-3)](PF_6_)}^+^.

**[(η^6^-cym)Ru(phen)(μ-BL-1)(η^6^-cym)Ru(bzq)](PF_6_)_3_,** (**4**). Yield 77%. **^1^H NMR** (400 MHz, 298 K, acetone-d_6_, *δ* in ppm): H_2c_: 6.32 (d, 1H), H_3c_: 5.62 (d, 1H), H_5c_: 6.36 (d, 1H), H_6c_: 6.07 (d, 1H), H_7c_: 1.69 (s, 3H), H_8c_: 2.22 (m, 1H), H_9c_: 0.71 (d, 3H), H_10c_: 0.63 (d, 3H), H_2’c/6’c_: 6.85 (d, 2H), H_3’c/5’c_: 6.48 (d, 2H), H_7’c_: 1.93 (s, 3H), H_8’c_: 2.58 (m, 1H), H_9’c/10’c_: 0.83 (d, 6H), H_a_: 8.72 (d, 2H), H_b_: 7.43 (d, 2H), H_a’_: 8.79 (d, 2H), H_b’_: 7.62 (d, 2H), H_2_: 10.03 (d, 1H), H_3_: 7.81 (dd, 1H), H_4_: 8.55 (d, 1H), H_5_: 7.72 (s, 1H), H_6_: 7.70 (s, 1H), H_7_: 7.85 (d, 1H), H_8_: 7.70 (dd, 1H), H_9_: 8.74 (d, 1H), H_2’/9’_: 10.43 (d, 2H), H_3’/8’_: 8.37 (dd, 2H), H_4’/7’_: 9.05 (d, 2H), H_5’/6’_: 8.30 (s, 2H). **HR-ESI-MS**; *m/z* = 1276.1766, calc. 1276.1588 for [C_55_H_53_F_12_N_5_P_2_^101^Ru_2_]^+^, [(η^6^-cym)Ru(phen)(μ-BL-1)(η^6^-cym)Ru(bq)](PF_6_)_2_}^+^.

**[(η^6^-cym)Ru(phen)(μ-BL-2)(η^6^-cym)Ru(bzq)](PF_6_)_3_,** (**5**). **^1^H NMR** (400 MHz, 298 K, acetone-d_6_, *δ* in ppm): H_2c_: 6.29 (d, 1H), H_3c_: 5.59 (d, 1H), H_5c_: 6.34 (d, 1H), H_6c_: 6.04 (d, 1H), H_7c_: 1.67 (s, 3H), H_8c_: 2.19 (m, 1H), H_9c_: 0.70 (d, 3H), H_10c_: 0.63 (d, 3H), H_2’c/6’c_: 6.83 (d, 2H), H_3’c/5’c_: 6.43 (d, 2H), H_7’c_: 1.84 (s, 3H), H_8’c_: 2.58 (m, 1H), H_9’c/10’c_: 0.83 (d, 6H), H_a_: 8.47 (d, 2H), H_b_: 7.02 (d, 2H), H_c_: 2.69 (t, 2H), H_a’_: 8.53 (d, 2H), H_b’_: 7.24 (d, 2H), H_c’_: 2.71 (t, 2H), H_2_: 10.03 (d, 1H), H_3_: 7.81 (dd, 1H), H_4_: 8.55 (d, 1H), H_5_: 7.73 (s, 1H), H_6_: 7.71 (s, 1H), H_7_: 7.87 (d, 1H), H_8_: 7.72 (dd, 1H), H_9_: 8.72 (d, 1H), H_2’/9’_: 10.40 (d, 2H), H_3’/8’_: 8.38 (dd, 2H), H_4’/7’_: 9.07 (d, 2H), H_5’/6’_: 8.33 (s, 2H). **HR-ESI-MS**; *m/z* = 1304.2021, calc. 1304.1901 for [C_57_H_57_F_12_N_5_P_2_^101^Ru_2_]^+^, [(η^6^-cym)Ru(phen)(μ-BL-2)(η^6^-cym)Ru(bq)](PF_6_)_2_}^+^.

**[(η^6^-cym)Ru(phen)(μ-BL-3)(η^6^-cym)Ru(bzq)](PF_6_)_3_,** (**6**). Yield 74%. **^1^H NMR** (400 MHz, 298 K, acetone-d_6_, *δ* in ppm): H_2c_: 6.29 (d, 1H), H_3c_: 5.59 (d, 1H), H_5c_: 6.33 (d, 1H), H_6c_: 6.04 (d, 1H), H_7c_: 1.68 (s, 3H), H_8c_: 2.21 (m, 1H), H_9c_: 0.71 (d, 3H), H_10c_: 0.64 (d, 3H), H_2’c/6’c_: 6.83 (d, 2H), H_3’c/5’c_: 6.44 (d, 2H), H_7’c_: 1.92 (s, 3H), H_8’c_: 2.58 (m, 1H), H_9’c/10’c_: 0.84 (d, 6H), H_a_: 8.45 (d, 2H), H_b_: 6.96 (d, 2H), H_c_: 2.41 (t, 2H), H_d_: 1.59 (mt, 2H), H_a’_: 8.52 (d, 2H), H_b’_: 7.18 (d, 2H), H_c’_: 2.48 (t, 2H), H_d’_: 1.59 (mt, 2H), H_2_: 10.03 (d, 1H), H_3_: 7.81 (dd, 1H), H_4_: 8.55 (d, 1H), H_5_: 7.73 (s, 1H), H_6_: 7.71 (s, 1H), H_7_: 7.87 (d, 1H), H_8_: 7.72 (dd, 1H), H_9_: 8.72 (d, 1H), H_2’/9’_: 10.40 (d, 2H), H_3’/8’_: 8.38 (dd, 2H), H_4’/7’_: 9.07 (d, 2H), H_5’/6’_: 8.33 (s, 2H). **HR-ESI-MS**; *m/z* = 1318.2067, calc. 1318.2057 for [C_58_H_59_F_12_N_5_P_2_^101^Ru_2_]^+^, [(η^6^-cym)Ru(phen)(μ-BL-3)(η^6^-cym)Ru(bq)](PF_6_)_2_}^+^.

**
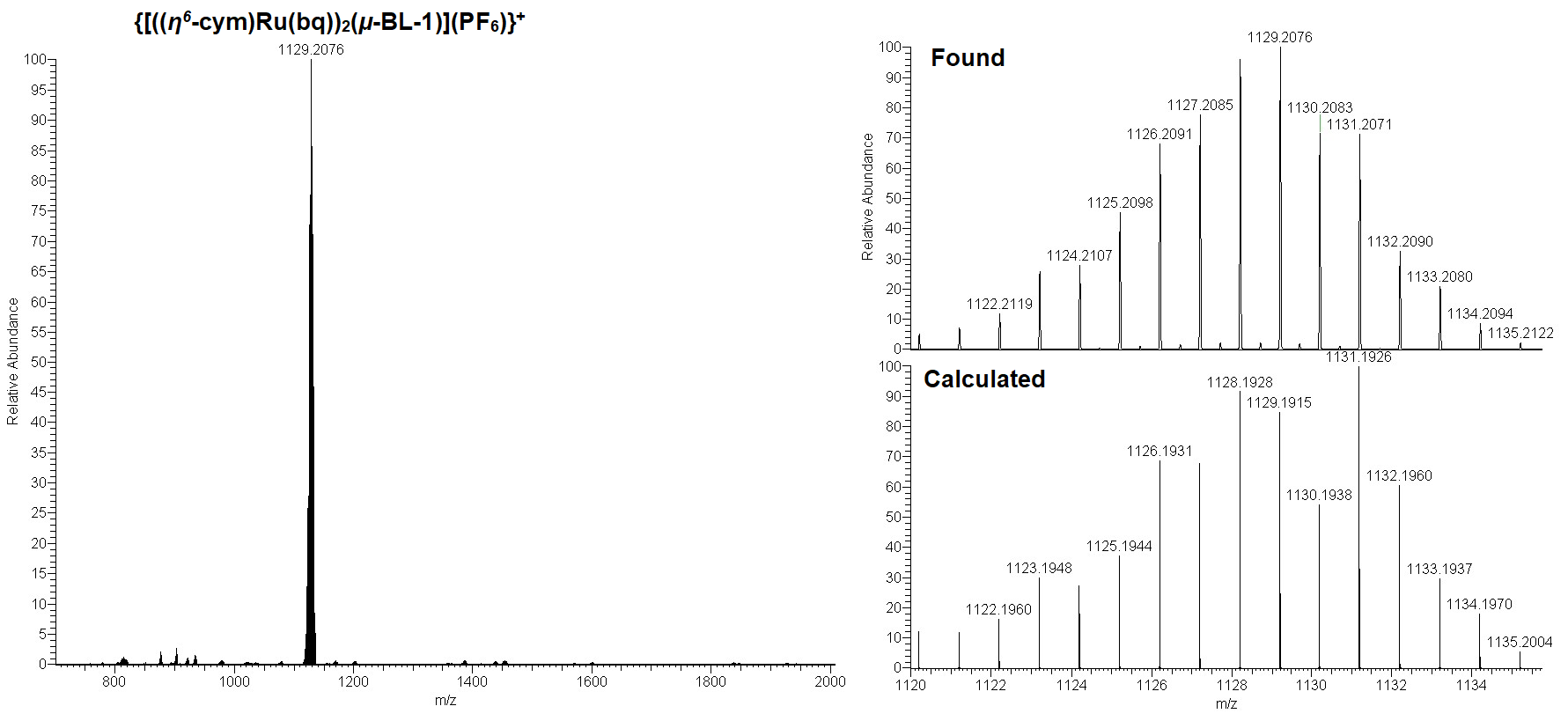
**

**Figure S1**: Part of HR-ESI-MS of the complex (**1**) in acetonitrile at 298 K.


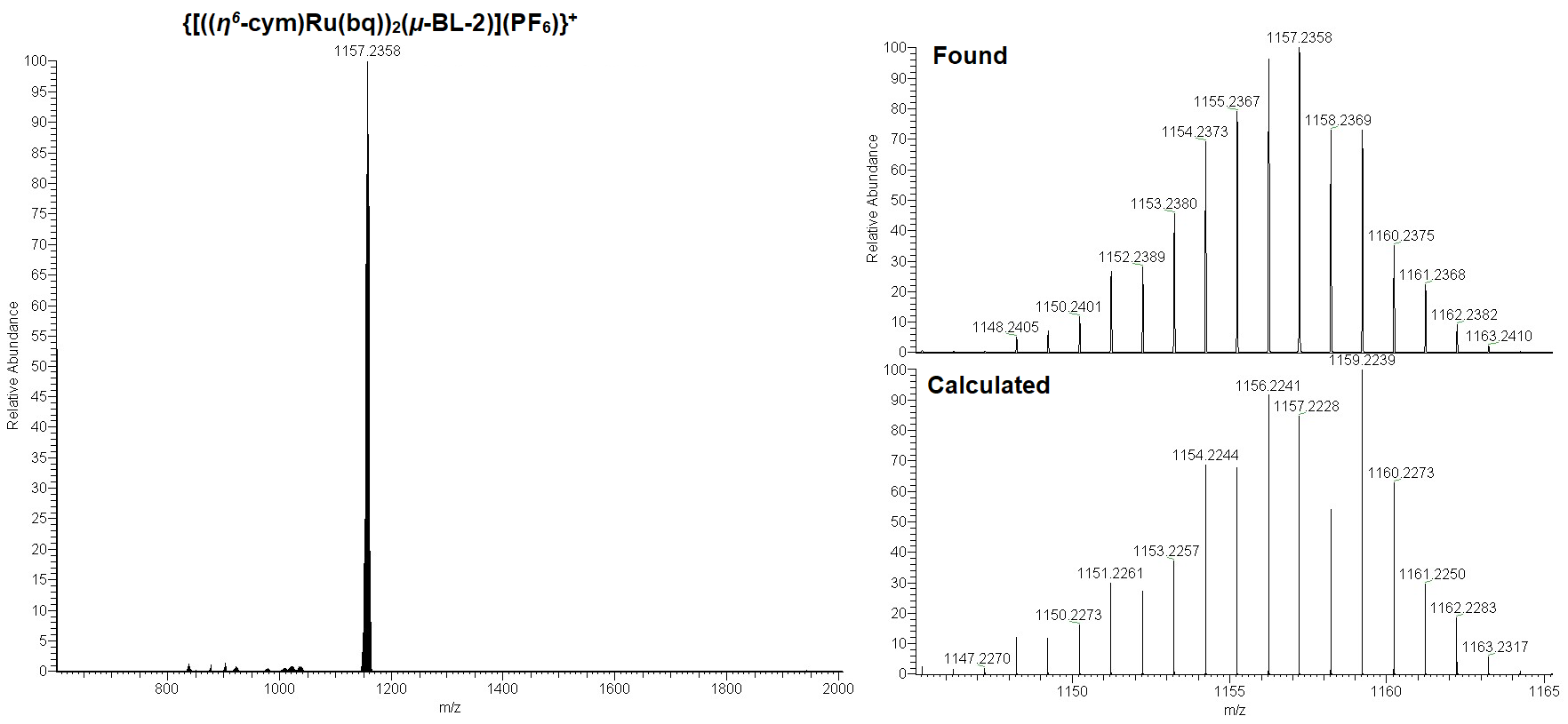


**Figure S2**: Part of HR-ESI-MS of the complex (**2**) in acetonitrile at 298 K.

**
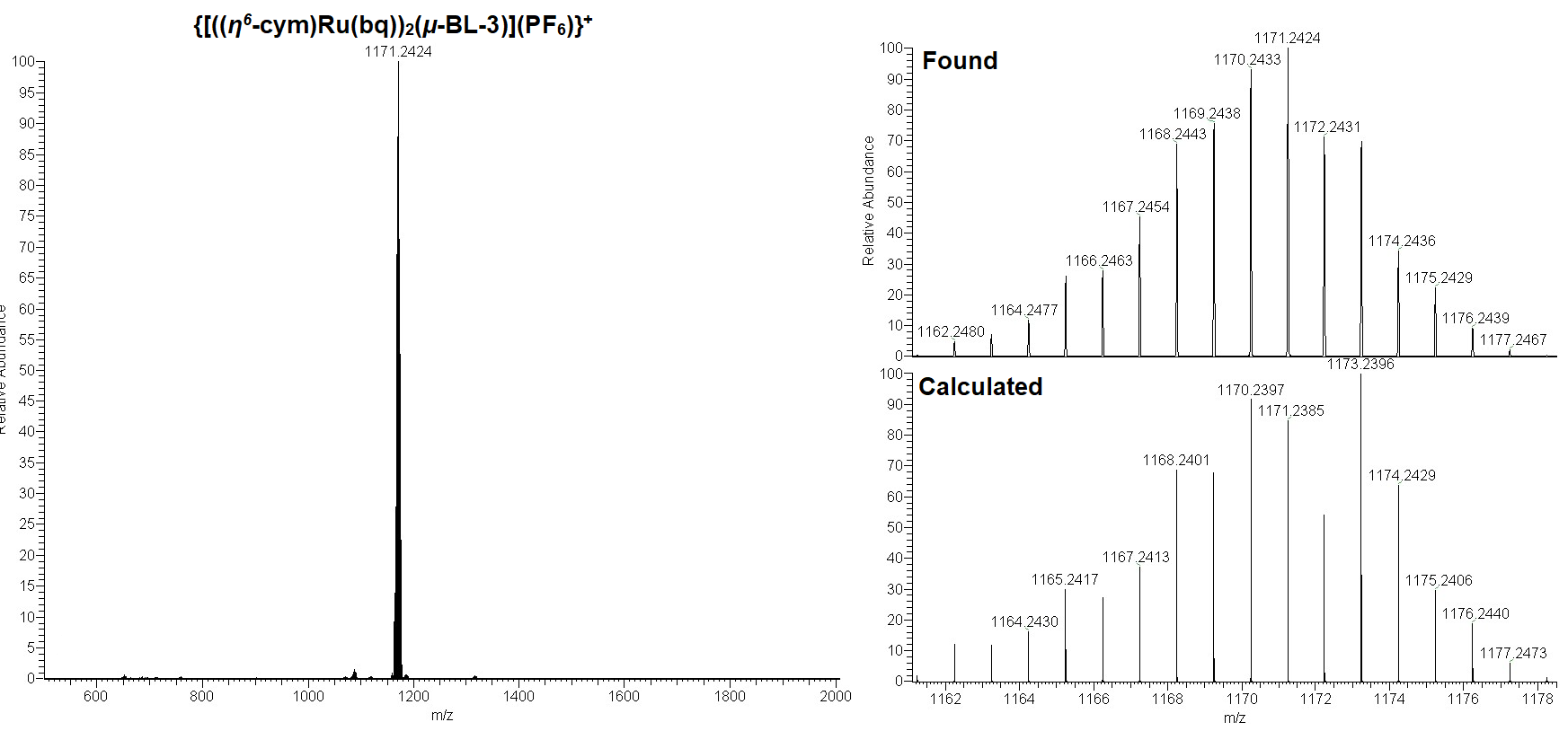
**

**Figure S3**: Part of HR-ESI-MS of the complex (**3**) in acetonitrile at 298 K.


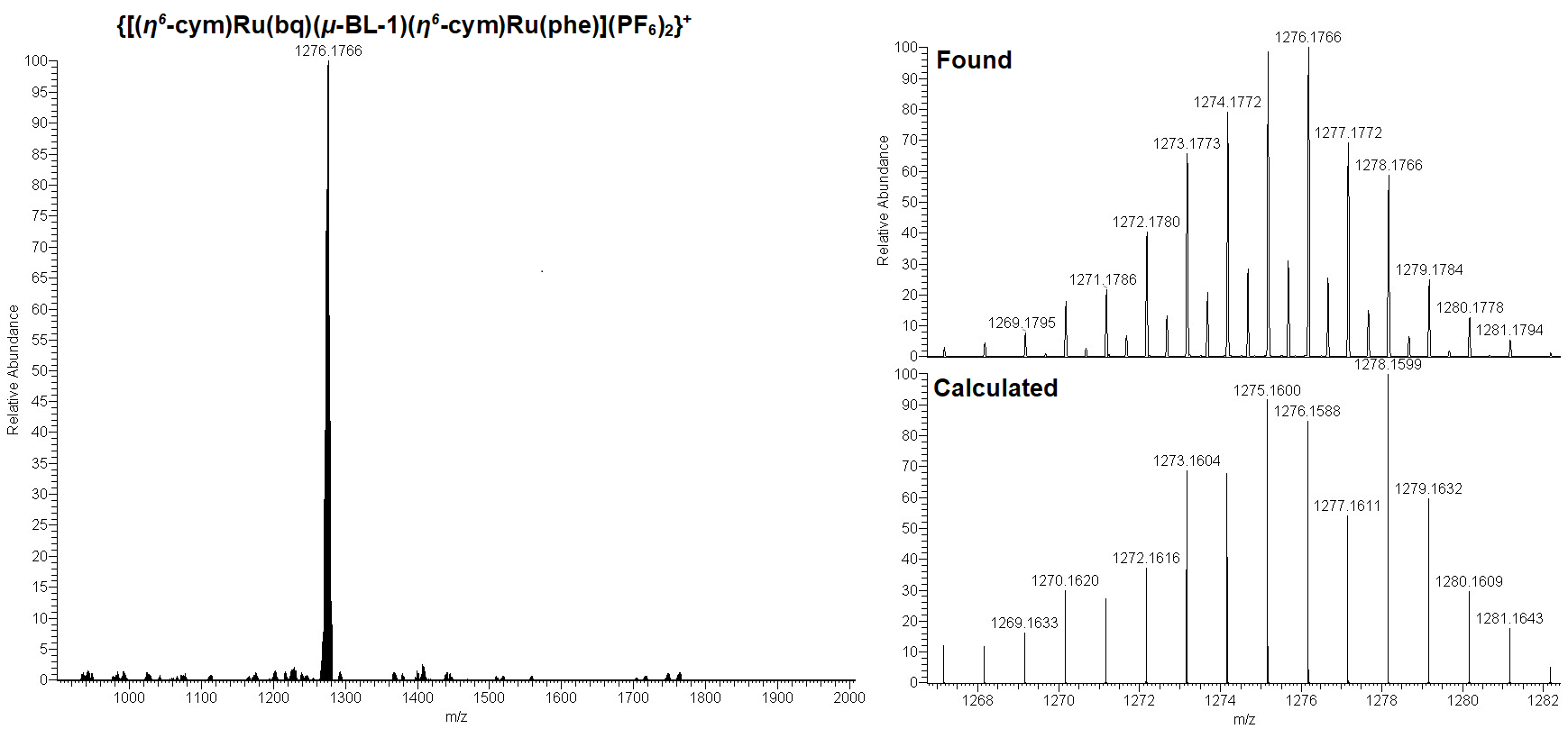


**Figure S4**: Part of HR-ESI-MS of the complex (**4**) in acetonitrile at 298 K.

**
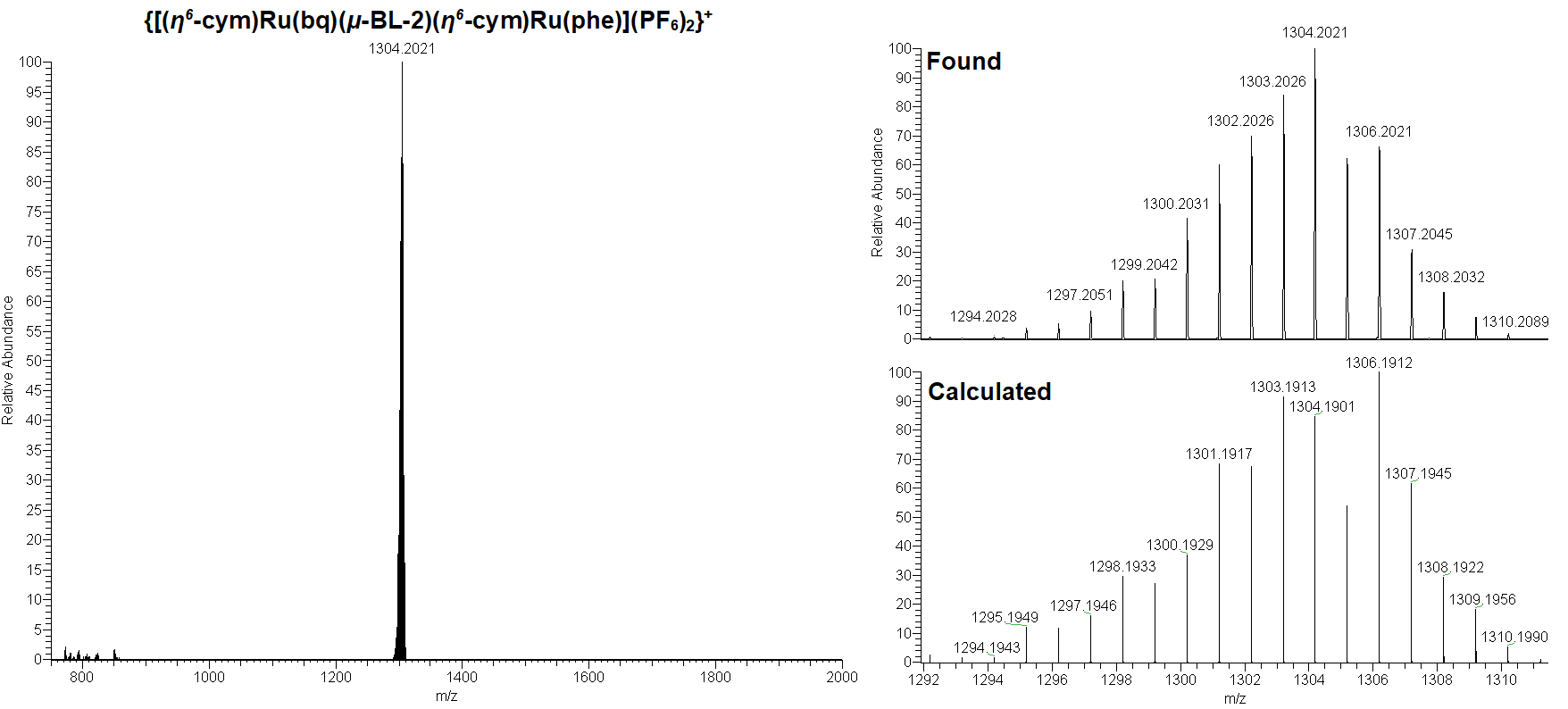
**

**Figure S5**: Part of HR-ESI-MS of the complex (**5**) in acetonitrile at 298 K.

**
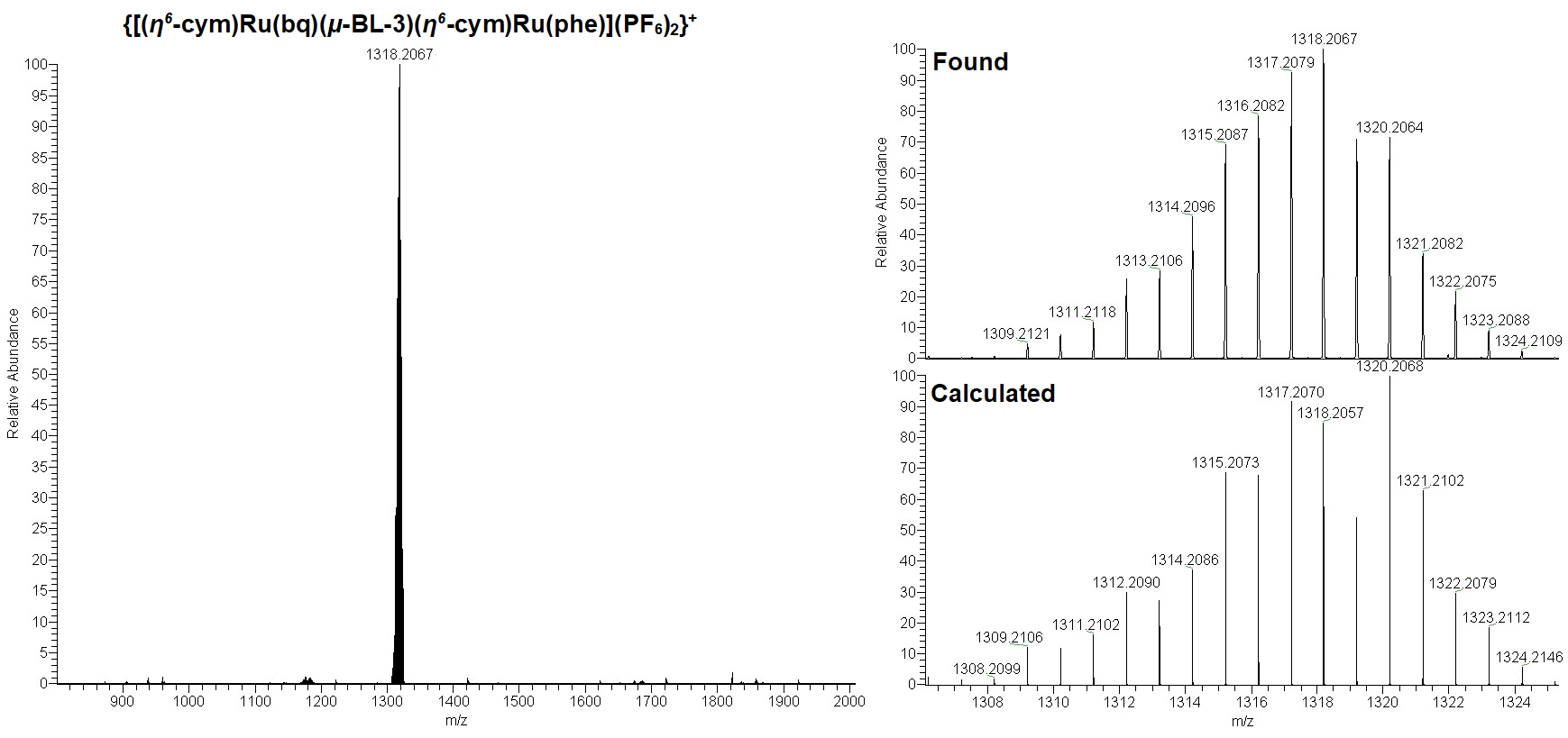
**

**Figure S6**: Part of HR-ESI-MS of the complex (**6**) in acetone at 298 K.

**
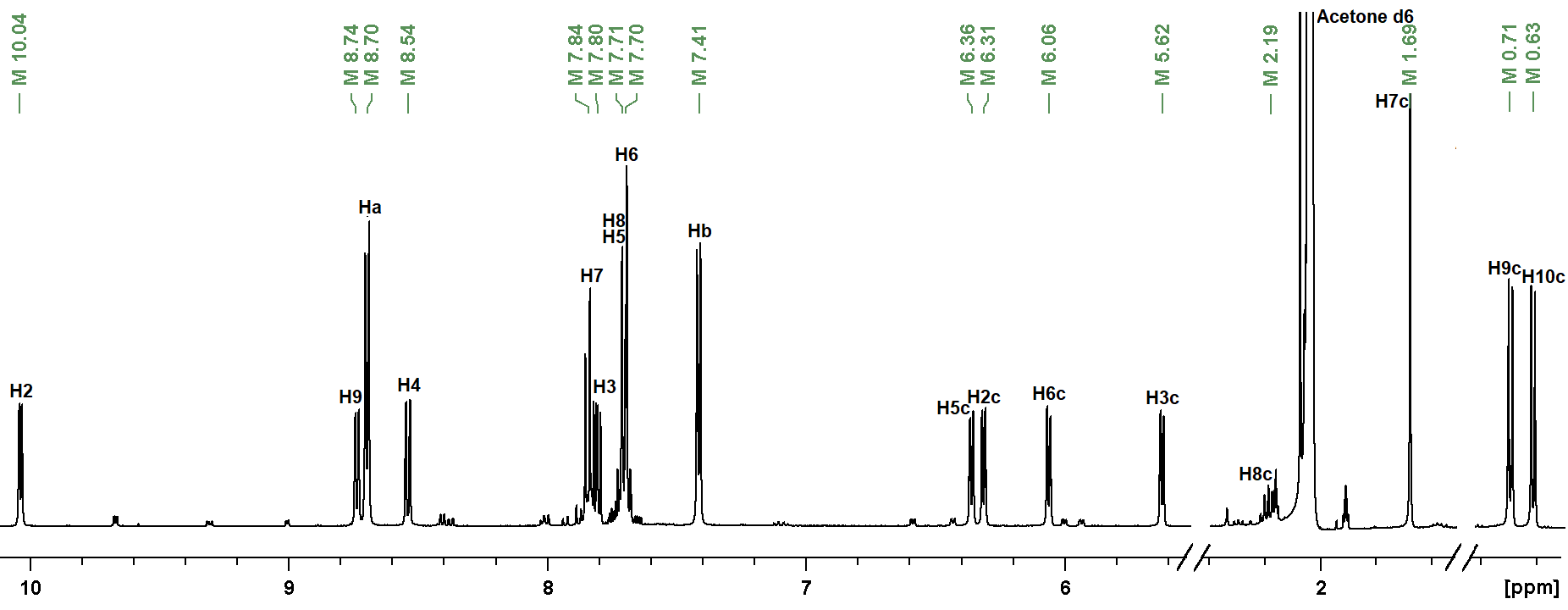
**

**Figure S7**: ^1^H NMR spectrum of the complex (**1**) in acetone-d_6_ at 298 K.

**
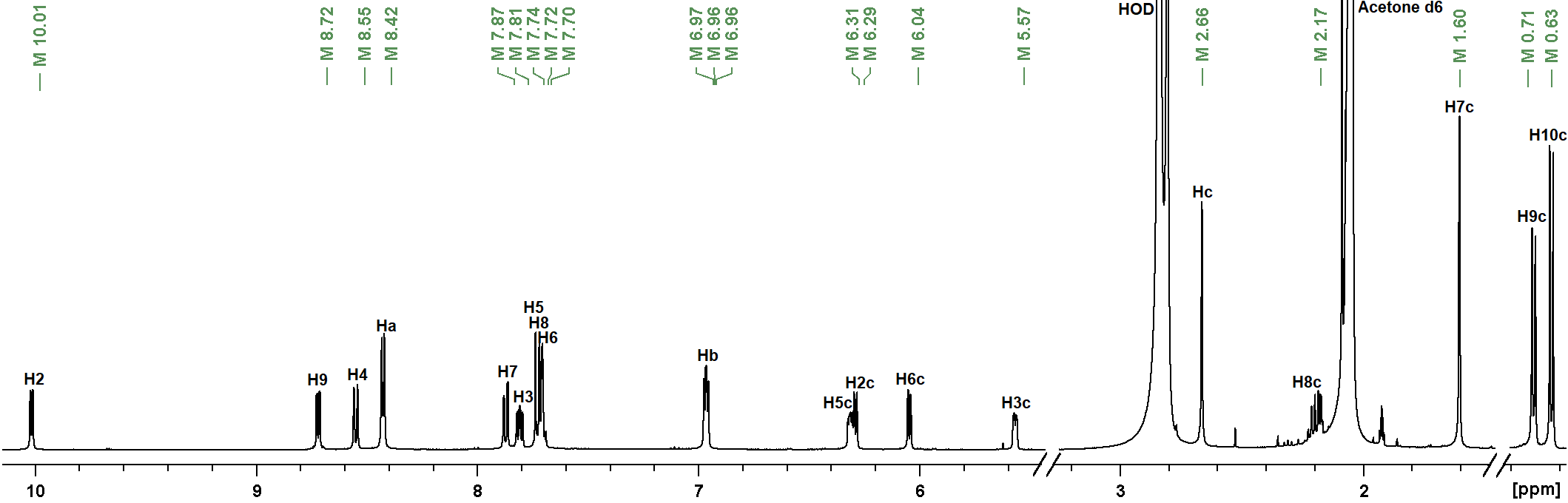
**

**Figure S8**: ^1^H NMR spectrum of the complex (**2**) in acetone-d_6_ at 298 K.

**
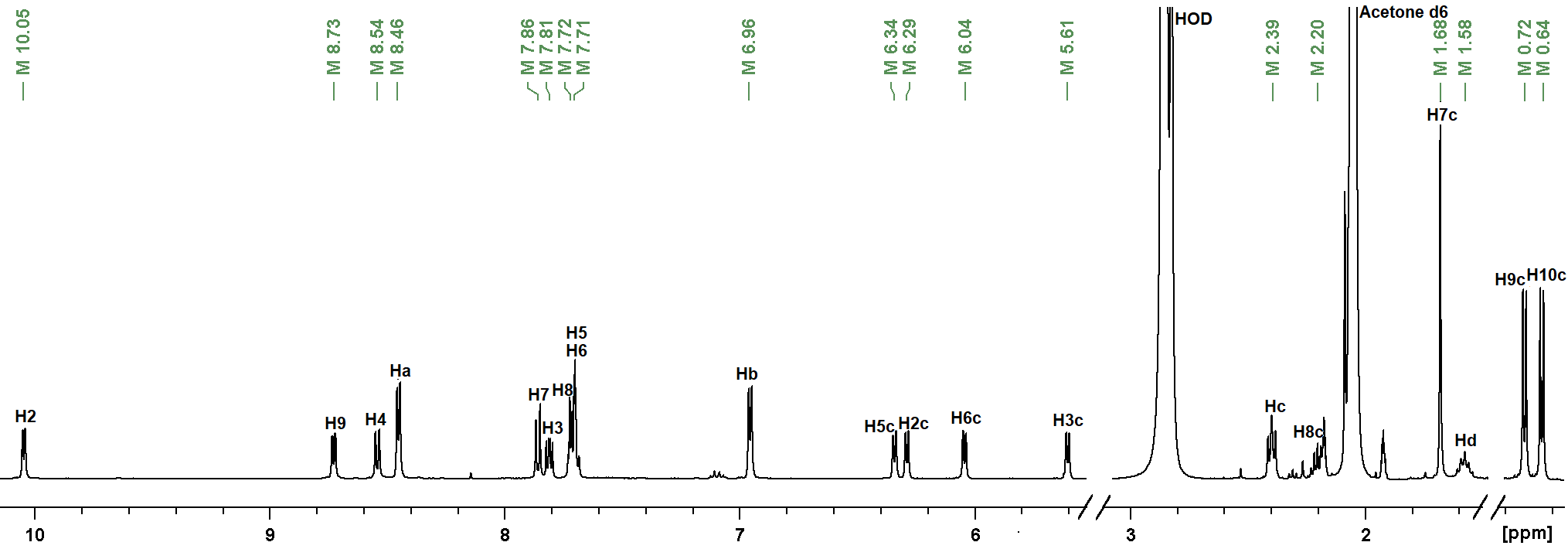
**

**Figure S9**: ^1^H NMR spectrum of the complex (**3**) in acetone-d_6_ at 298 K.

**
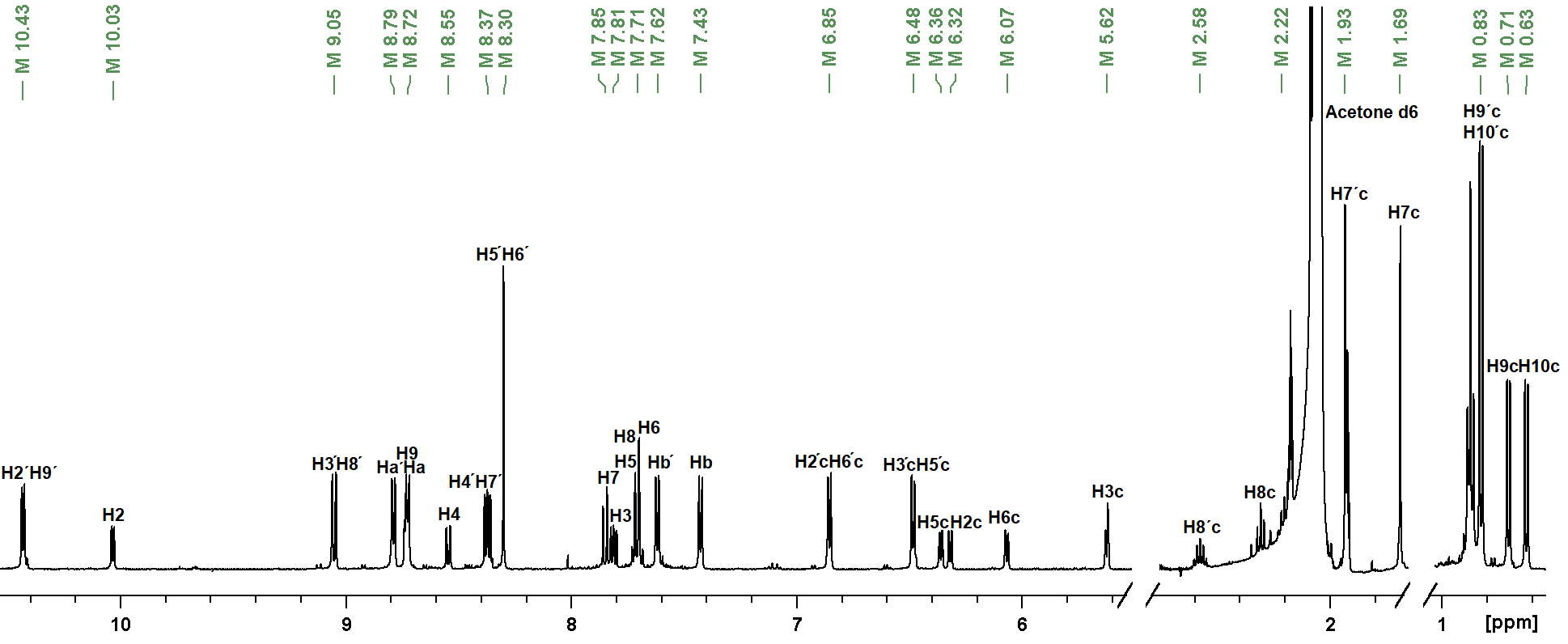
**

**Figure S10**: ^1^H NMR spectrum of the complex (**4**) in acetone-d_6_ at 298 K.

**
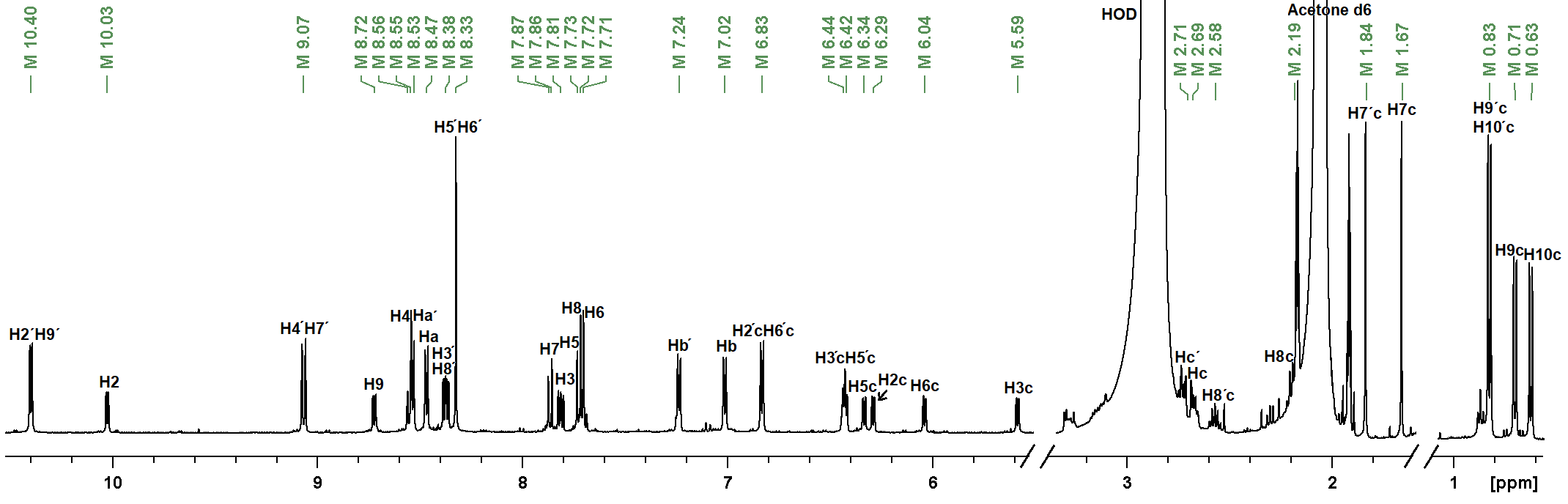
**

**Figure S11**: ^1^H NMR spectrum of the complex (**5**) in acetone-d_6_ at 298 K.

**
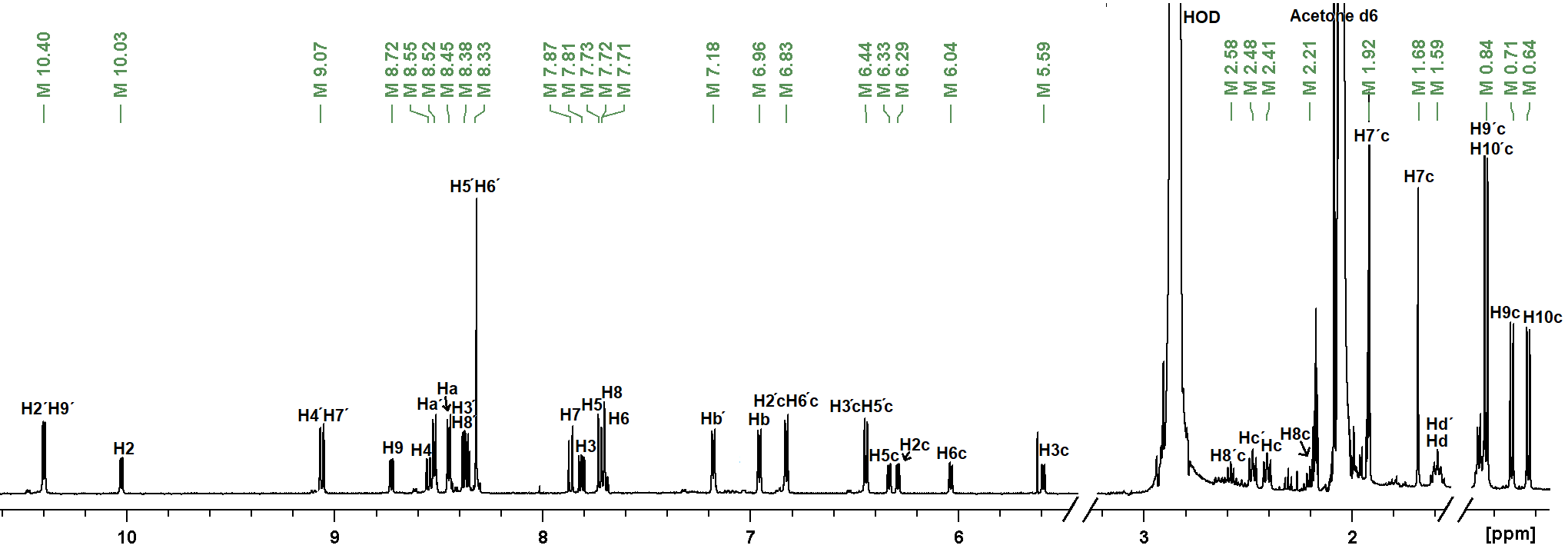
**

**Figure S12**: ^1^H NMR spectrum of the complex (**6**) in acetone-d_6_ at 298 K.


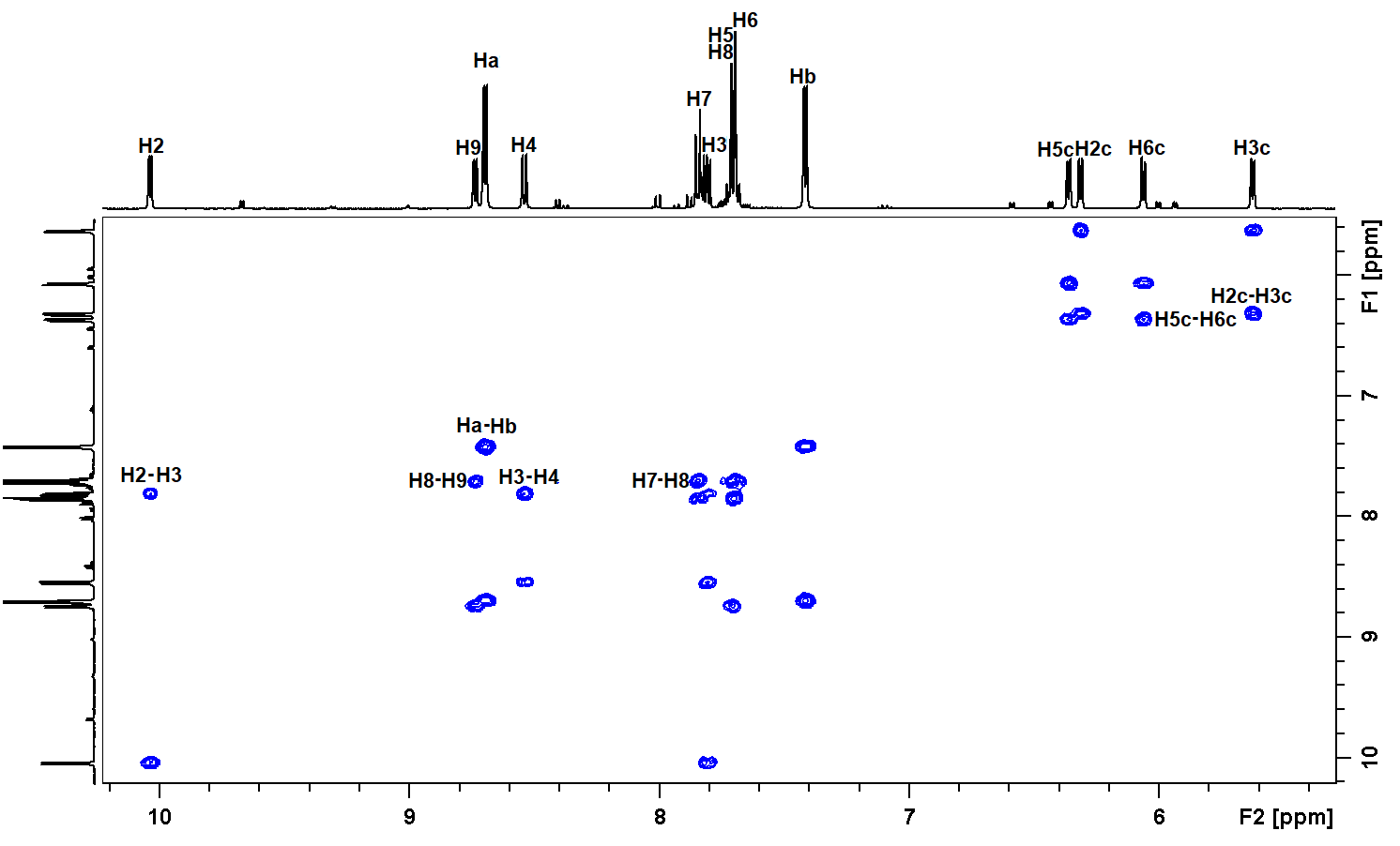


**Figure S13**: ¹H–¹H COSY NMR spectrum of complex (**1**) in acetone-d₆ at 298 K, showing proton–proton cross-peak assignments.


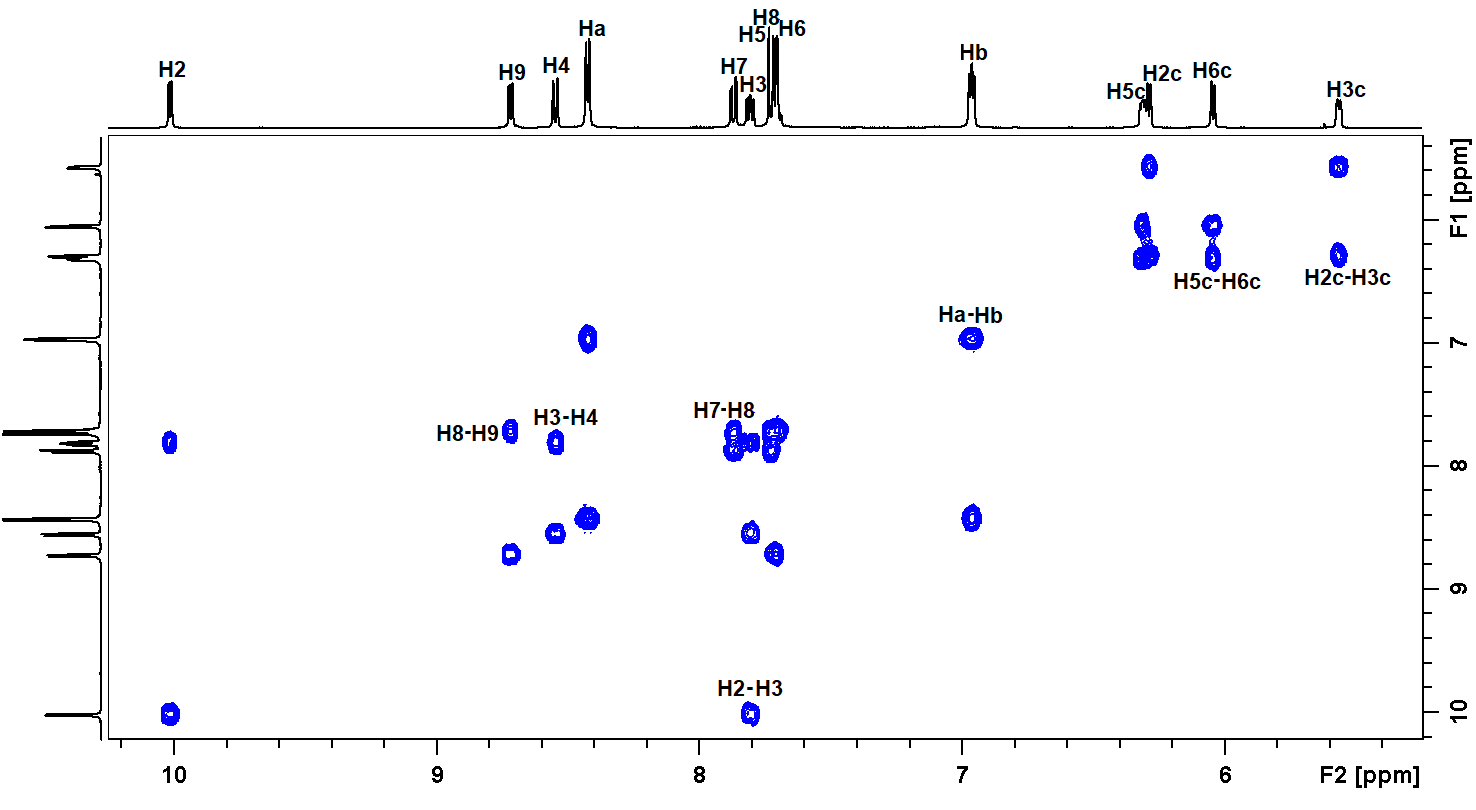


**Figure S14**: ¹H–¹H COSY NMR spectrum of complex (**2**) in acetone-d₆ at 298 K, showing proton–proton cross-peak assignments.


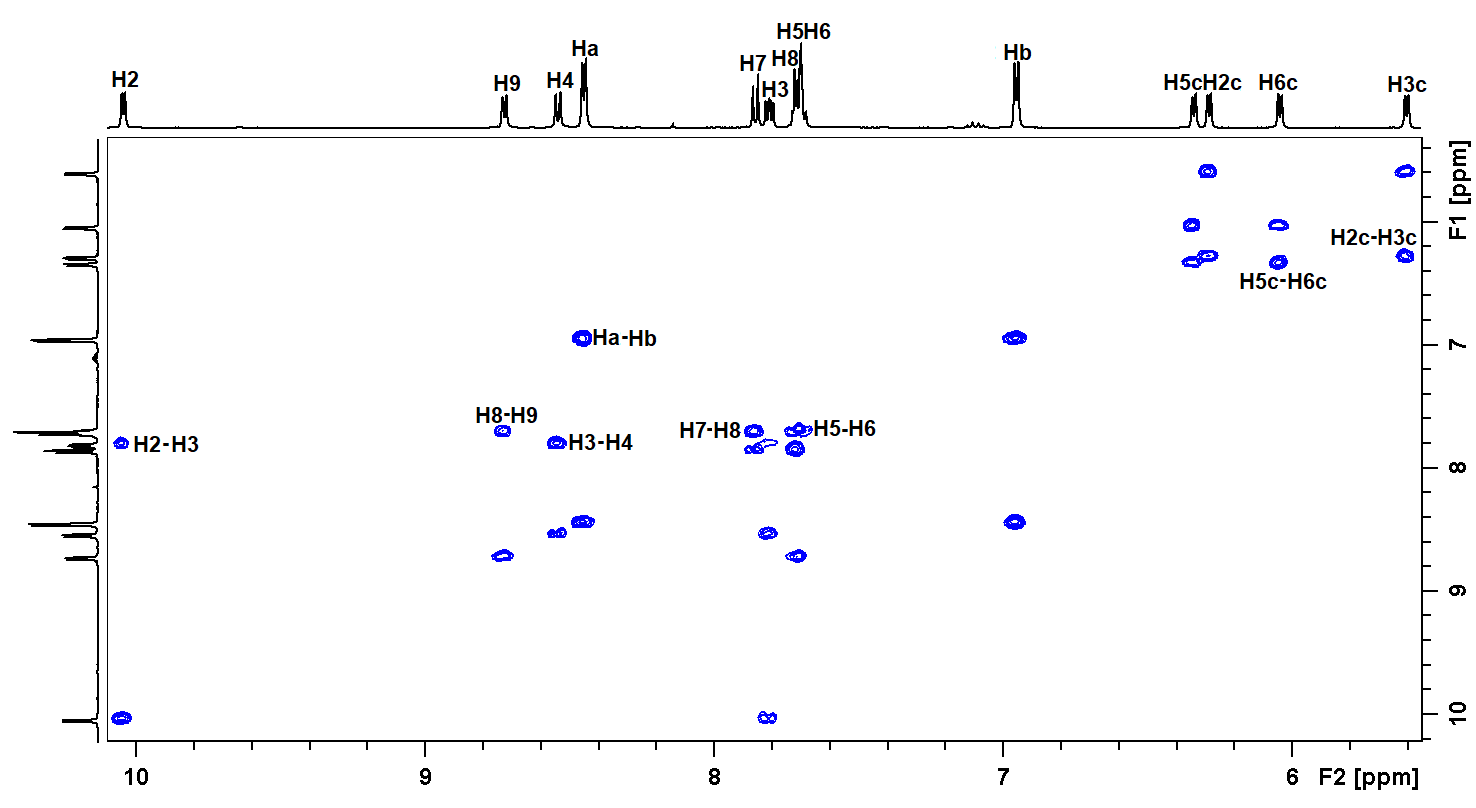


**Figure S15**: ¹H–¹H COSY NMR spectrum of complex (**3**) in acetone-d₆ at 298 K, showing proton–proton cross-peak assignments.


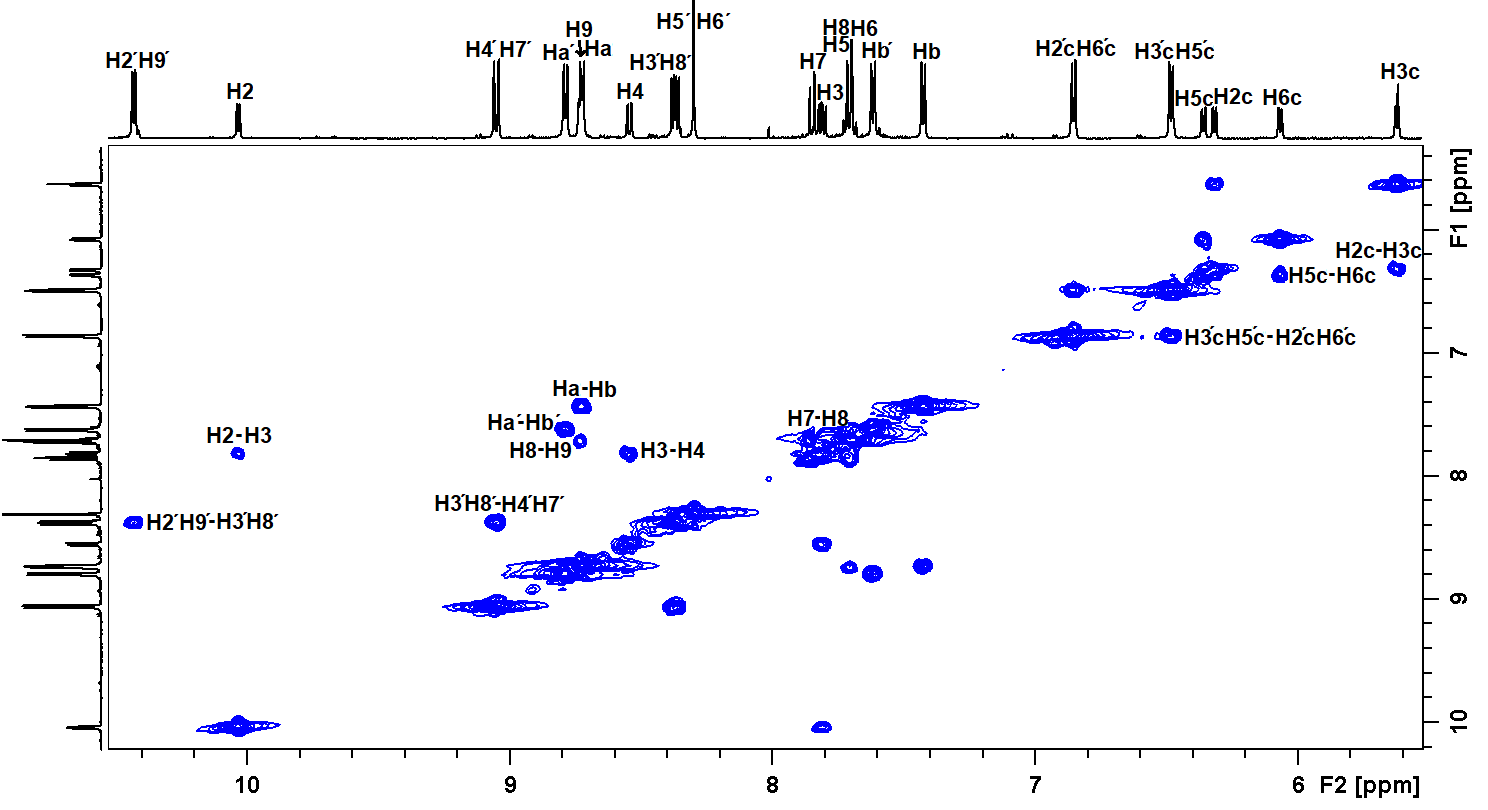


**Figure S16**: ¹H–¹H COSY NMR spectrum of complex (**4**) in acetone-d₆ at 298 K, showing proton–proton cross-peak assignments.


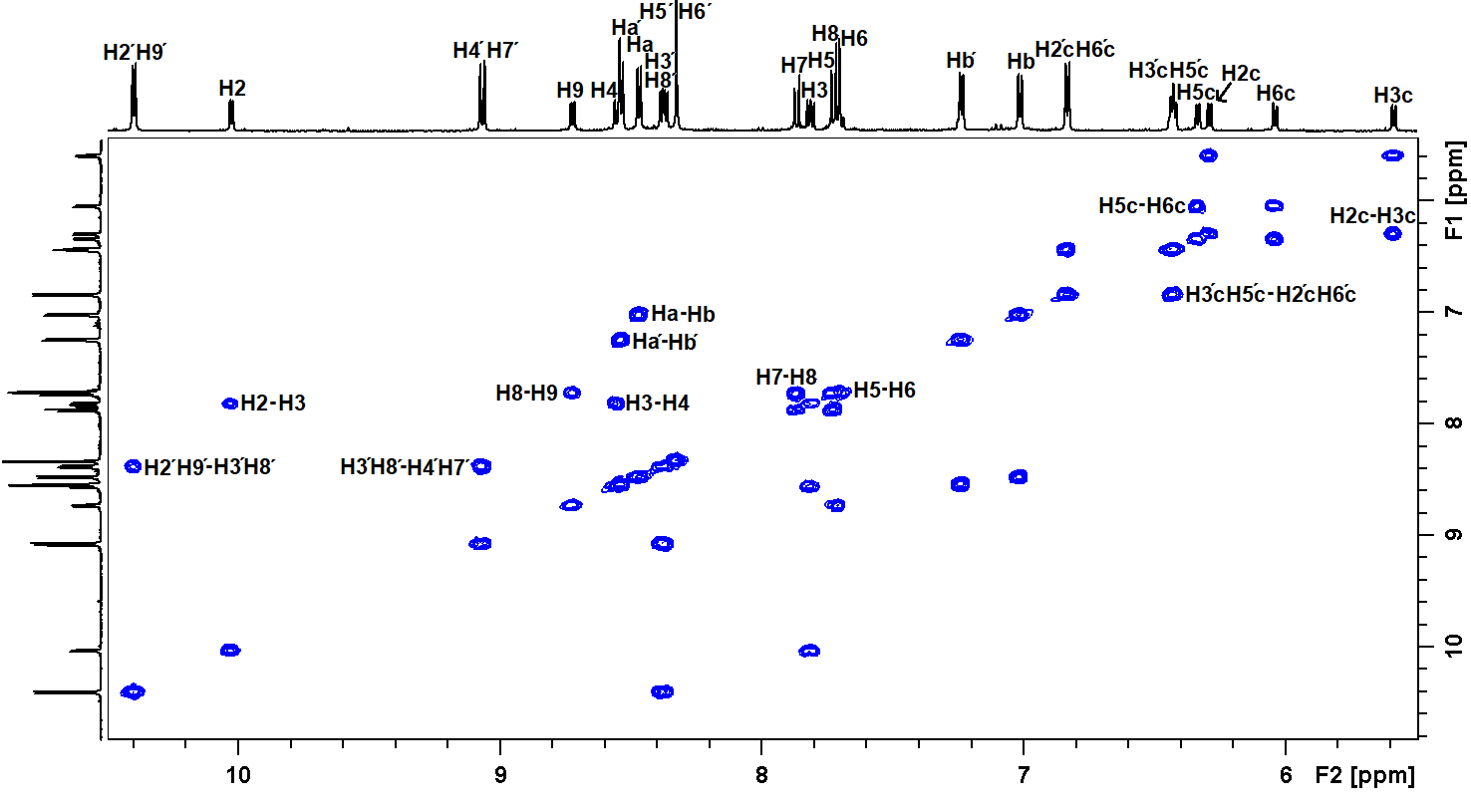


**Figure S17**: ¹H–¹H COSY NMR spectrum of complex (**5**) in acetone-d₆ at 298 K, showing proton–proton cross-peak assignments.


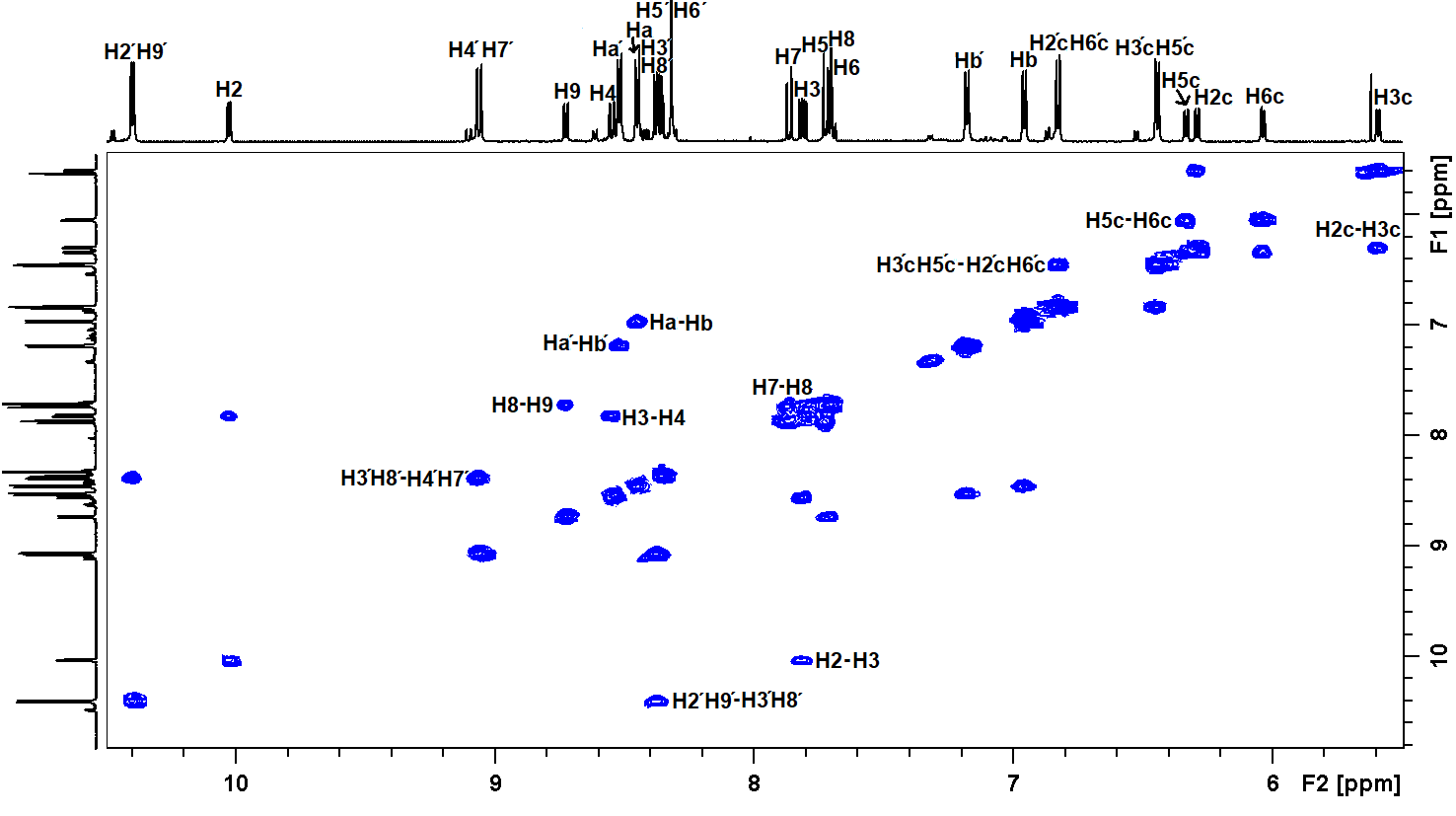


**Figure S18**: ¹H–¹H COSY NMR spectrum of complex (**6**) in acetone-d₆ at 298 K, showing proton–proton cross-peak assignments.


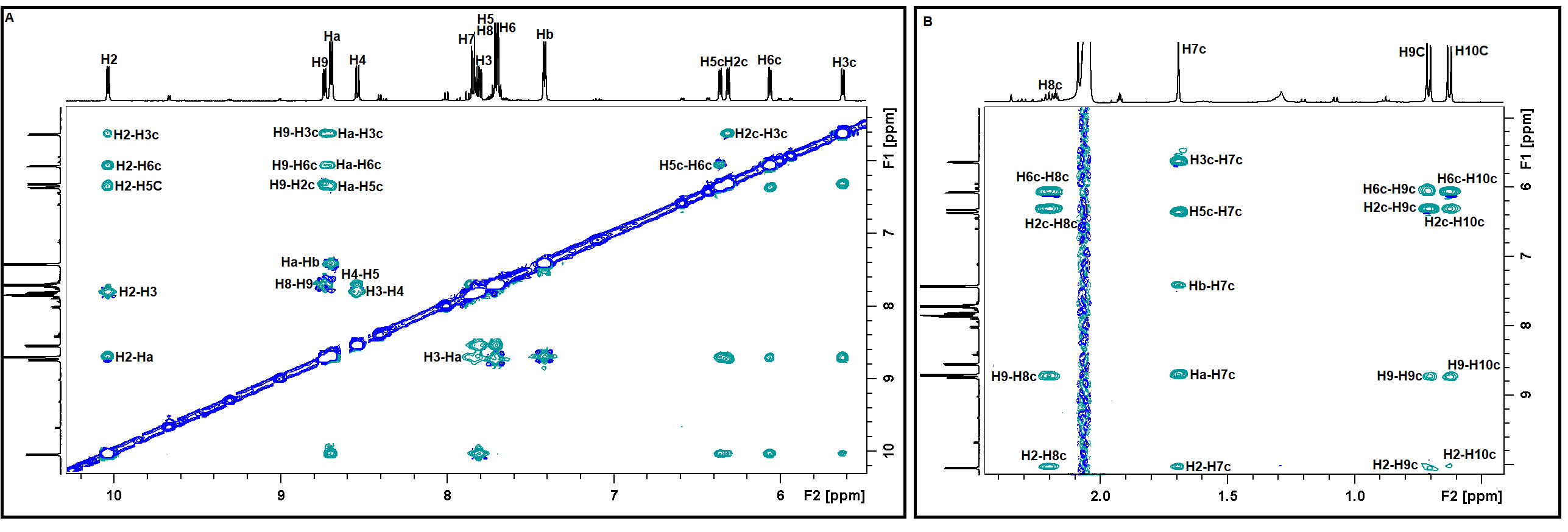


**Figure S19**: ¹H–¹H NOESY NMR spectrum of complex (**1**) in acetone-d₆ at 298 K, showing proton–proton cross-peak assignments. (A) aromatic region; (B) aliphatic region.


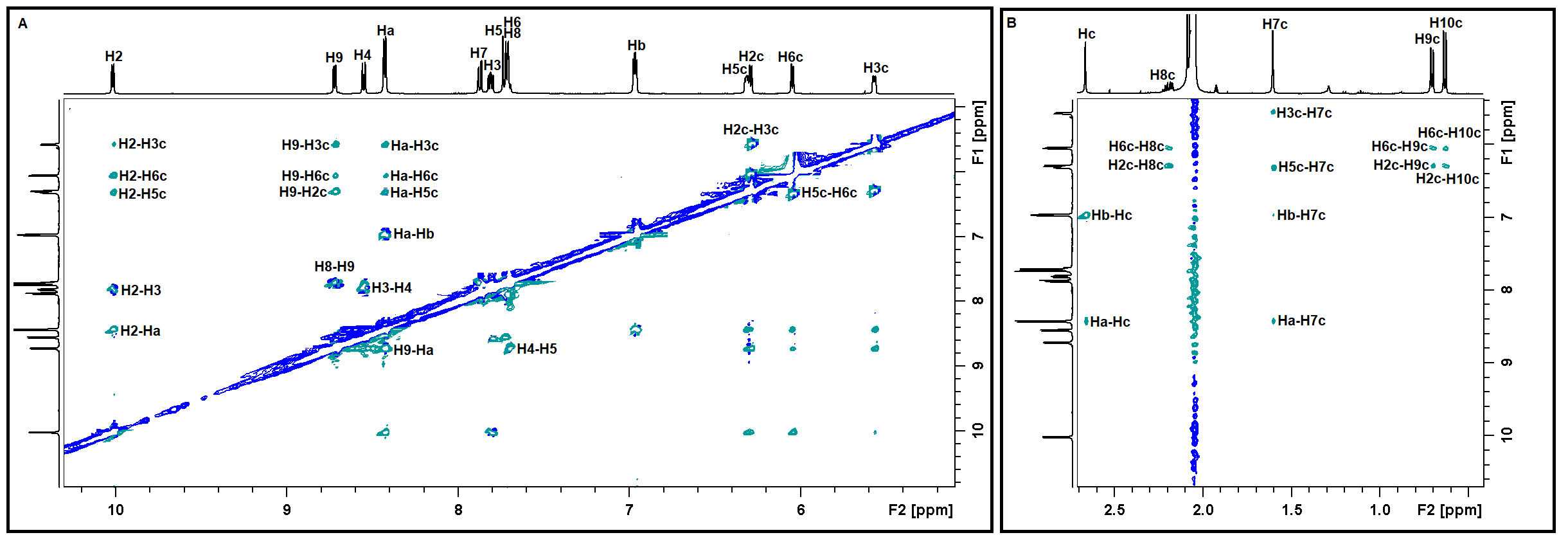


**Figure S20**: ¹H–¹H NOESY NMR spectrum of complex (**2**) in acetone-d₆ at 298 K, showing proton–proton cross-peak assignments. (A) aromatic region; (B) aliphatic region.


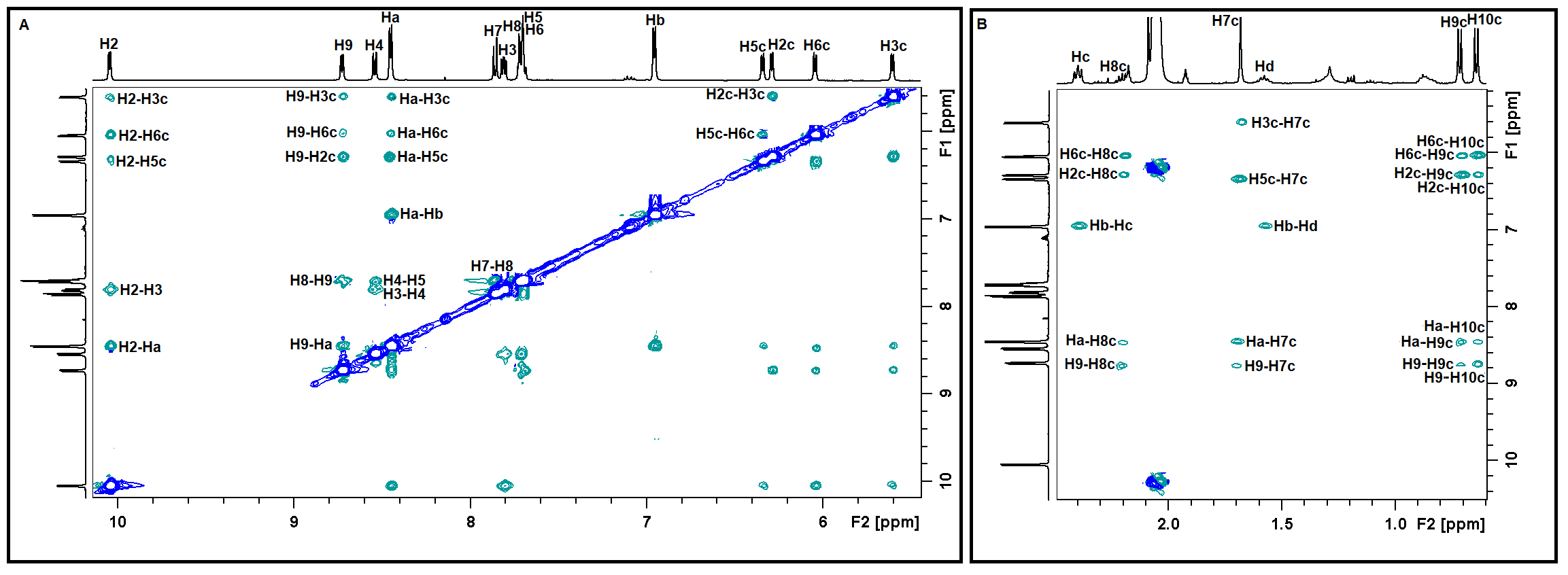


**Figure S21**: ¹H–¹H NOESY NMR spectrum of complex (**3**) in acetone-d₆ at 298 K, showing proton–proton cross-peak assignments. (A) aromatic region; (B) aliphatic region.

**Table S2**: Interligand NOE cross-peaks were observed between protons of the bq, cym, and BL ligands in complexes (**1**) –(**3**) in acetone-d₆ at 298 K (s: strong; m: medium; w: weak).

| bq-cym | BL-cym | bq-BL |
| --- | --- | --- |
| H2-H5c (s) | Ha-H3c (s) | H2-Ha (s) |
| H2-H6c (s) | Ha-H5c (s) | H9-Ha (s) |
| H2-H3c (w) | Ha-H6c (w) |  |
| H2-H9c (w) | Ha-H7c (s) |  |
| H2-H10c (w) | Ha-H8c (w) |  |
| H9-Ha (m) | Hb-H7c (m) |  |
| H9-H2c (s) |  |  |
| H9-H3c (s) |  |  |
| H9-H6c (w) |  |  |
| H9-H7c (w) |  |  |
| H9-H8c (m) |  |  |
| H9-H9c (w) |  |  |
| H9-H10c (w) |  |  |


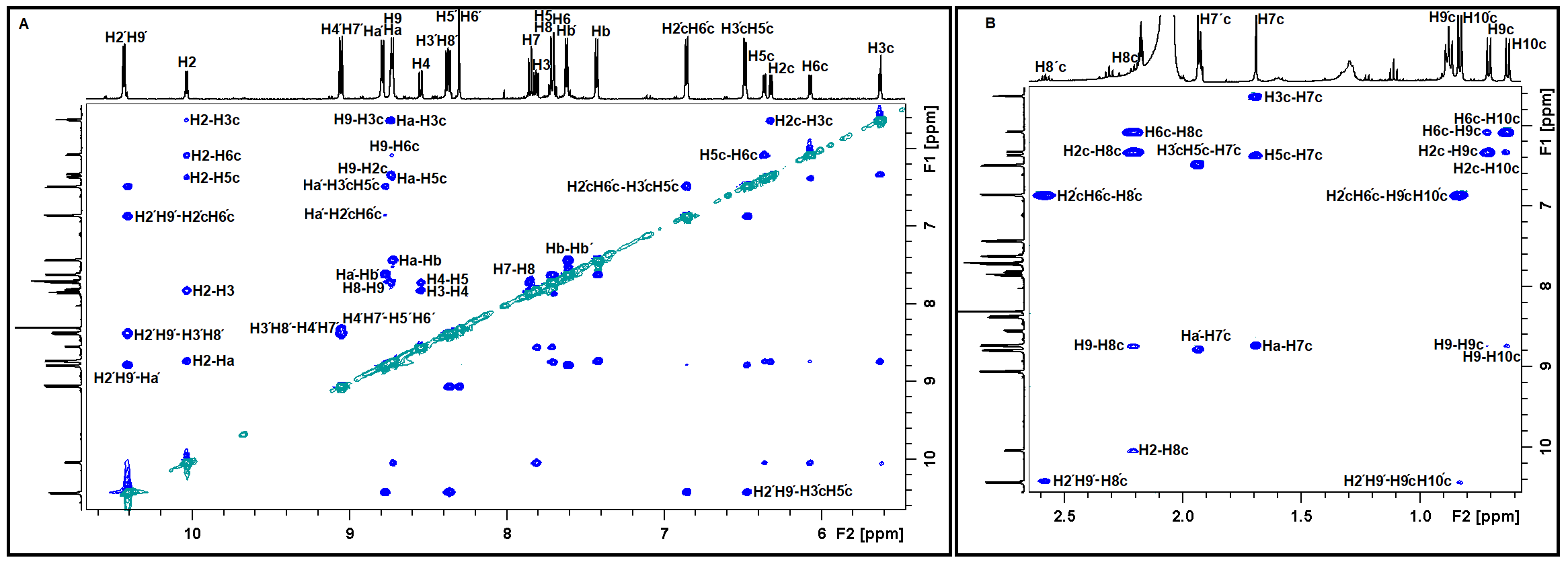


**Figure S22**: ¹H–¹H NOESY NMR spectrum of complex (**4**) in acetone-d₆ at 298 K, showing proton–proton cross-peak assignments. (A) aromatic region; (B) aliphatic region.


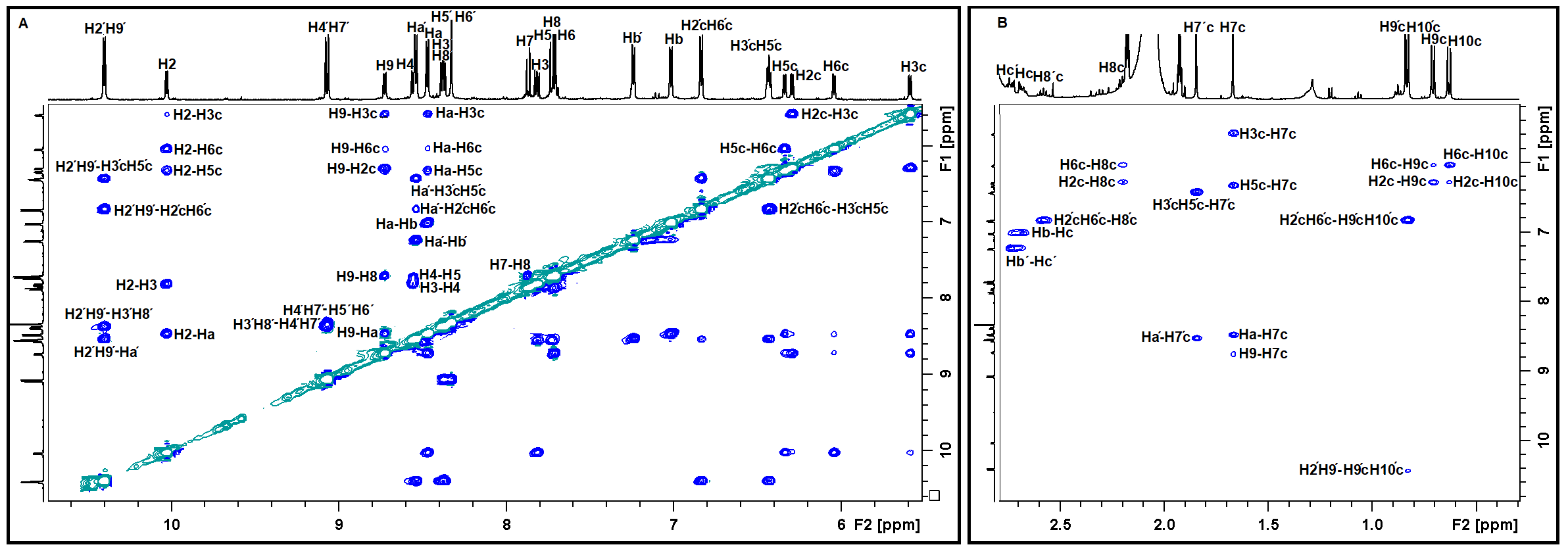


**Figure S23**: ¹H–¹H NOESY NMR spectrum of complex (**5**) in acetone-d₆ at 298 K, showing proton–proton cross-peak assignments. (A) aromatic region; (B) aliphatic region.


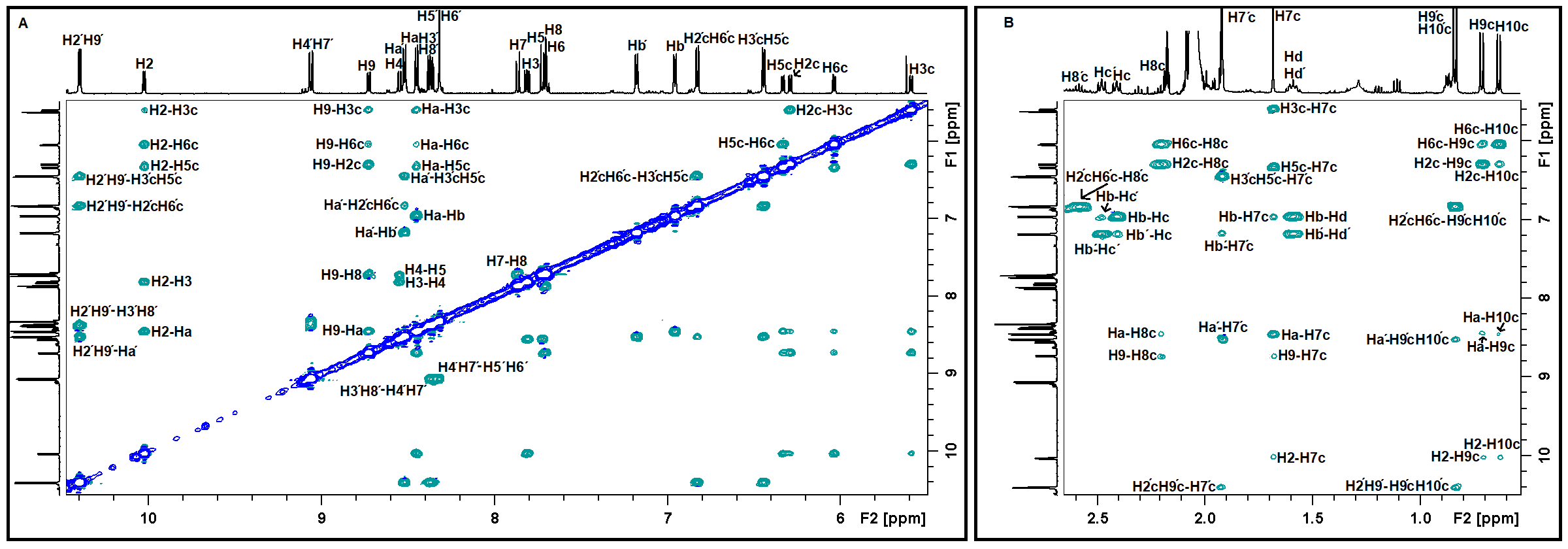


**Figure S24**: ¹H–¹H NOESY NMR spectrum of complex (**6**) in acetone-d₆ at 298 K, showing proton–proton cross-peak assignments. (A) aromatic region; (B) aliphatic region.

**Table S3**: Interligand NOE cross-peaks were observed between protons of the bq, phe, cym, cym΄and BL ligands in complexes (**4**)(PF₆)_2_–(**6**)(PF₆)_2_ in acetone-d₆ at 298 K (s: strong; m: medium; w: weak).

| bq-cym | bq-BL | BL-cym | phe-cym΄ | phe-BL | BL-cym΄ |
| --- | --- | --- | --- | --- | --- |
| H2-H3c (w) | H2-Ha (s) | Ha-H3c (s) | H2’H9΄-H2’cH6΄c (s) | H2’H9΄-Ha΄ (s) | Ha΄-H2’cH6΄c (w) |
| H2-H5c (s) | H9-Ha (s) | Ha-H5c (s) | H2’H9΄-H3’cH5΄c (s) |  | Ha΄-H3’cH5΄c (s) |
| H2-H6c (s) |  | Ha-H6c (w) | H2’H9΄-H7’c (w) |  | Ha΄-H7’c (s) |
| H2-H7c (w) |  | Ha-H7c (s) | H2’H9΄-H9’cH10΄c (m) |  | Ha΄-H9’c H10’c (w) |
| H2-H9c (w) |  | Ha-H8c (w) |  |  | Hb΄-H7΄c (m) |
| H2-H10c (w) |  | Ha-H9c (w) |  |  |  |
| H9-H2c (s) |  | Ha-H10c (w) |  |  |  |
| H9-H3c (s) |  | Hb-H7c (m) |  |  |  |
| H9-H6c (w) |  |  |  |  |  |
| H9-H7c (w) |  |  |  |  |  |
| H9-H8c (m) |  |  |  |  |  |
| H9-H9c (w) |  |  |  |  |  |
| H9-H10c (w) |  |  |  |  |  |


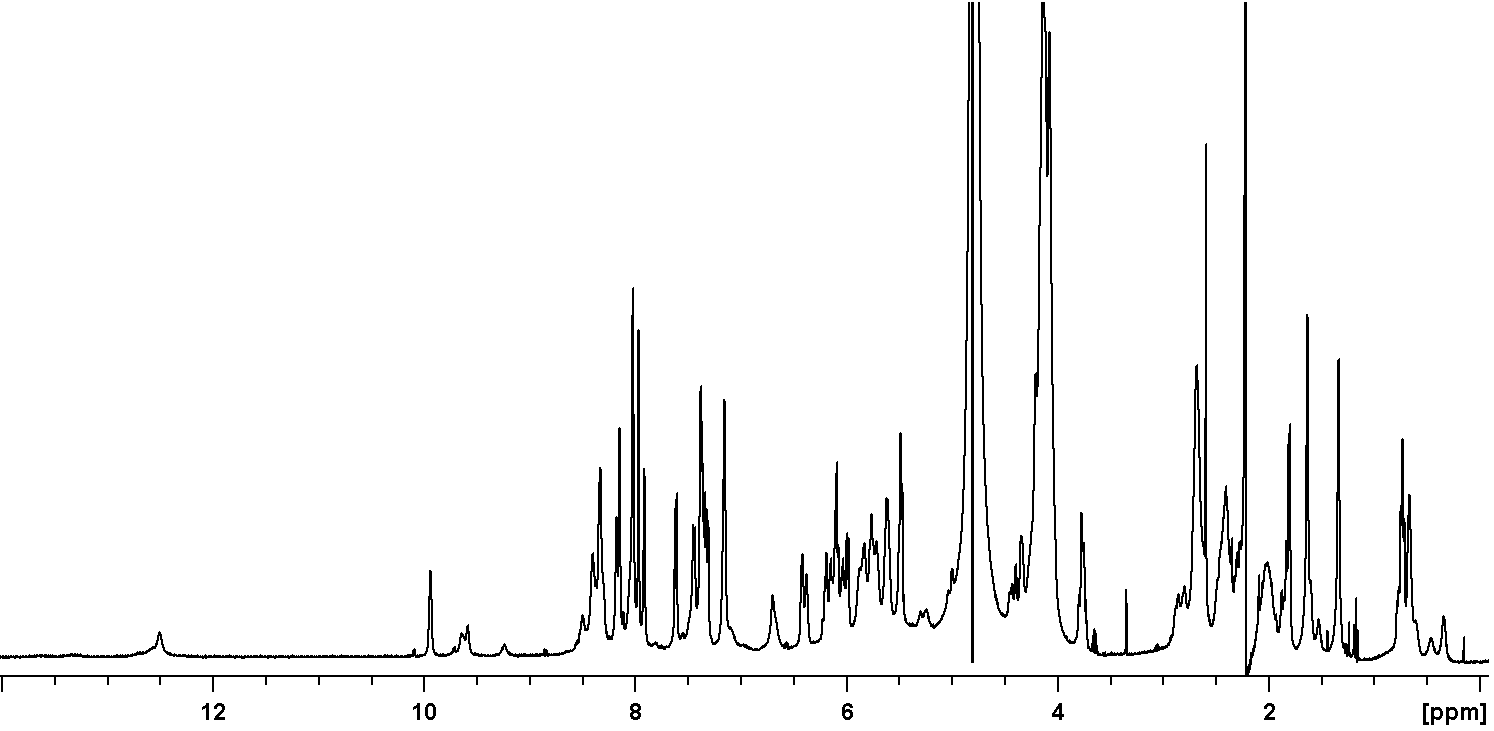


**Figure S25**: ^1^H NMR spectrum of d(5′-CGCGAATTGGCC-3′)_2_ upon addition of complex (**4a**) at r = 0.5 in H_2_O/ D_2_O 9:1 (buffer phosphate 100 mM, pH = 7.0) at 298 K, 500 MHz.


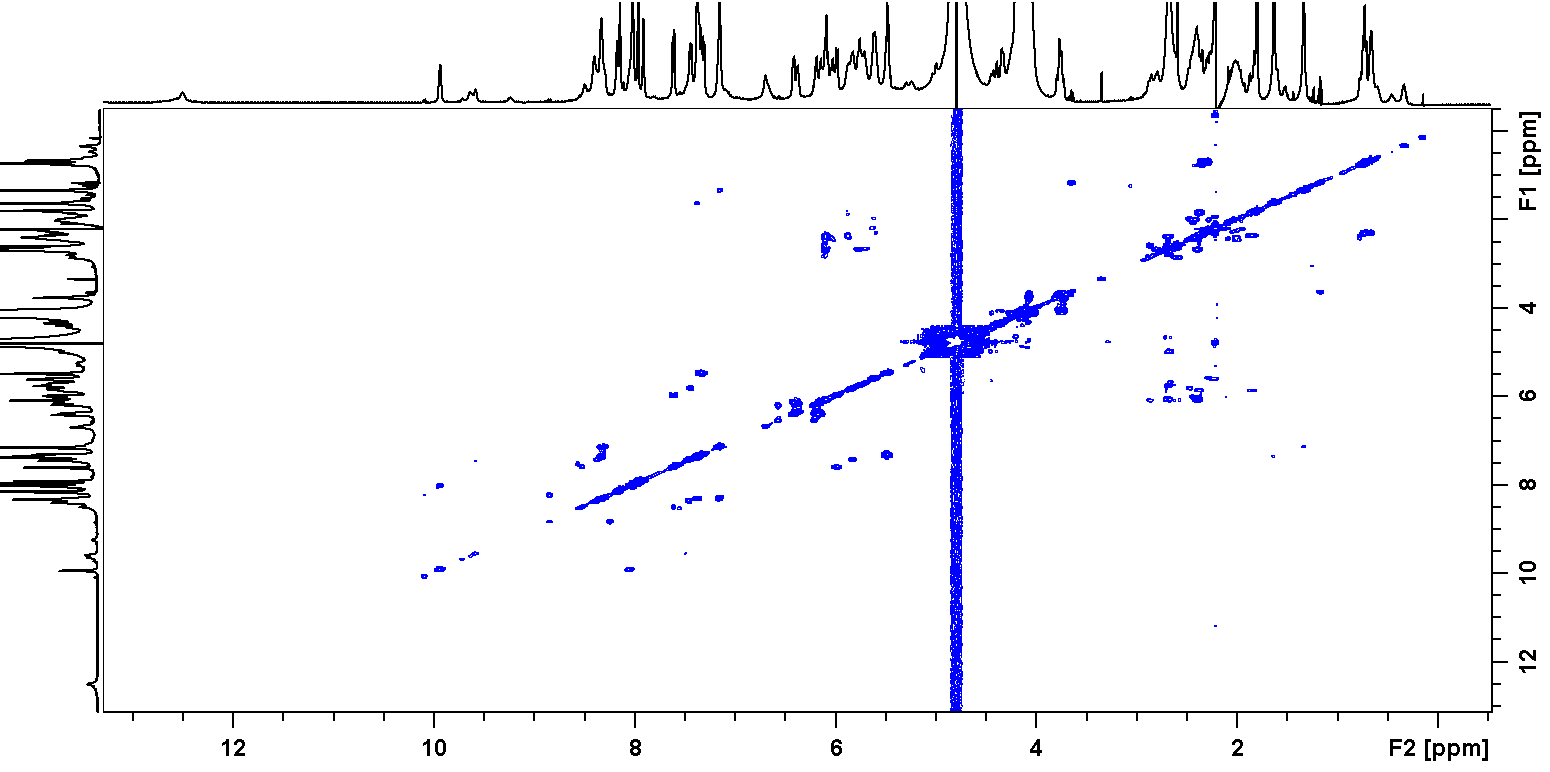


**Figure S26**: ^1^H–^1^H COSY spectrum of d(5′-CGCGAATTGGCC-3′)_2_ upon addition of complex (**4a**) at r = 0.5 in H_2_O/ D_2_O 9:1 (buffer phosphate 100 mM, pH = 7.0) at 298 K, 500 MHz.


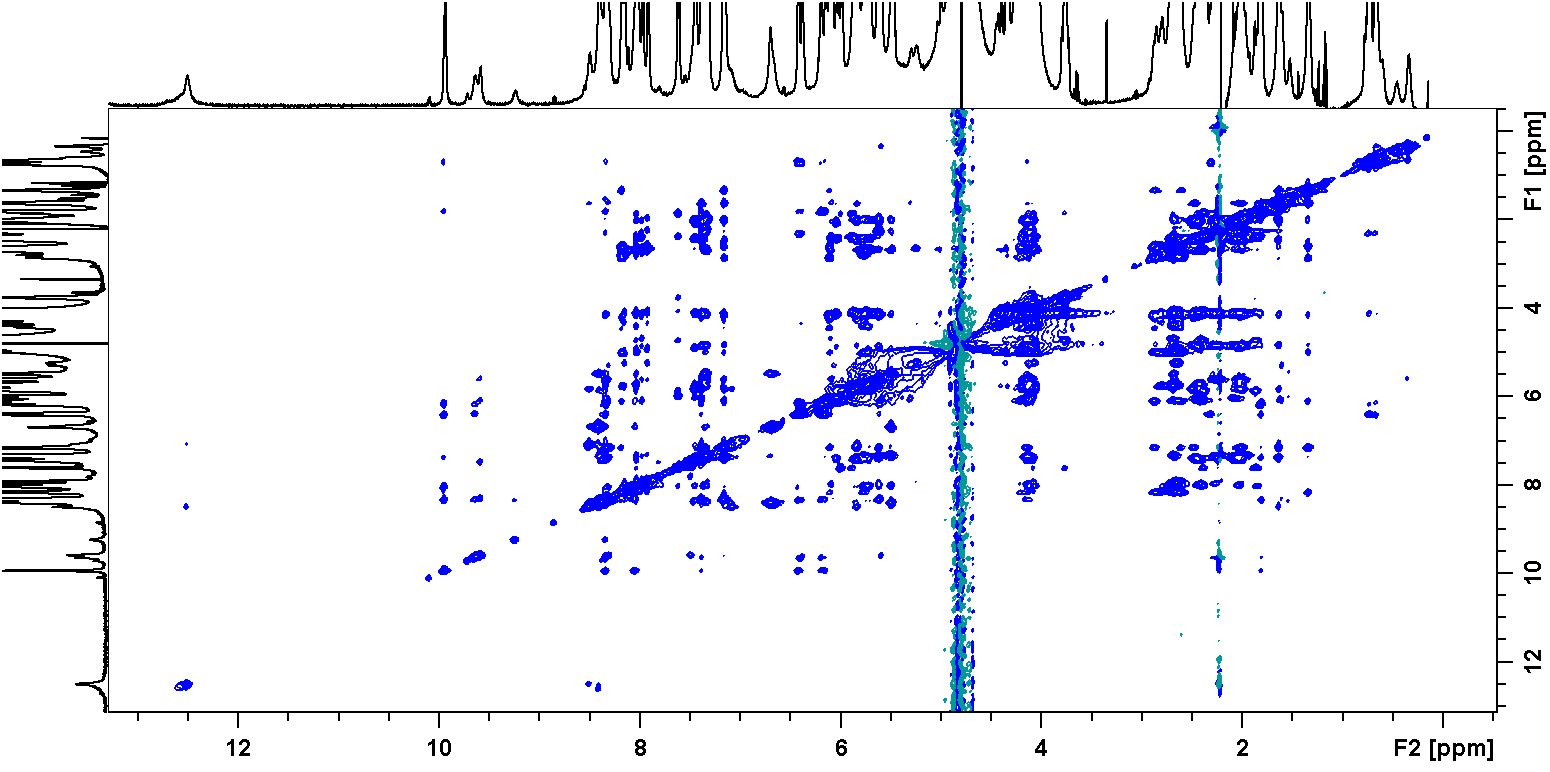


**Figure S27**: ^1^H–^1^H NOESY spectrum of d(5′-CGCGAATTGGCC-3′)_2_ upon addition of complex (**4a**) at r = 0.5 in H_2_O/ D_2_O 9:1 (buffer phosphate 100 mM, pH = 7.0) at 298 K, 500 MHz.


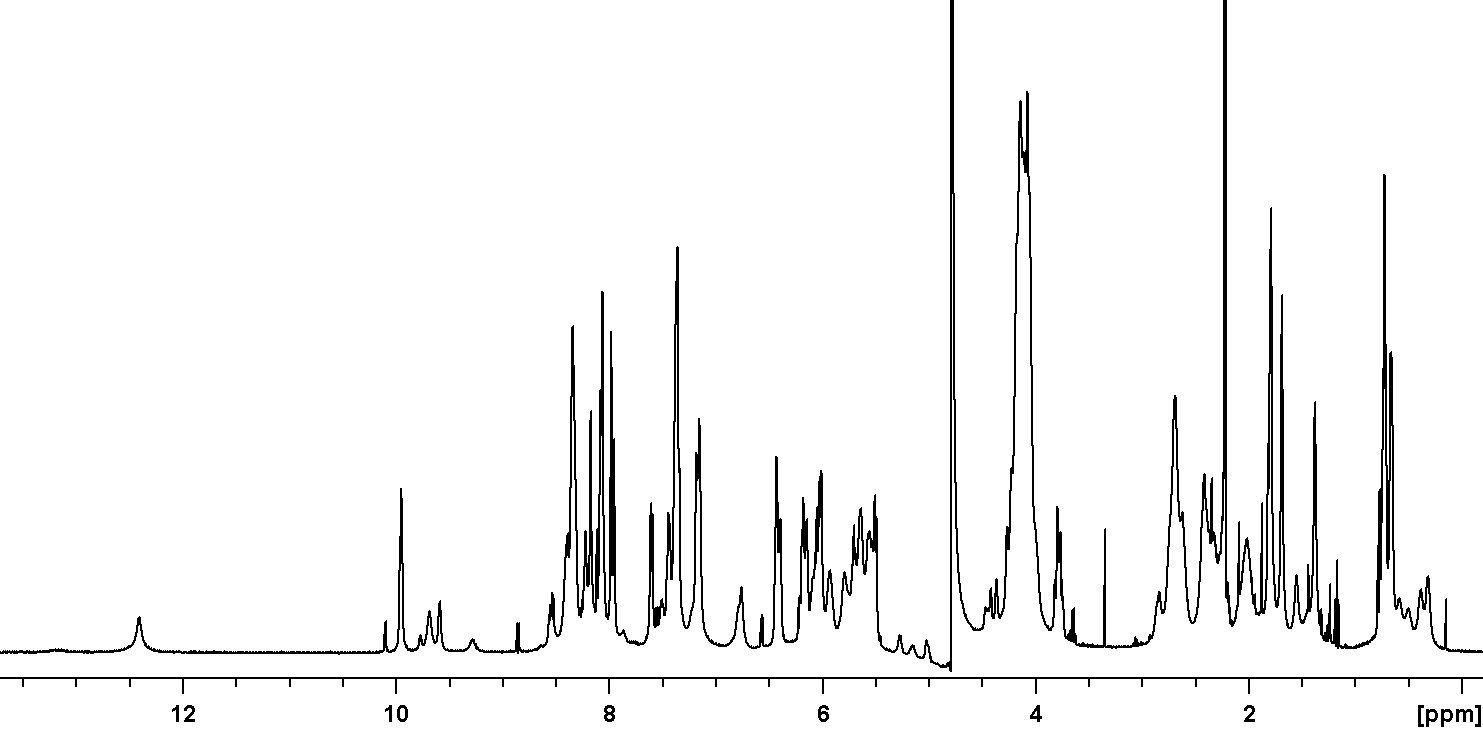


**Figure S28**: ^1^H NMR spectrum of d(5′-CGCGAATTGGCC-3′)_2_ upon addition of complex (**4a**) at r = 1 in H_2_O/ D_2_O 9:1 (buffer phosphate 100 mM, pH = 7.0) at 298 K, 500 MHz.


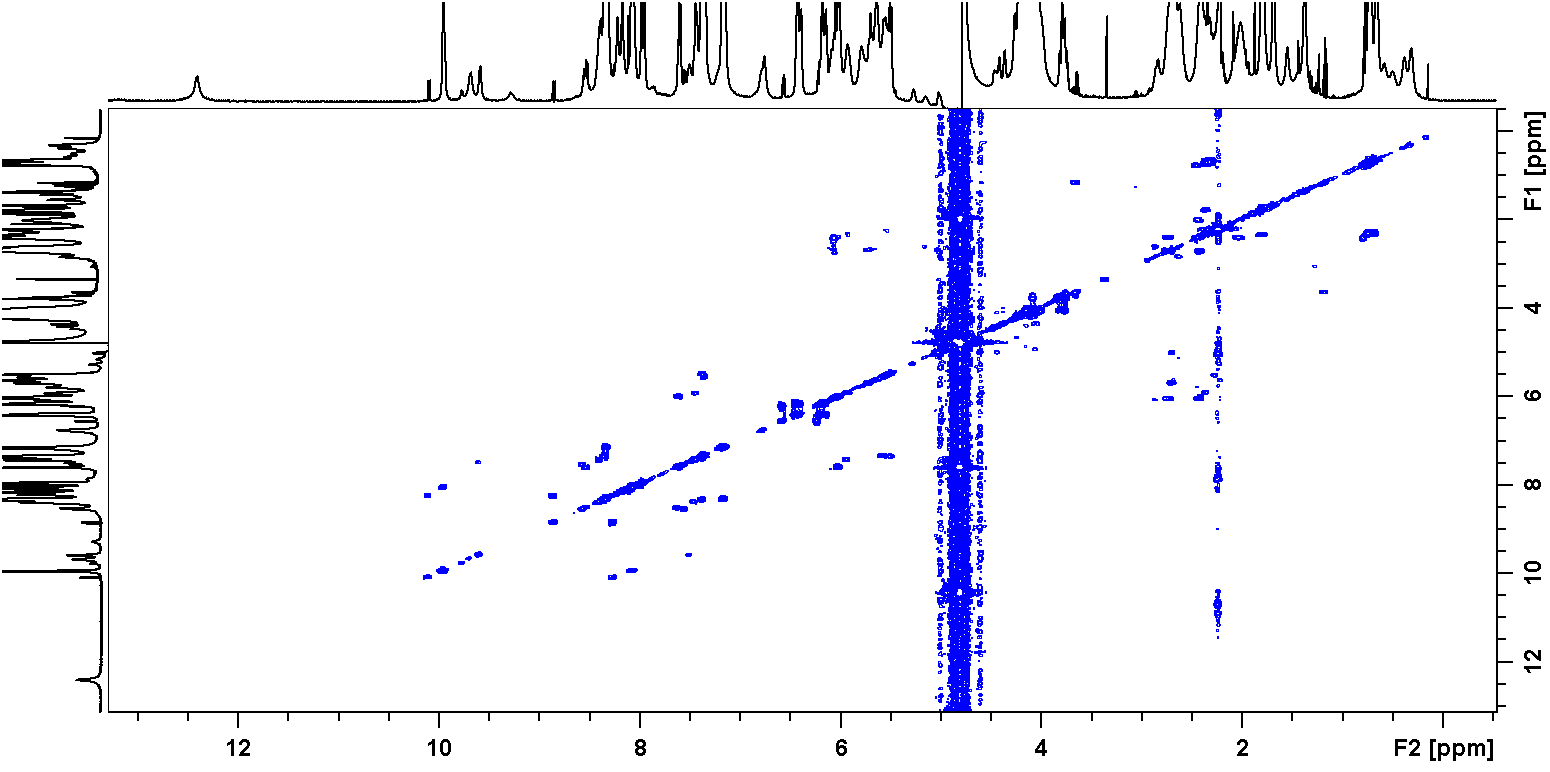


**Figure S29**: ^1^H–^1^H COSY spectrum of d(5′-CGCGAATTGGCC-3′)_2_ upon addition of complex (**4a**) at r = 1 in H_2_O/ D_2_O 9:1 (buffer phosphate 100 mM, pH = 7.0) at 298 K, 500 MHz.


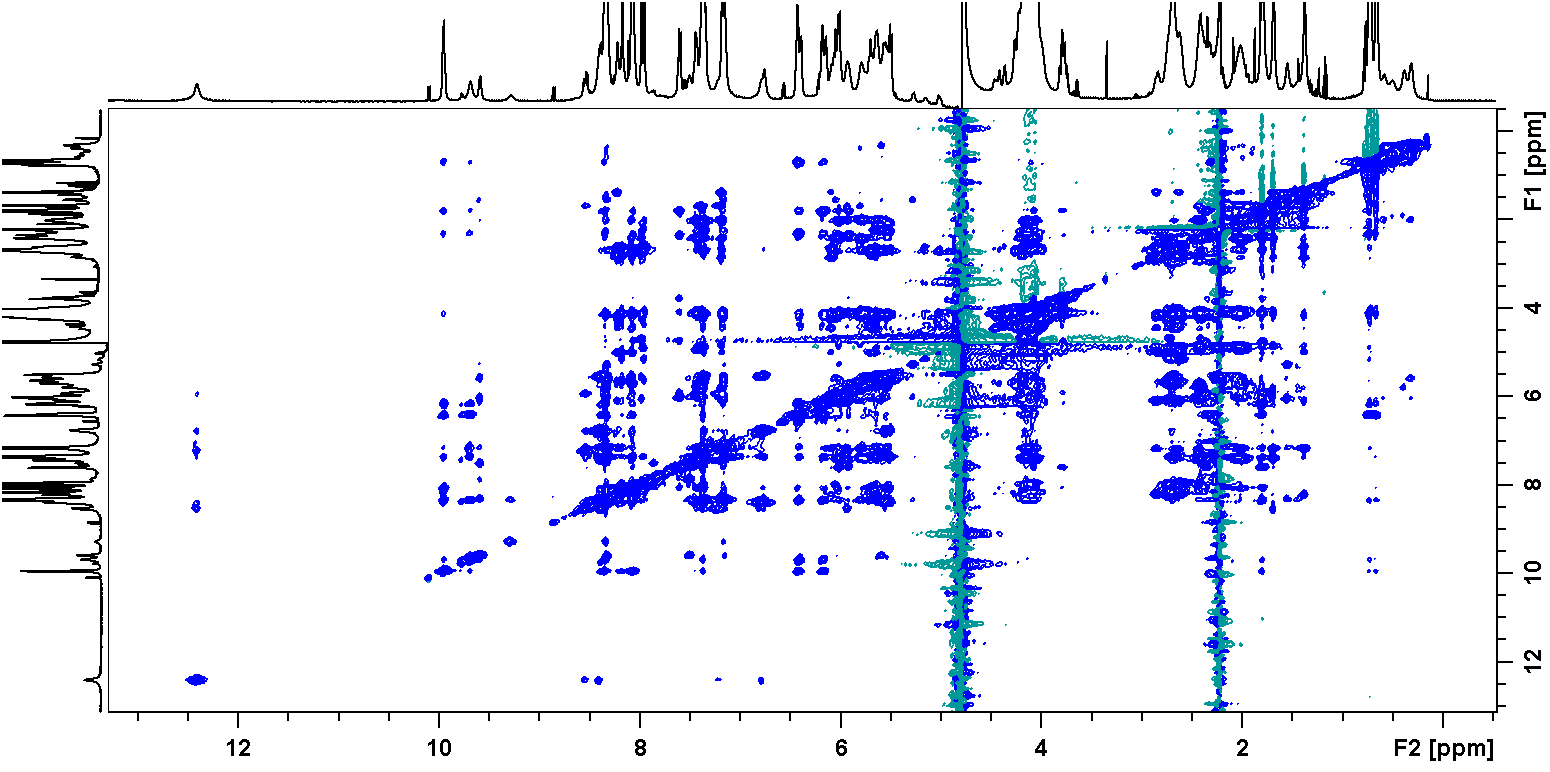


**Figure S30**: ^1^H–^1^H NOESY spectrum of d(5′-CGCGAATTGGCC-3′)_2_ upon addition of complex (**4a**) at r = 1 in H_2_O/ D_2_O 9:1 (buffer phosphate 100 mM, pH = 7.0) at 298 K, 500 MHz.


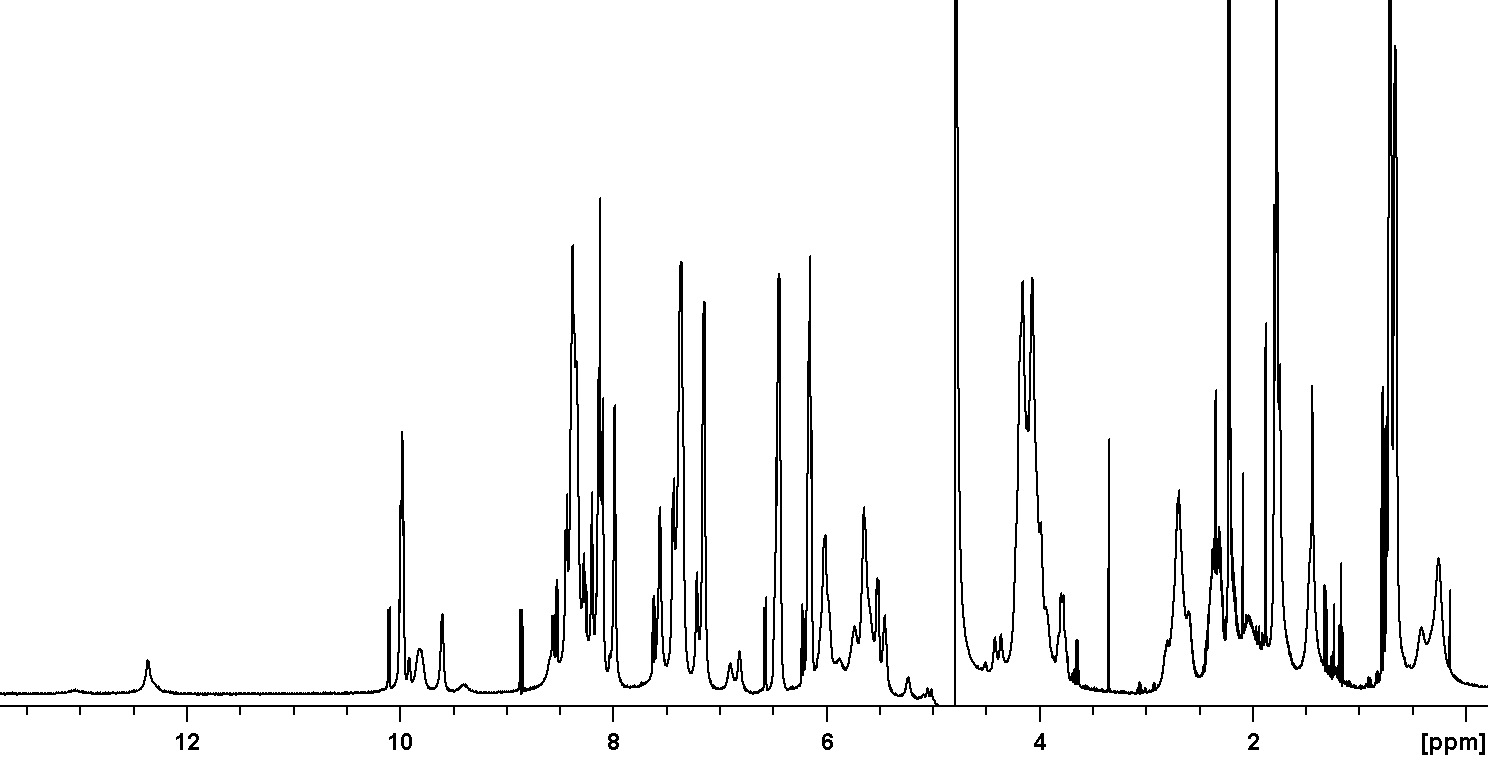


**Figure S31**: ^1^H NMR spectrum of d(5′-CGCGAATTGGCC-3′)_2_ upon addition of complex (**4a**) at r = 2 in H_2_O/ D_2_O 9:1 (buffer phosphate 100 mM, pH = 7.0) at 298 K, 500 MHz.


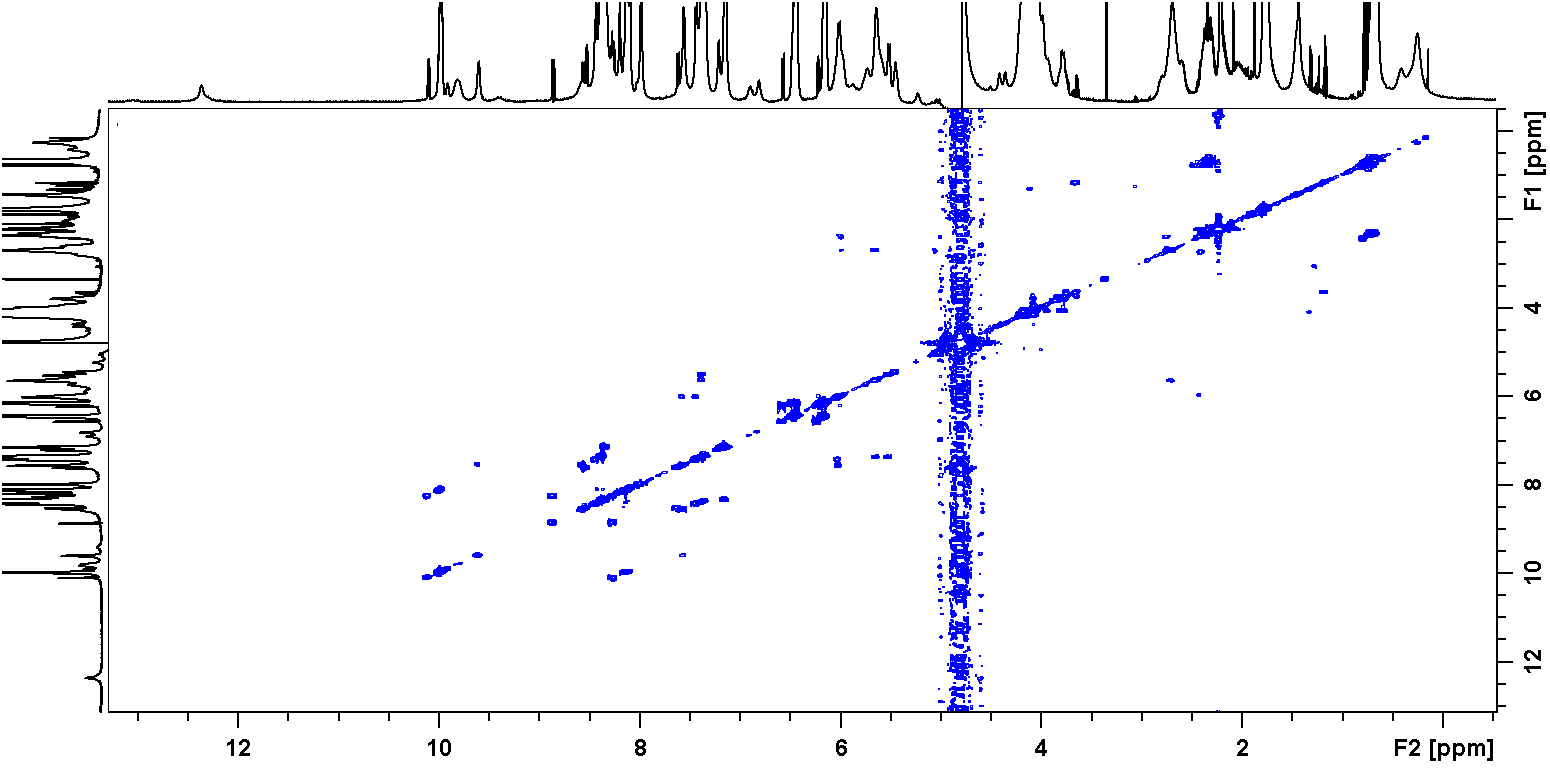


**Figure S32**: ^1^H–^1^H COSY spectrum of d(5′-CGCGAATTGGCC-3′)_2_ upon addition of complex (**4a**) at r = 2 in H_2_O/ D_2_O 9:1 (buffer phosphate 100 mM, pH = 7.0) at 298 K, 500 MHz.


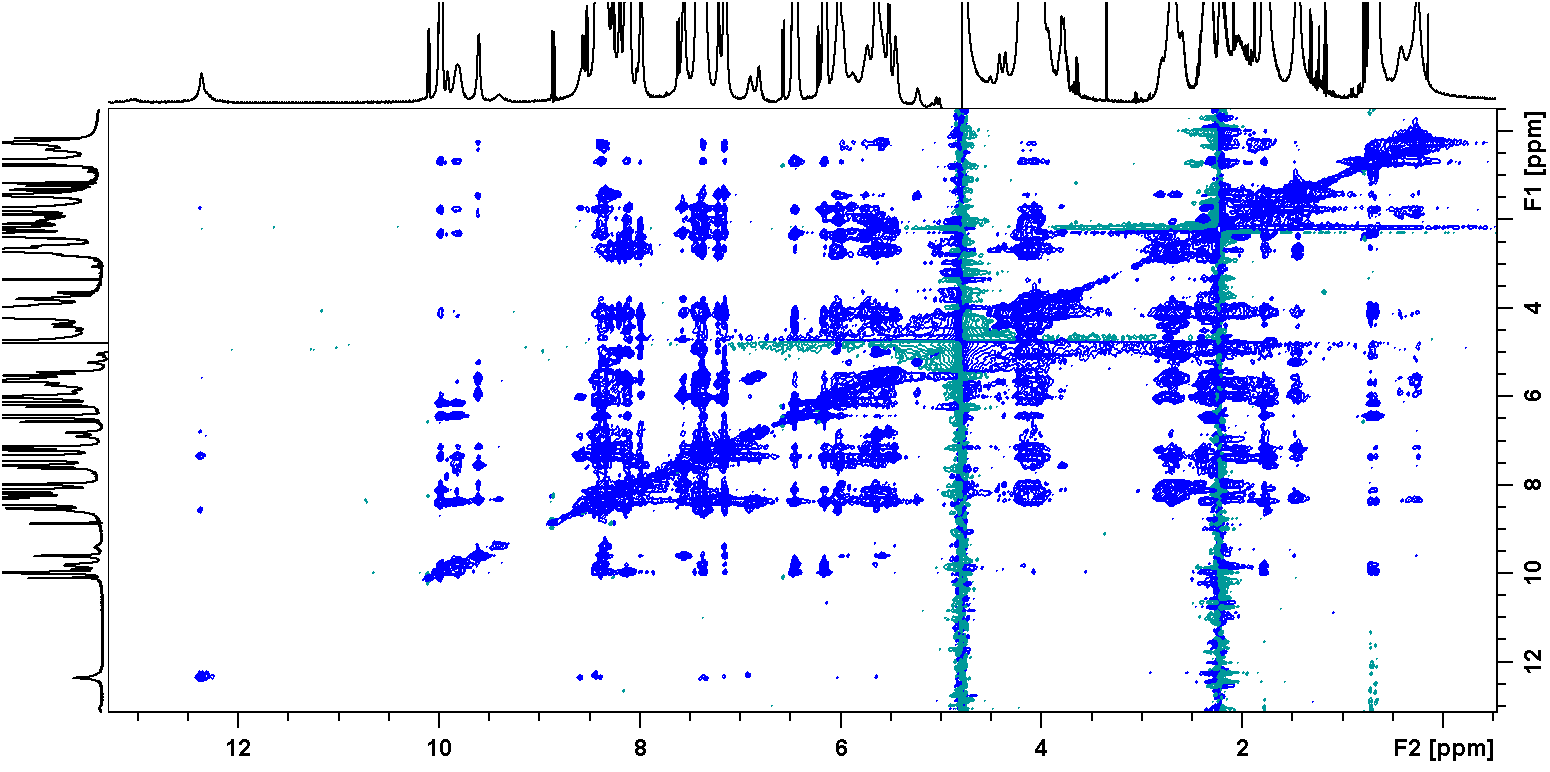


**Figure S33**: ^1^H–^1^H NOSEY spectrum of d(5′-CGCGAATTGGCC-3′)_2_ upon addition of complex (**4a**) at r = 2 in H_2_O/ D_2_O 9:1 (buffer phosphate 100 mM, pH = 7.0) at 298 K, 500 MHz.


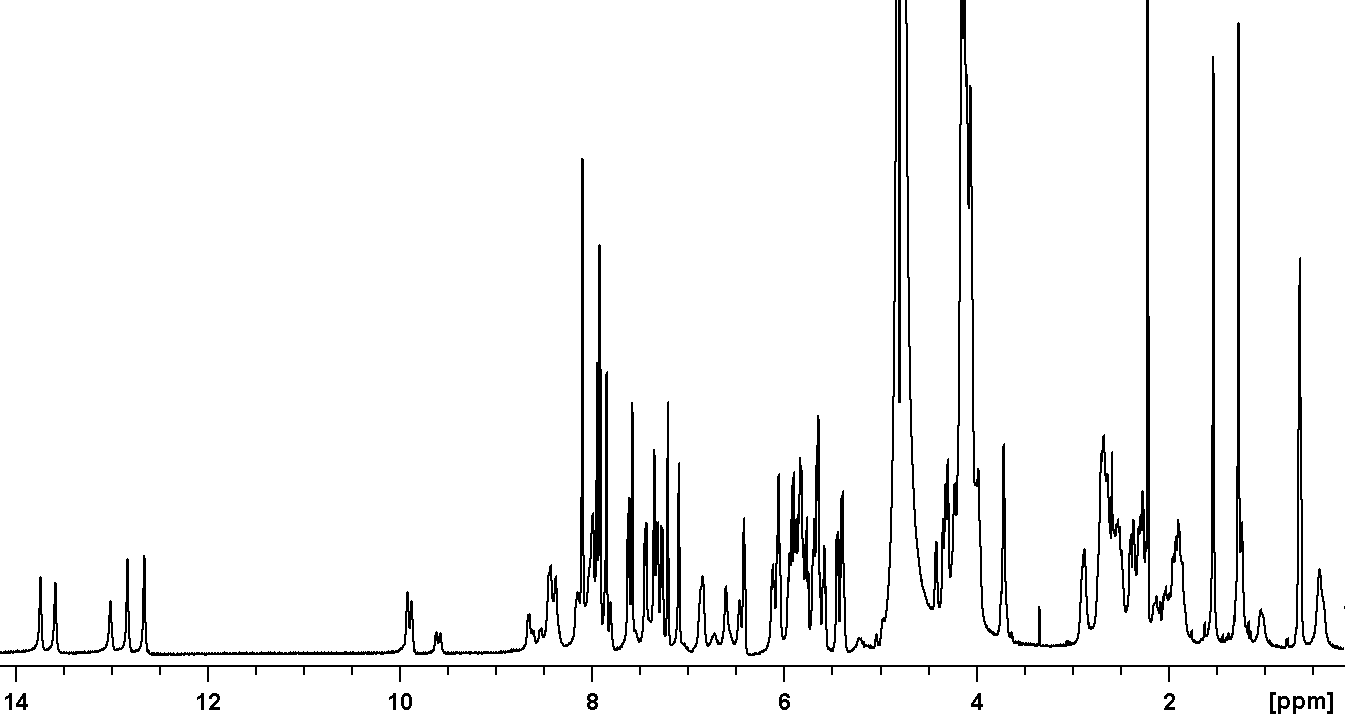


**Figure S34**: ^1^H NMR spectrum of d(5′-CGCGAATTGGCC-3′)_2_ upon addition of complex (**5a**) at r = 0.5 in H_2_O/ D_2_O 9:1 (buffer phosphate 100 mM, pH = 7.0) at 298 K, 500 MHz.


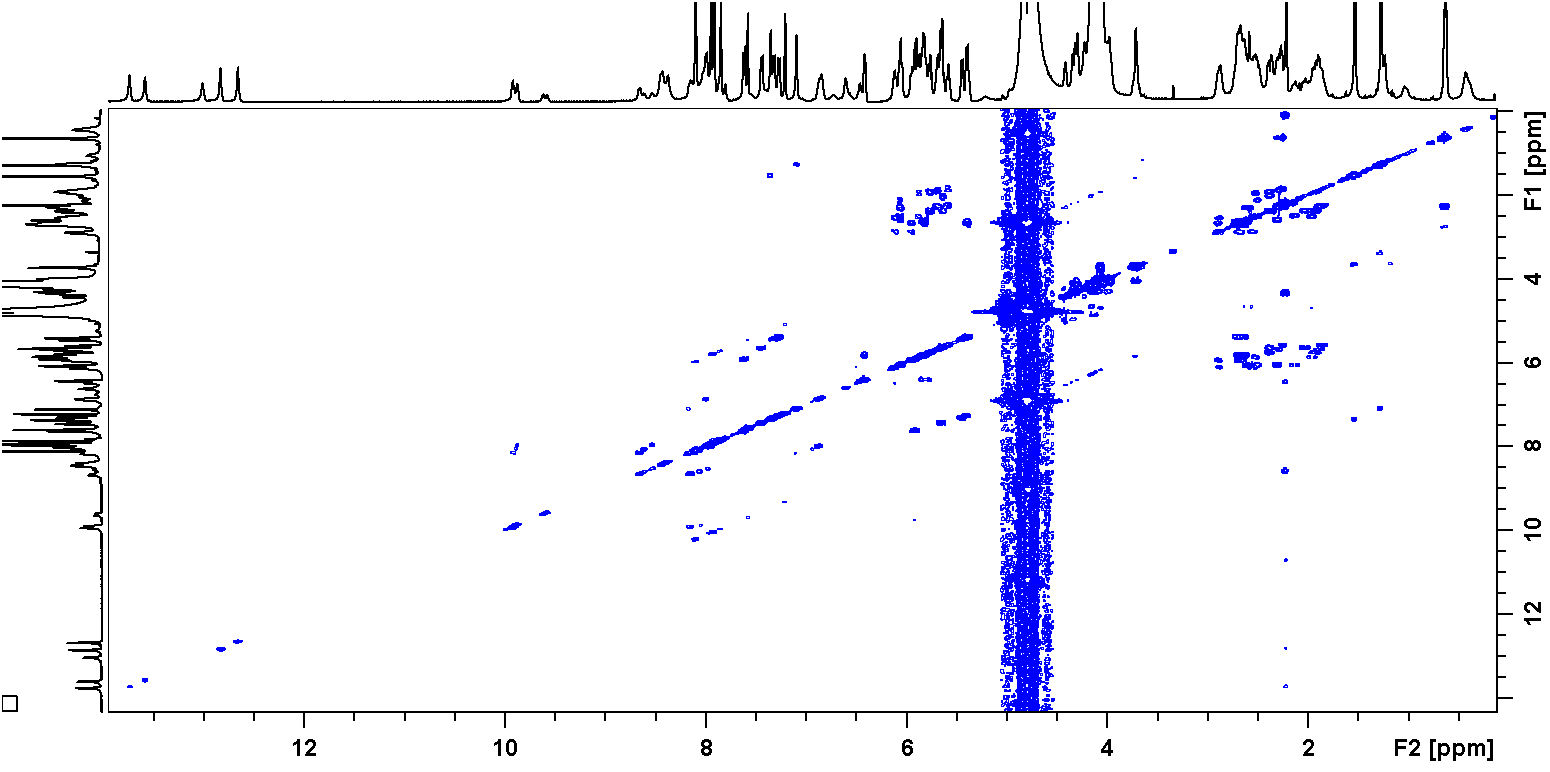


**Figure S35**: ^1^H–^1^H COSY spectrum of d(5′-CGCGAATTGGCC-3′)_2_ upon addition of complex (**5a**) at r = 0.5 in H_2_O/ D_2_O 9:1 (buffer phosphate 100 mM, pH = 7.0) at 298 K, 500 MHz.


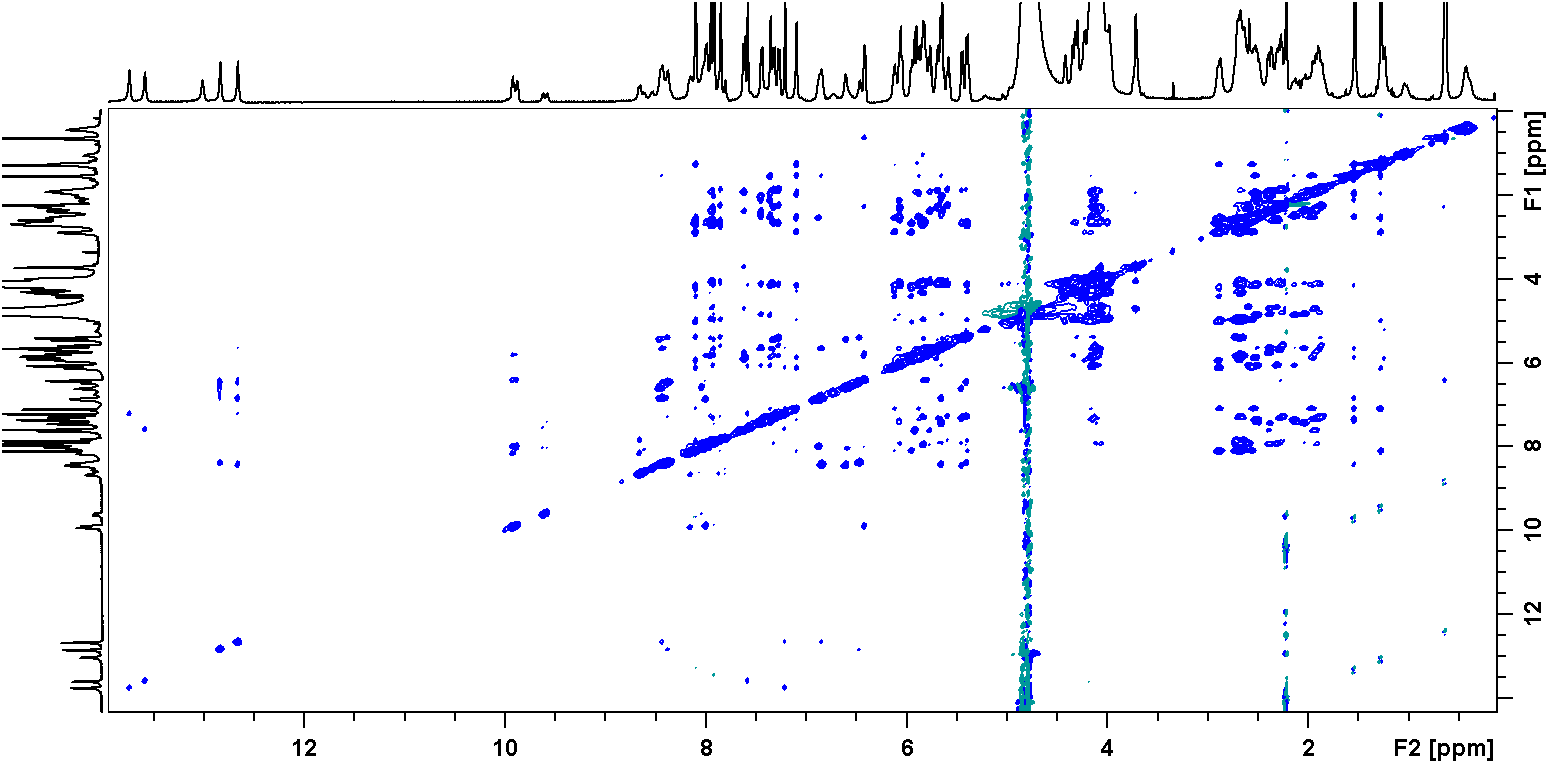


**Figure S36**: ^1^H–^1^H NOESY spectrum of d(5′-CGCGAATTGGCC-3′)_2_ upon addition of complex (**5a**) at r = 0.5 in H_2_O/ D_2_O 9:1 (buffer phosphate 100 mM, pH = 7.0) at 298 K, 500 MHz.


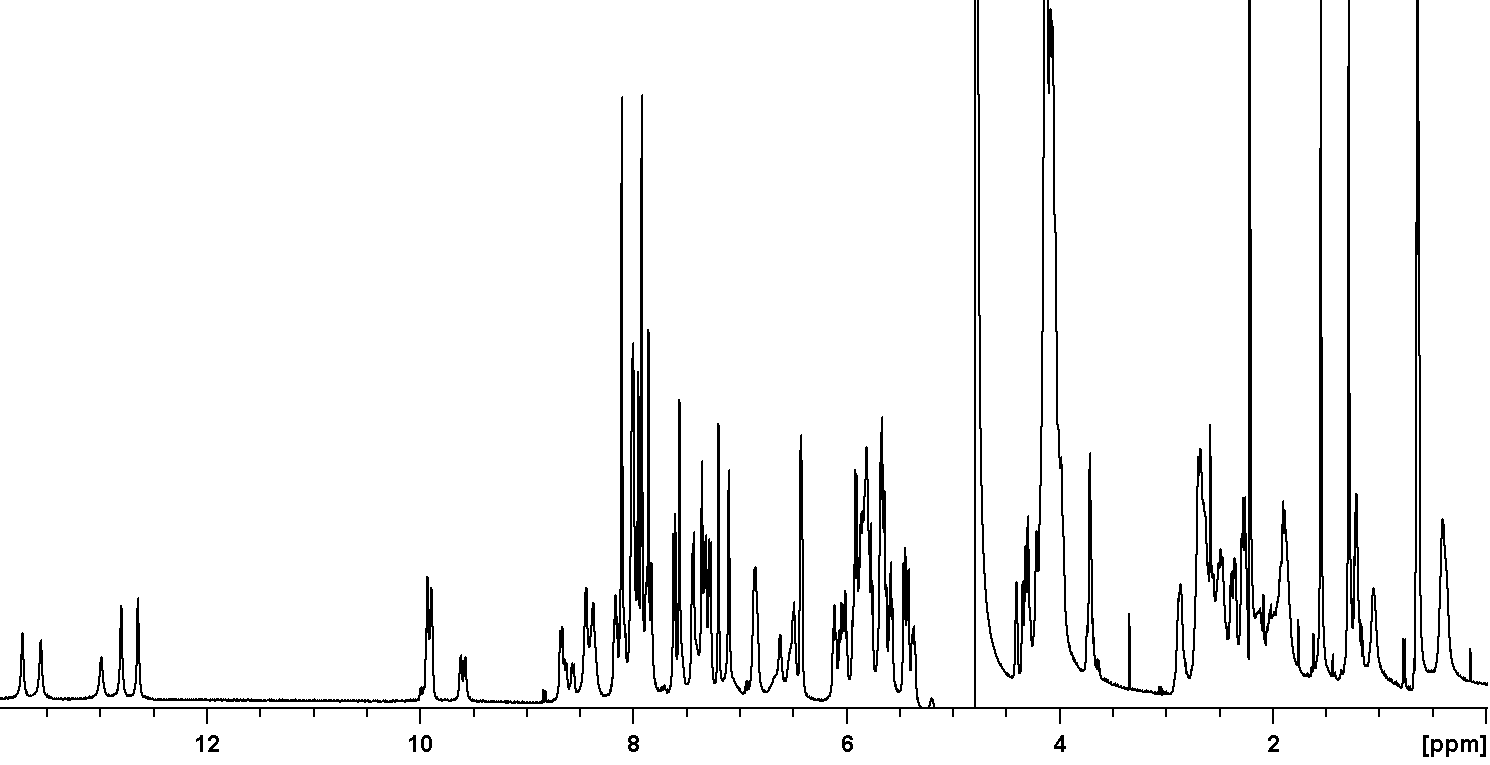


**Figure S37**: ^1^H NMR spectrum of d(5′-CGCGAATTGGCC-3′)_2_ upon addition of complex (**5a**) at r = 1 in H_2_O/ D_2_O 9:1 (buffer phosphate 100 mM, pH = 7.0) at 298 K, 500 MHz.


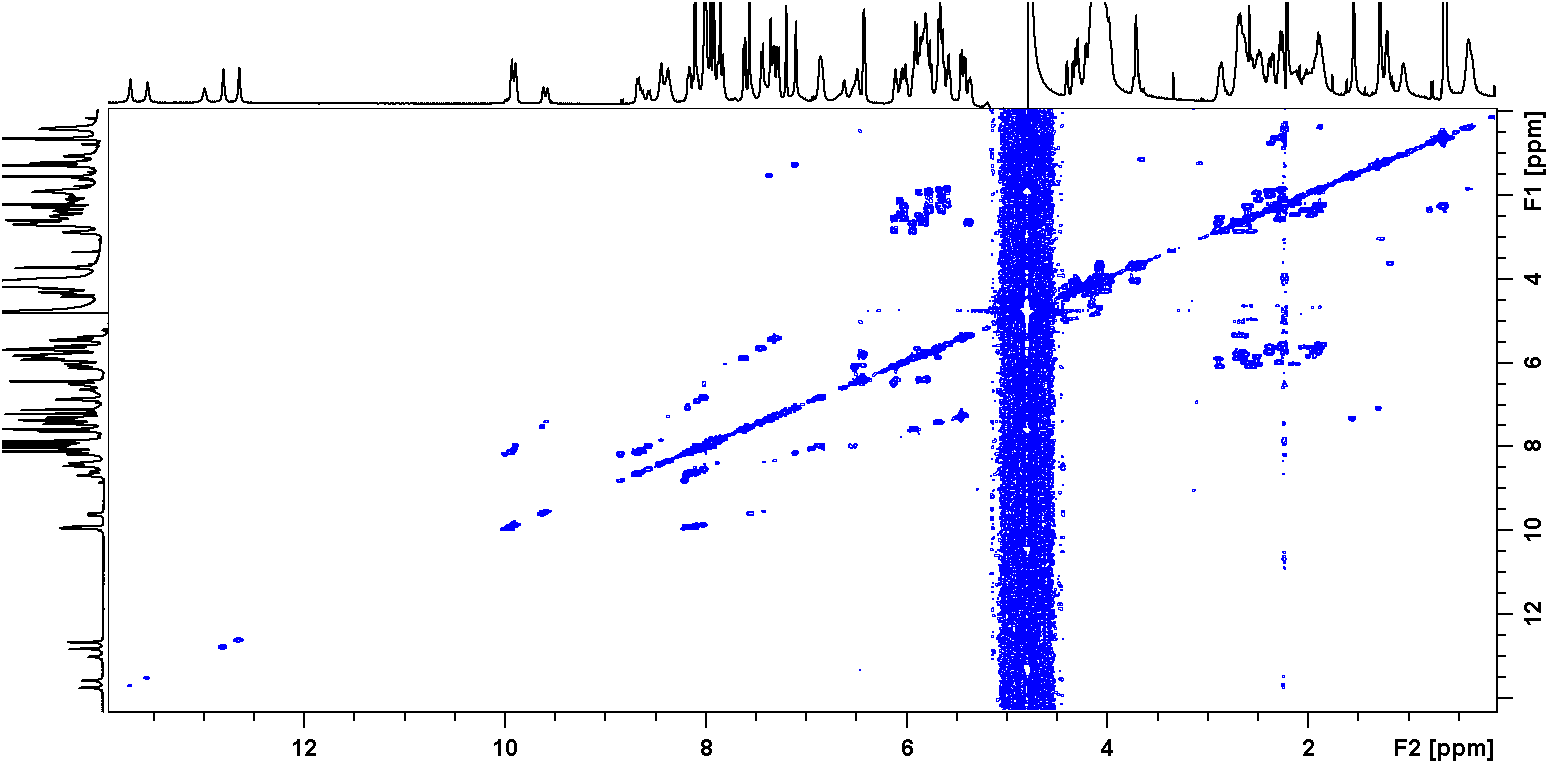


**Figure S38**: ^1^H–^1^H COSY spectrum of d(5′-CGCGAATTGGCC-3′)_2_ upon addition of complex (**5a**) at r = 1 in H_2_O/ D_2_O 9:1 (buffer phosphate 100 mM, pH = 7.0) at 298 K, 500 MHz.


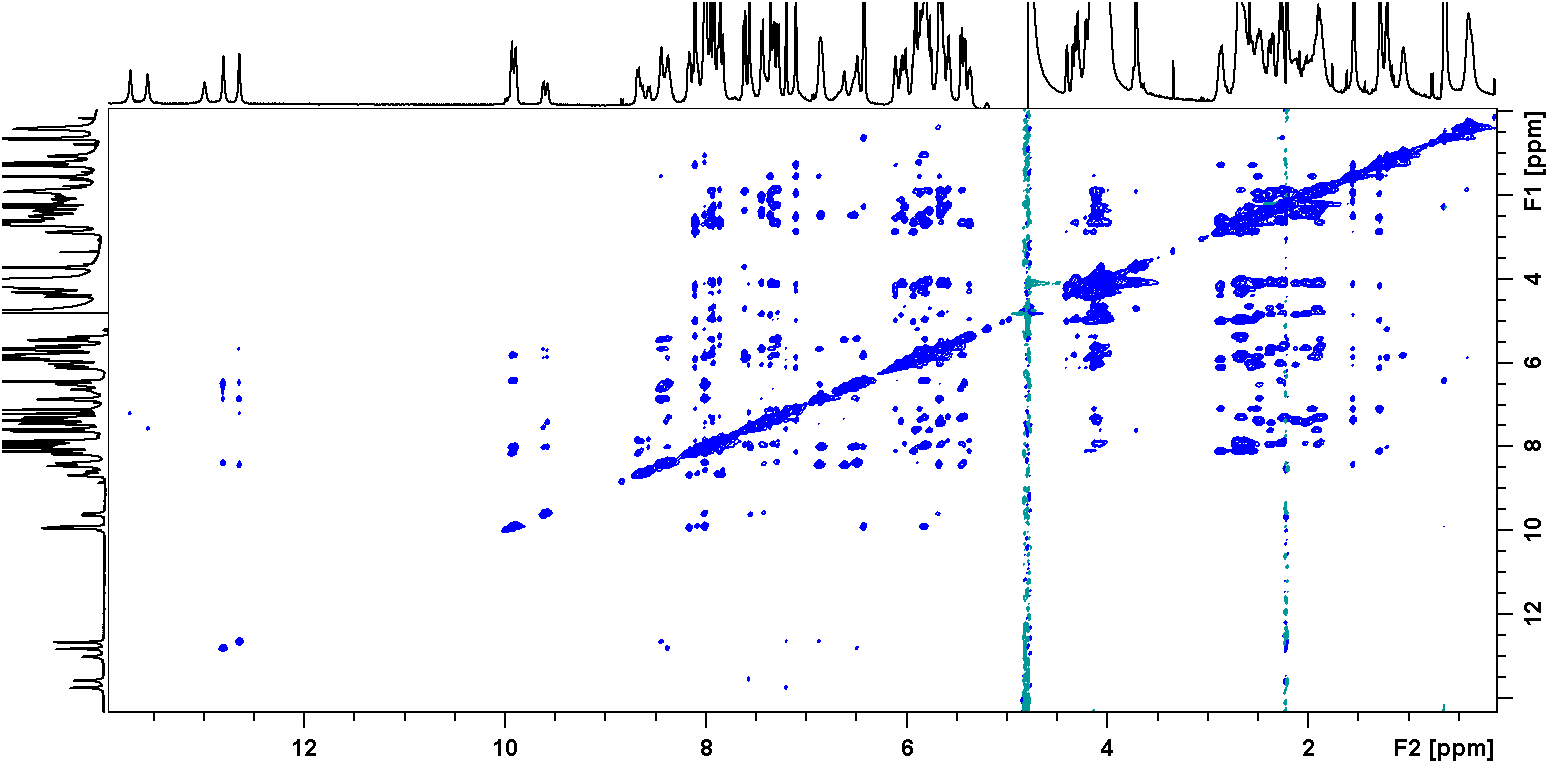


**Figure S39**: ^1^H–^1^H NOESY spectrum of d(5′-CGCGAATTGGCC-3′)_2_ upon addition of complex (**5a**) at r = 1 in H_2_O/ D_2_O 9:1 (buffer phosphate 100 mM, pH = 7.0) at 298 K, 500 MHz.


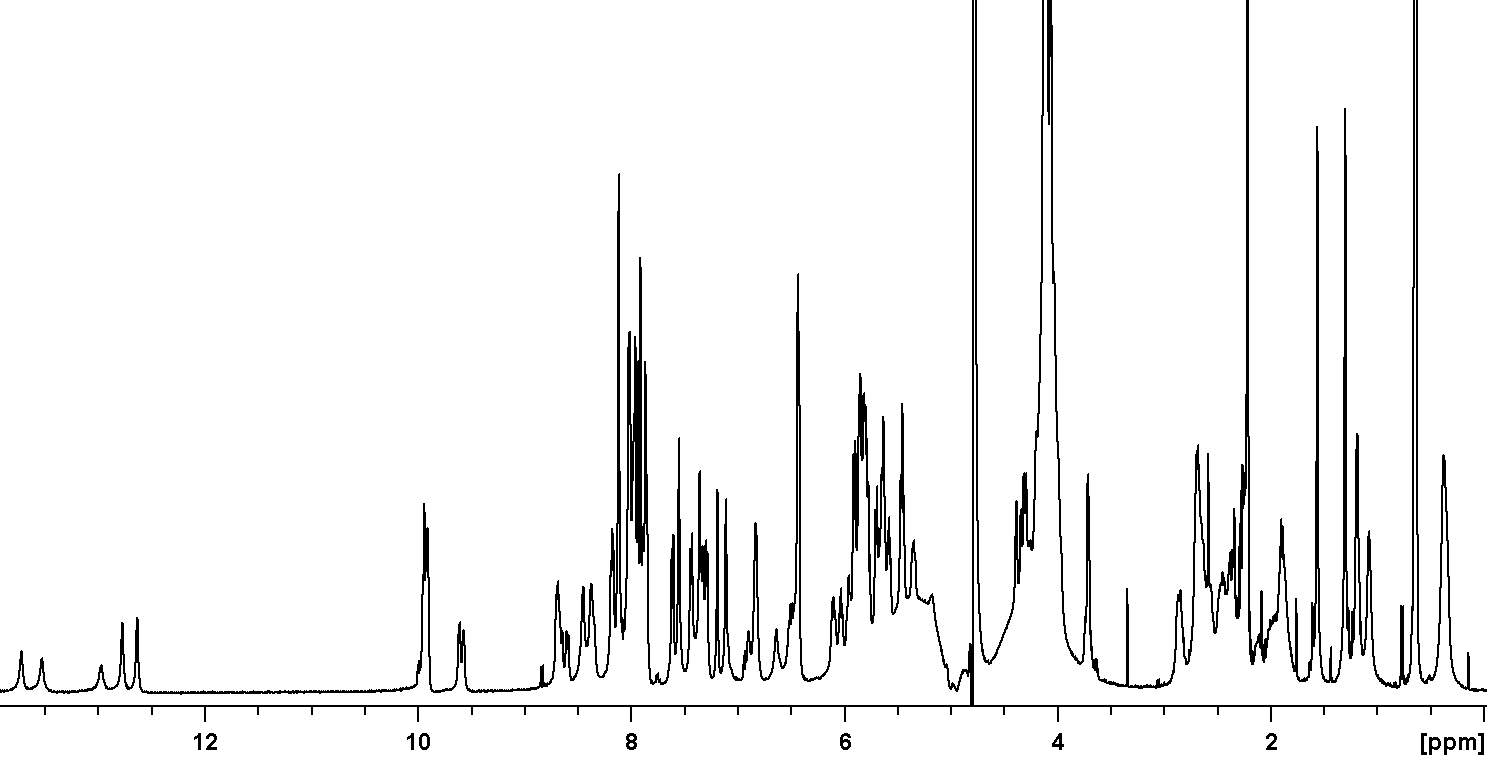


**Figure S40**: ^1^H NMR spectrum of d(5′-CGCGAATTGGCC-3′)_2_ upon addition of complex (**5a**) at r = 2 in H_2_O/ D_2_O 9:1 (buffer phosphate 100 mM, pH = 7.0) at 298 K, 500 MHz.


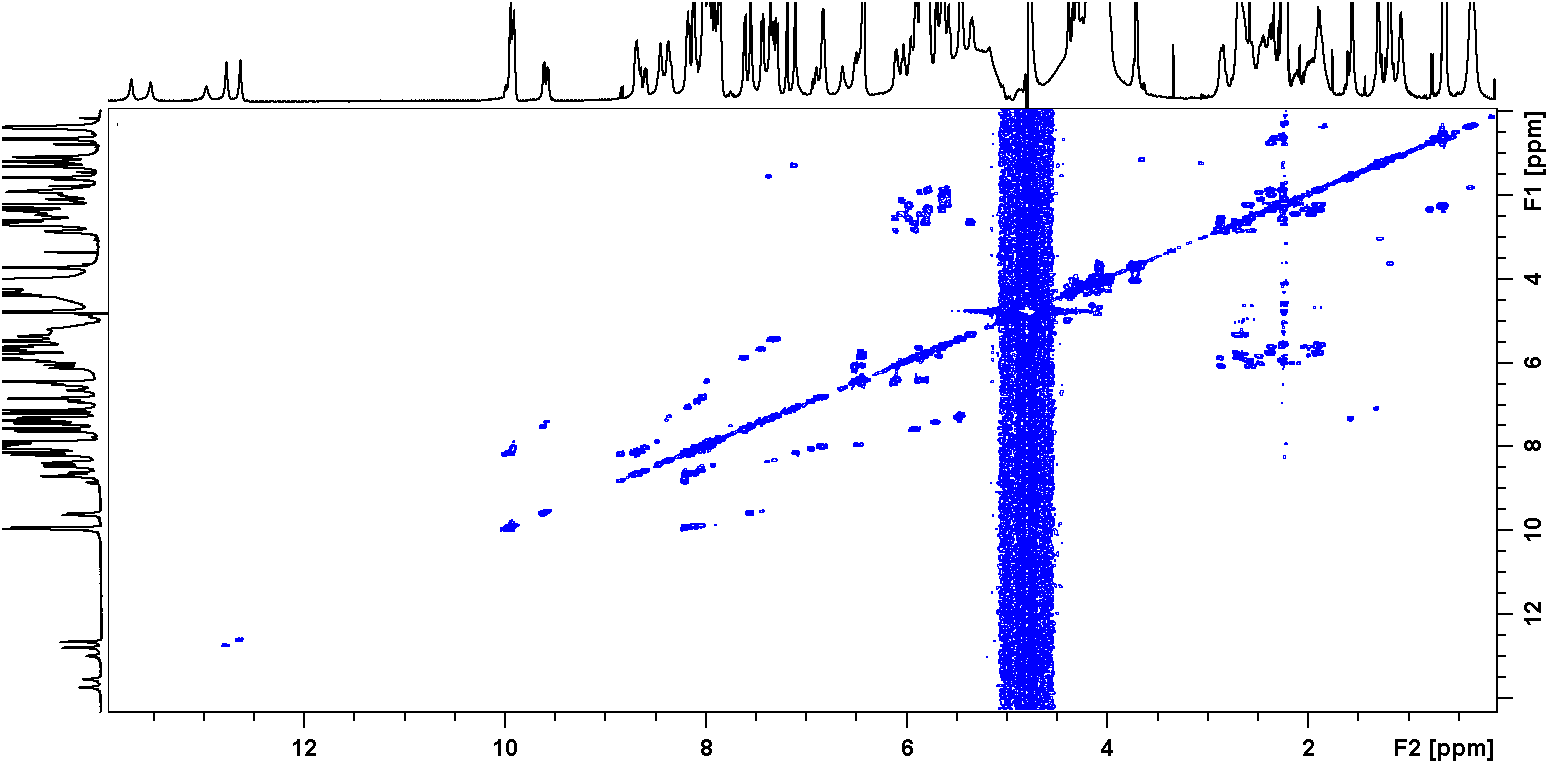


**Figure S41**: ^1^H–^1^H COSY spectrum of d(5′-CGCGAATTGGCC-3′)_2_ upon addition of complex (**5a**) at r = 2 in H_2_O/ D_2_O 9:1 (buffer phosphate 100 mM, pH = 7.0) at 298 K, 500 MHz.


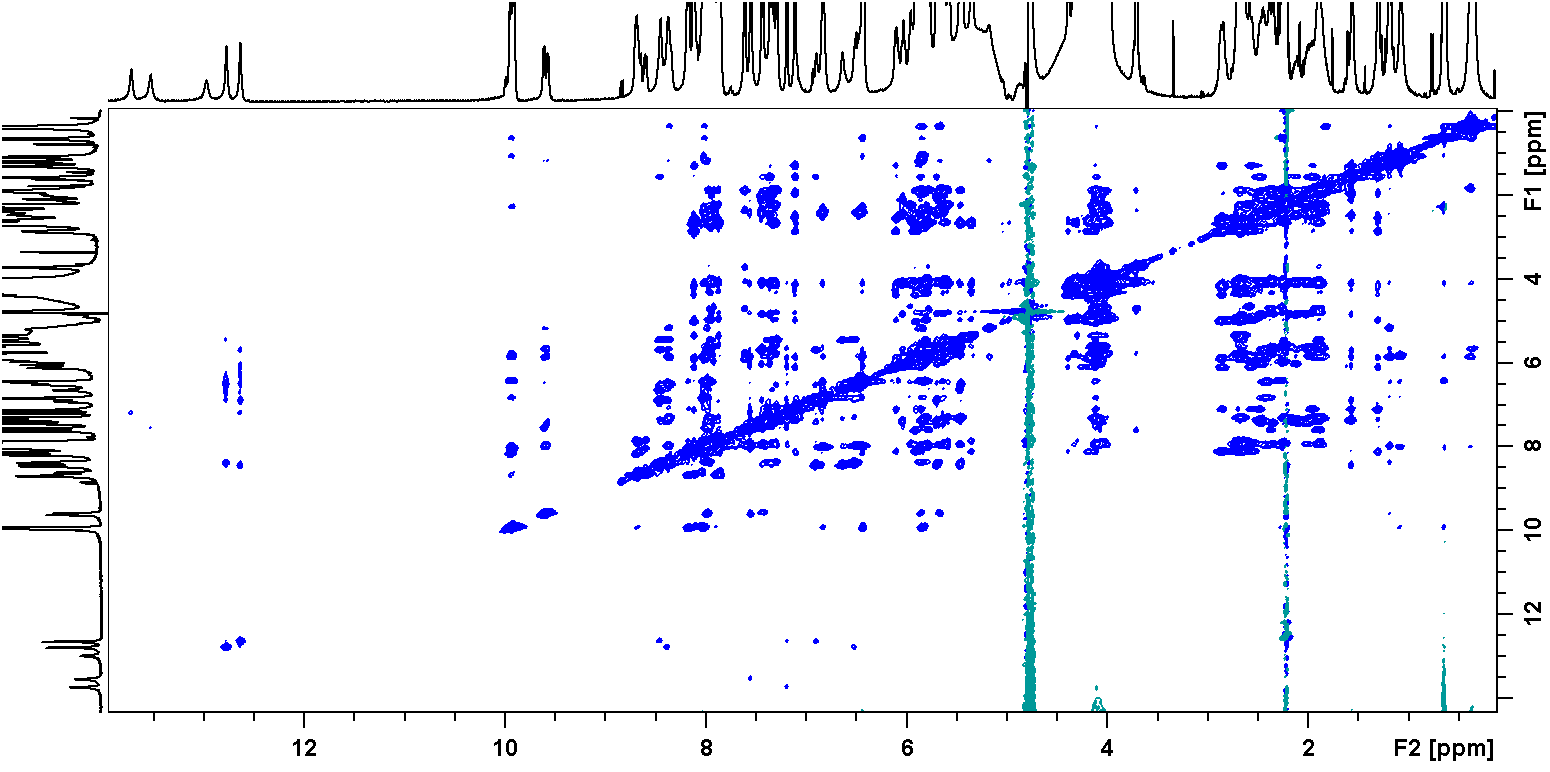


**Figure S42**: ^1^H–^1^H NOESY spectrum of d(5′-CGCGAATTGGCC-3′)_2_ upon addition of complex (**5a**) at r = 2 in H_2_O/ D_2_O 9:1 (buffer phosphate 100 mM, pH = 7.0) at 298 K, 500 MHz.


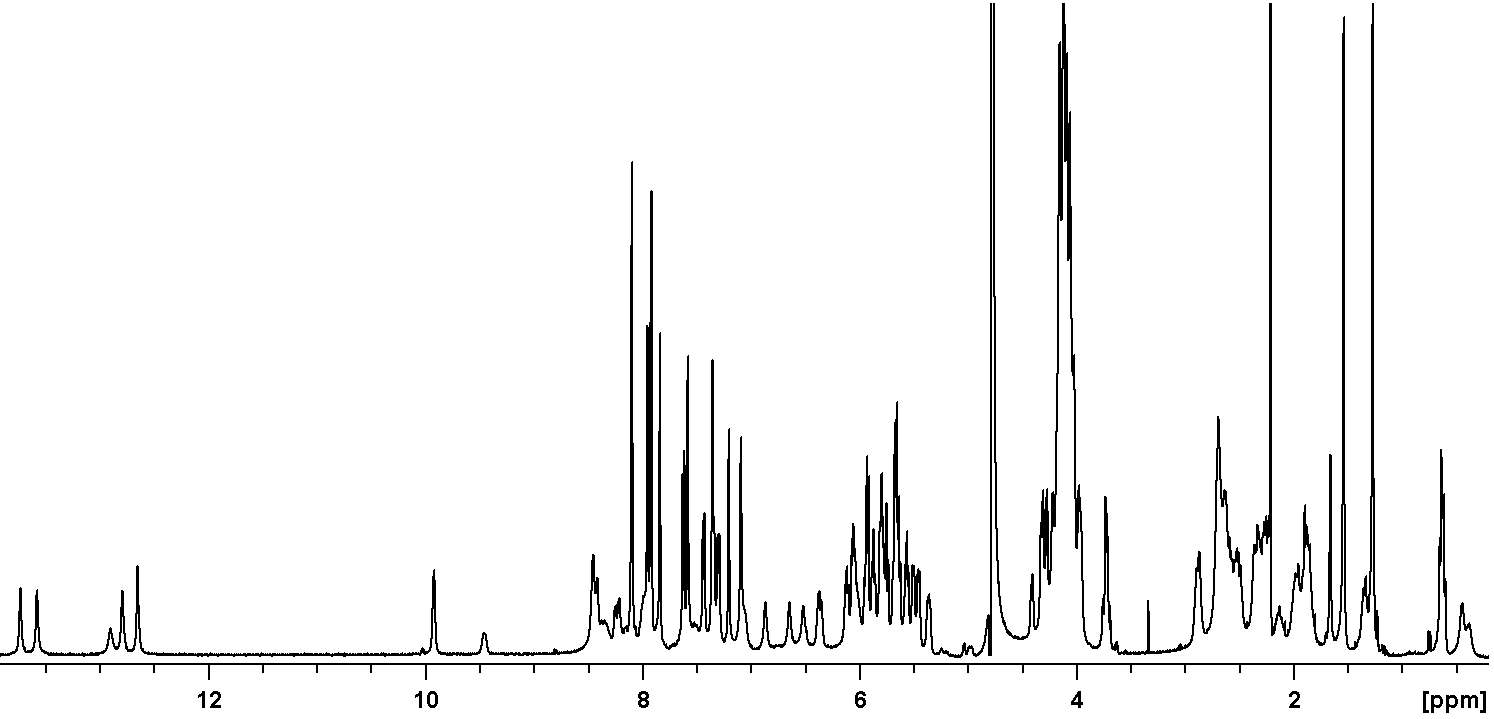


**Figure S43**: ^1^H NMR spectrum of d(5′-CGCGAATTGGCC-3′)_2_ upon addition of complex (**6a**) at r = 0.5 in H_2_O/ D_2_O 9:1 (buffer phosphate 100 mM, pH = 7.0) at 298 K, 500 MHz.


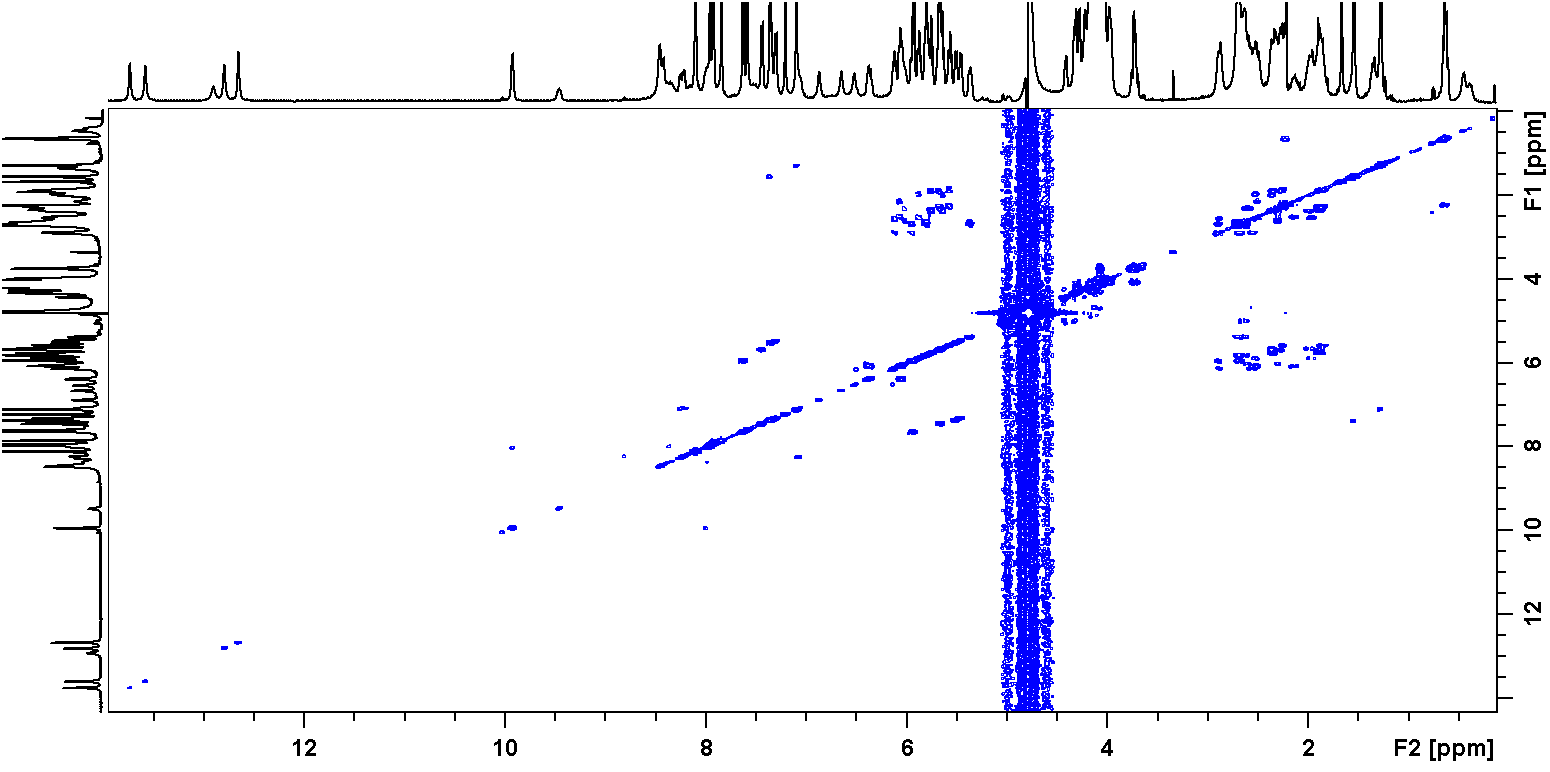


**Figure S44**: ^1^H–^1^H COSY spectrum of d(5′-CGCGAATTGGCC-3′)_2_ upon addition of complex (**6a**) at r = 0.5 in H_2_O/ D_2_O 9:1 (buffer phosphate 100 mM, pH = 7.0) at 298 K, 500 MHz.


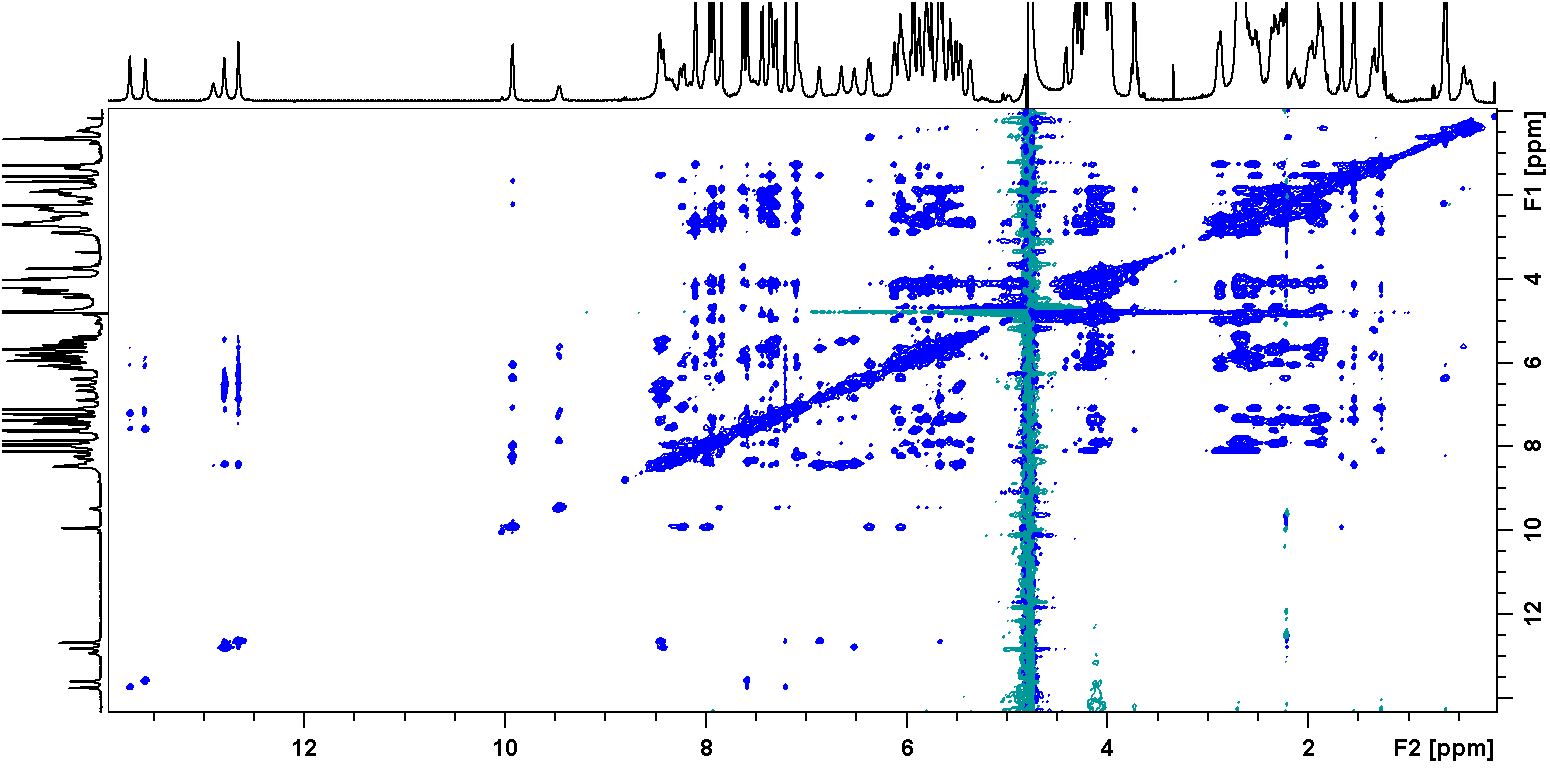


**Figure S45**: ^1^H–^1^H NOESY spectrum of d(5′-CGCGAATTGGCC-3′)_2_ upon addition of complex (**6a**) at r = 0.5 in H_2_O/ D_2_O 9:1 (buffer phosphate 100 mM, pH = 7.0) at 298 K, 500 MHz.


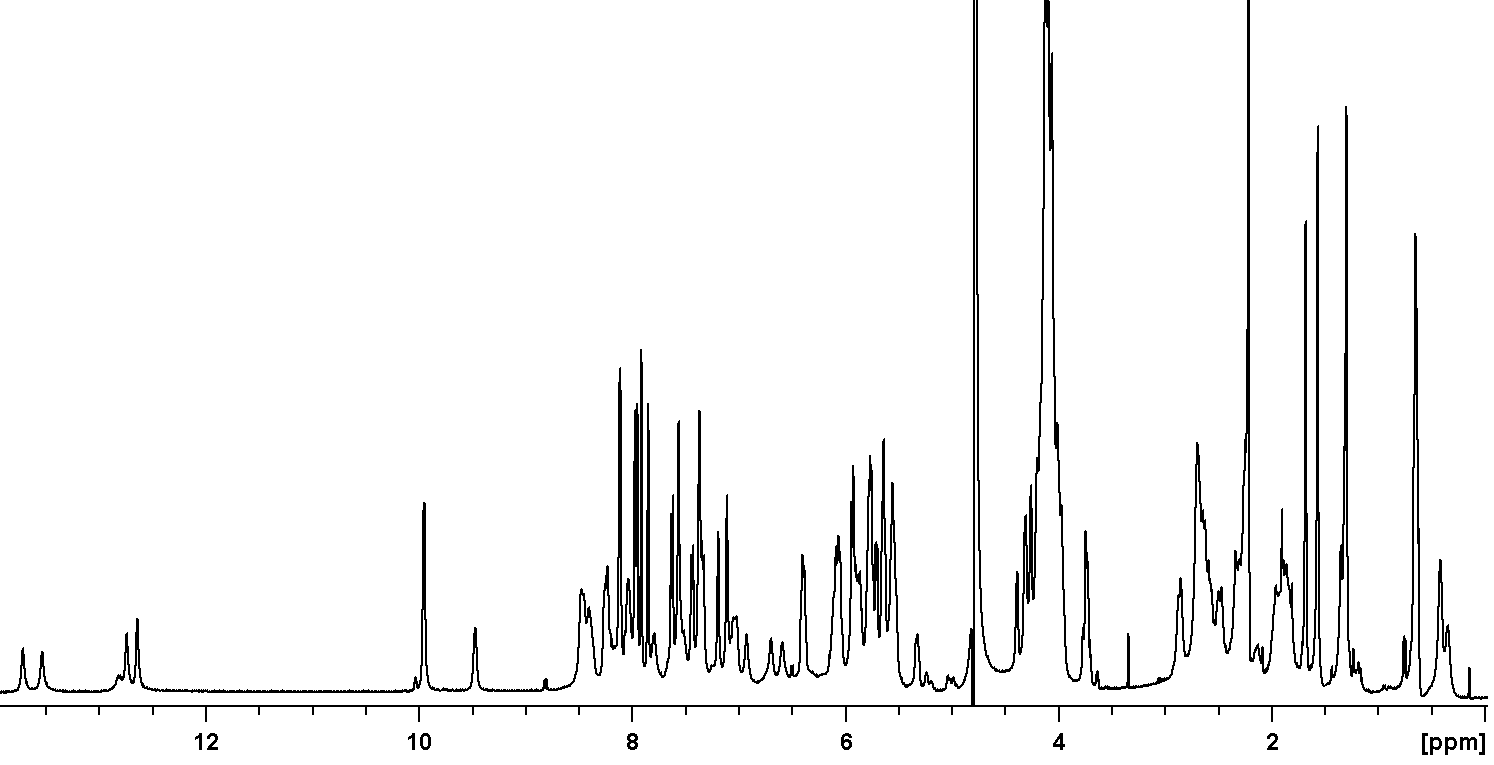


**Figure S46**: ^1^H NMR spectrum of d(5′-CGCGAATTGGCC-3′)_2_ upon addition of complex (**6a**) at r = 1 in H_2_O/ D_2_O 9:1 (buffer phosphate 100 mM, pH = 7.0) at 298 K, 500 MHz.


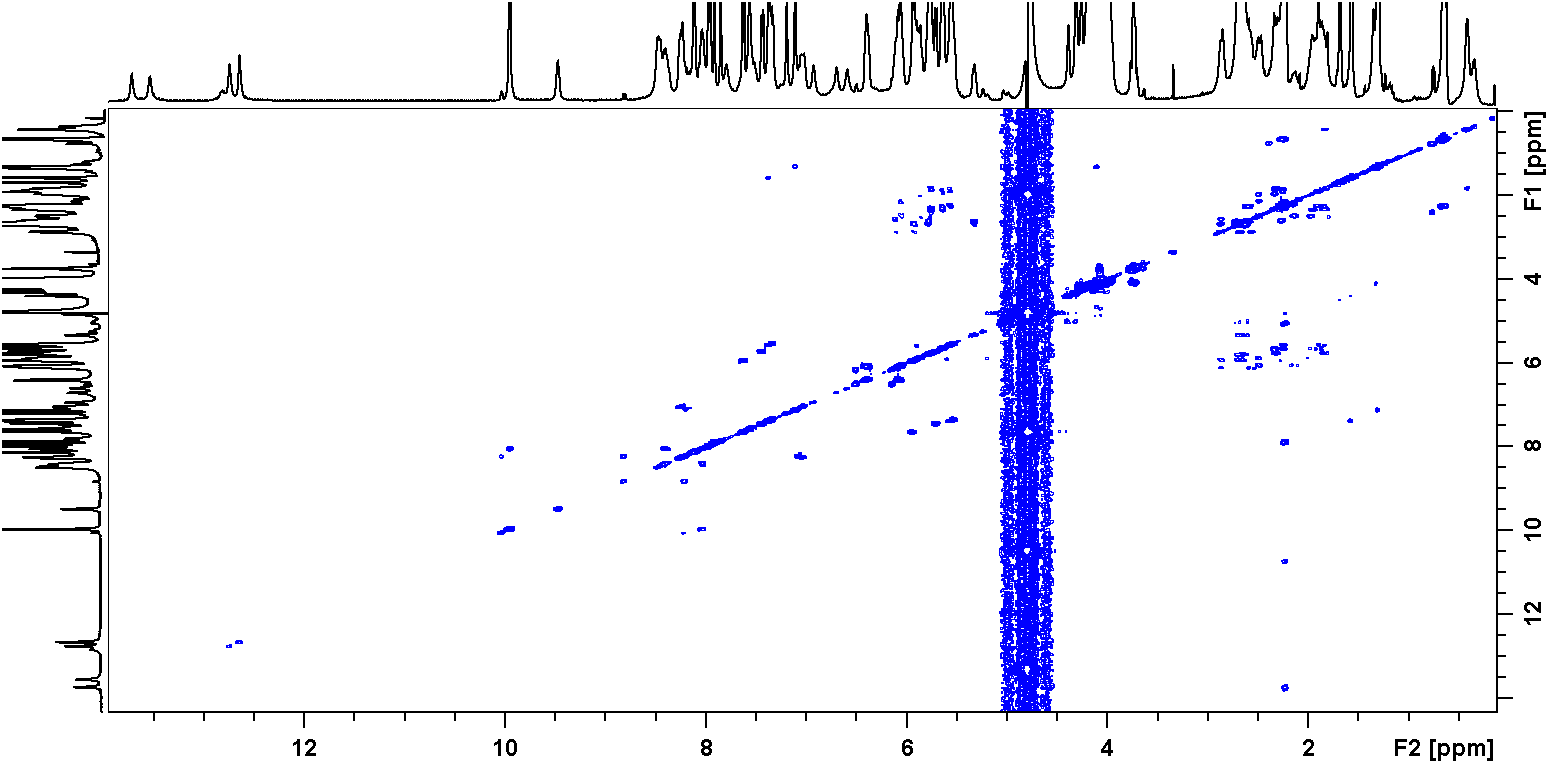


**Figure S47**: ^1^H–^1^H COSY spectrum of d(5′-CGCGAATTGGCC-3′)_2_ upon addition of complex (**6a**) at r = 1 in H_2_O/ D_2_O 9:1 (buffer phosphate 100 mM, pH = 7.0) at 298 K, 500 MHz.


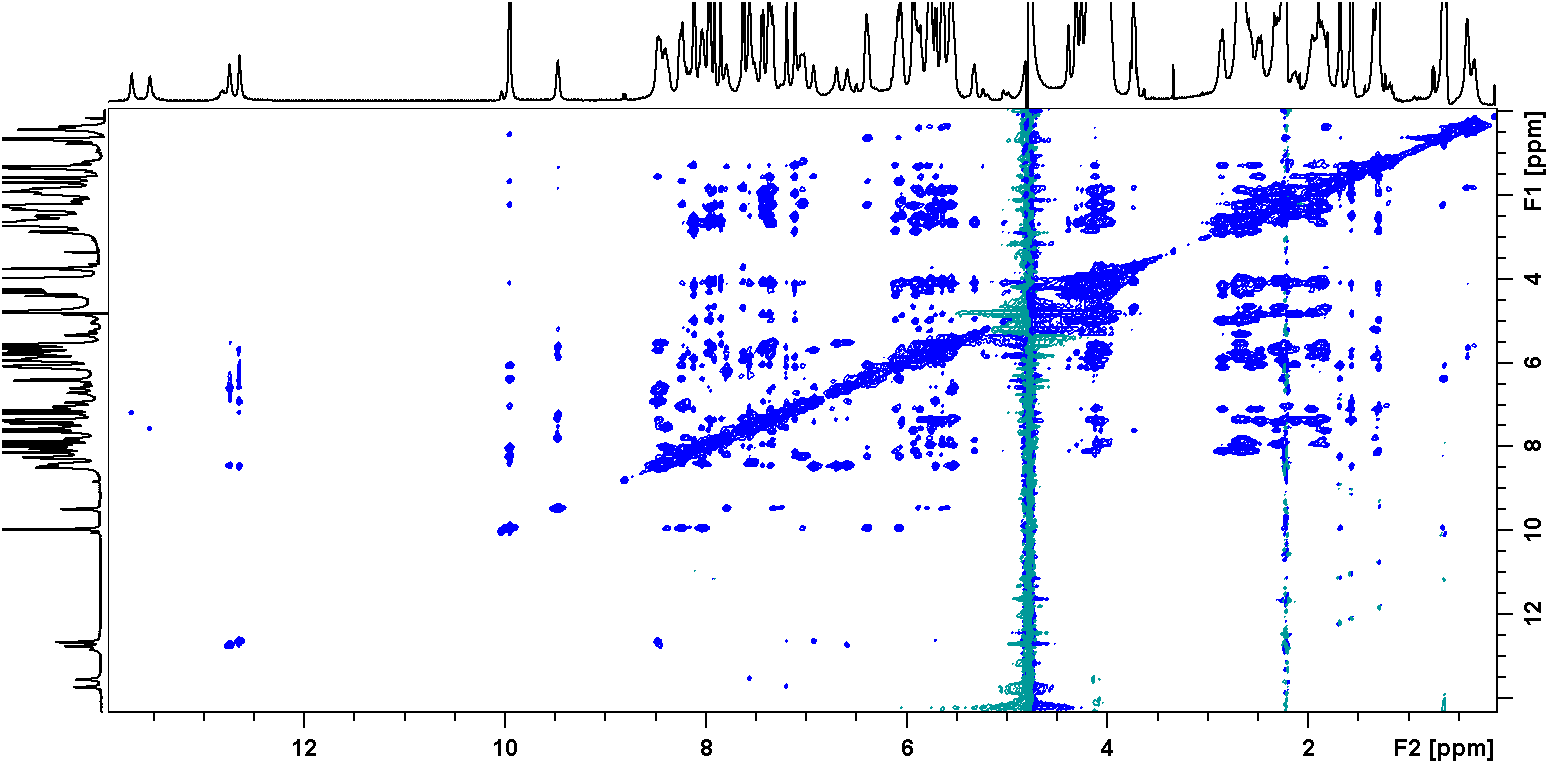


**Figure S48**: ^1^H–^1^H NOESY spectrum of d(5′-CGCGAATTGGCC-3′)_2_ upon addition of complex (**6a**) at r = 1 in H_2_O/ D_2_O 9:1 (buffer phosphate 100 mM, pH = 7.0) at 298 K, 500 MHz.


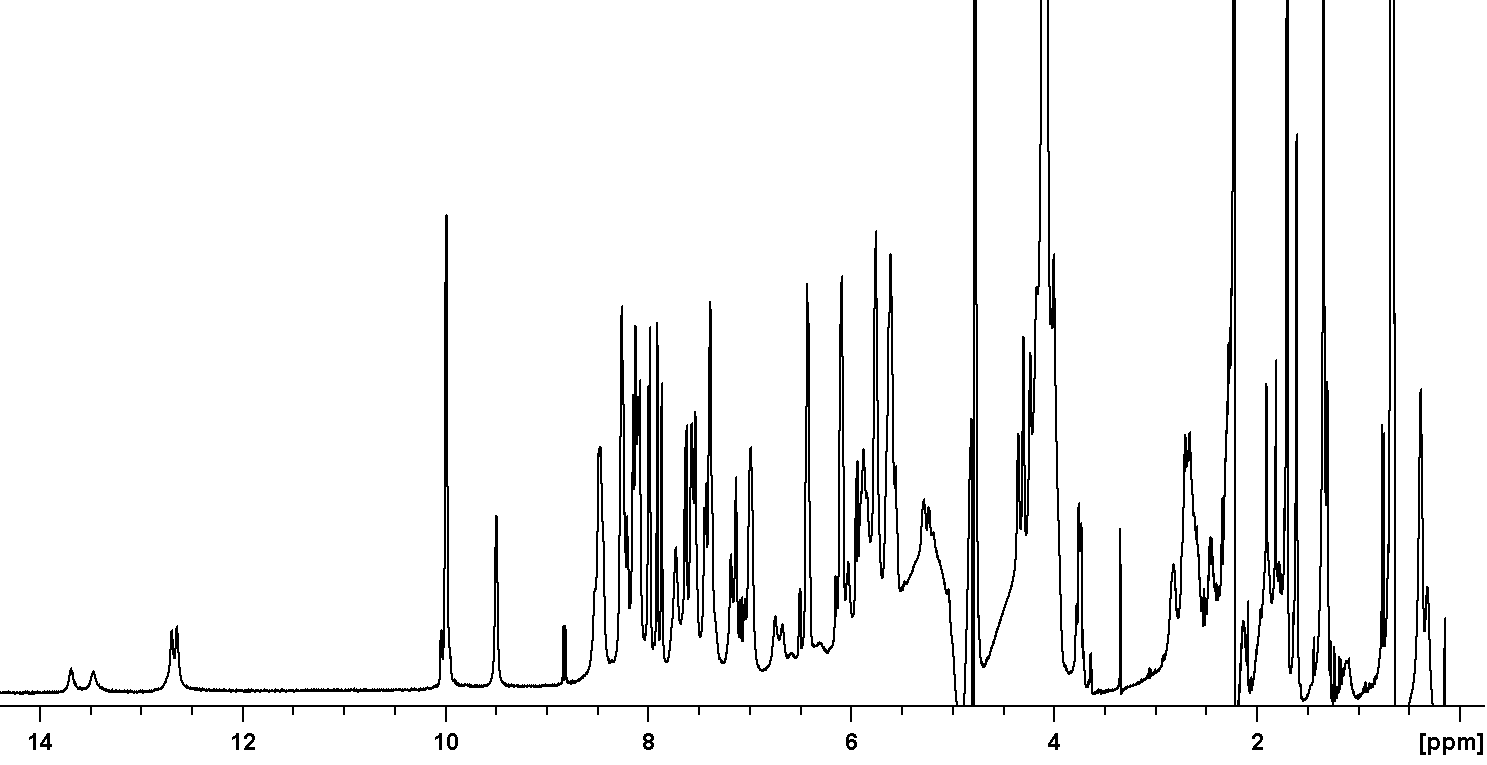


**Figure S49**: ^1^H NMR spectrum of d(5′-CGCGAATTGGCC-3′)_2_ upon addition of complex (**6a**) at r = 2 in H_2_O/ D_2_O 9:1 (buffer phosphate 100 mM, pH = 7.0) at 298 K, 500 MHz.


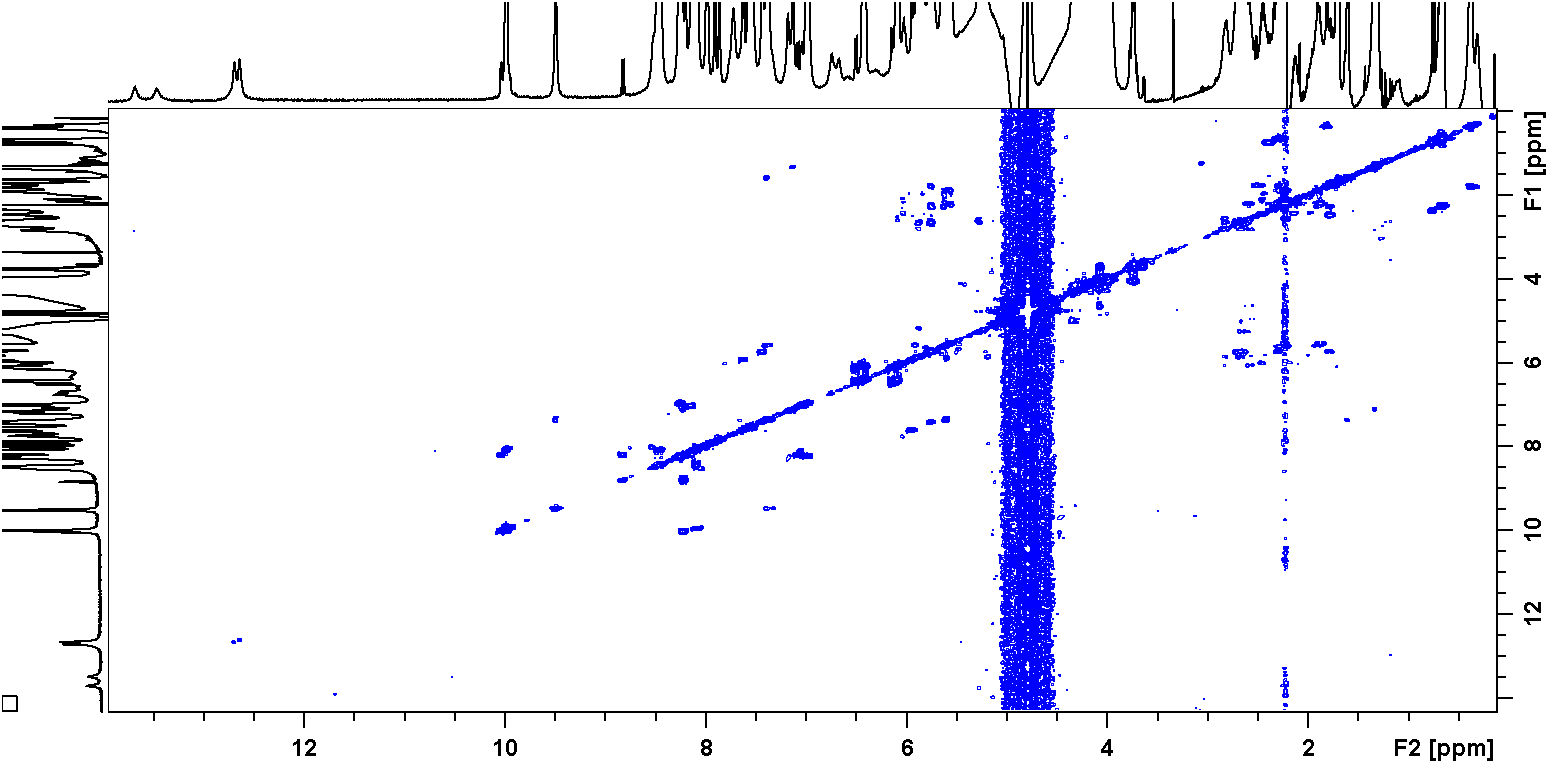


**Figure S50**: ^1^H–^1^H COSY spectrum of d(5′-CGCGAATTGGCC-3′)_2_ upon addition of complex (**6a**) at r = 2 in H_2_O/ D_2_O 9:1 (buffer phosphate 100 mM, pH = 7.0) at 298 K, 500 MHz.


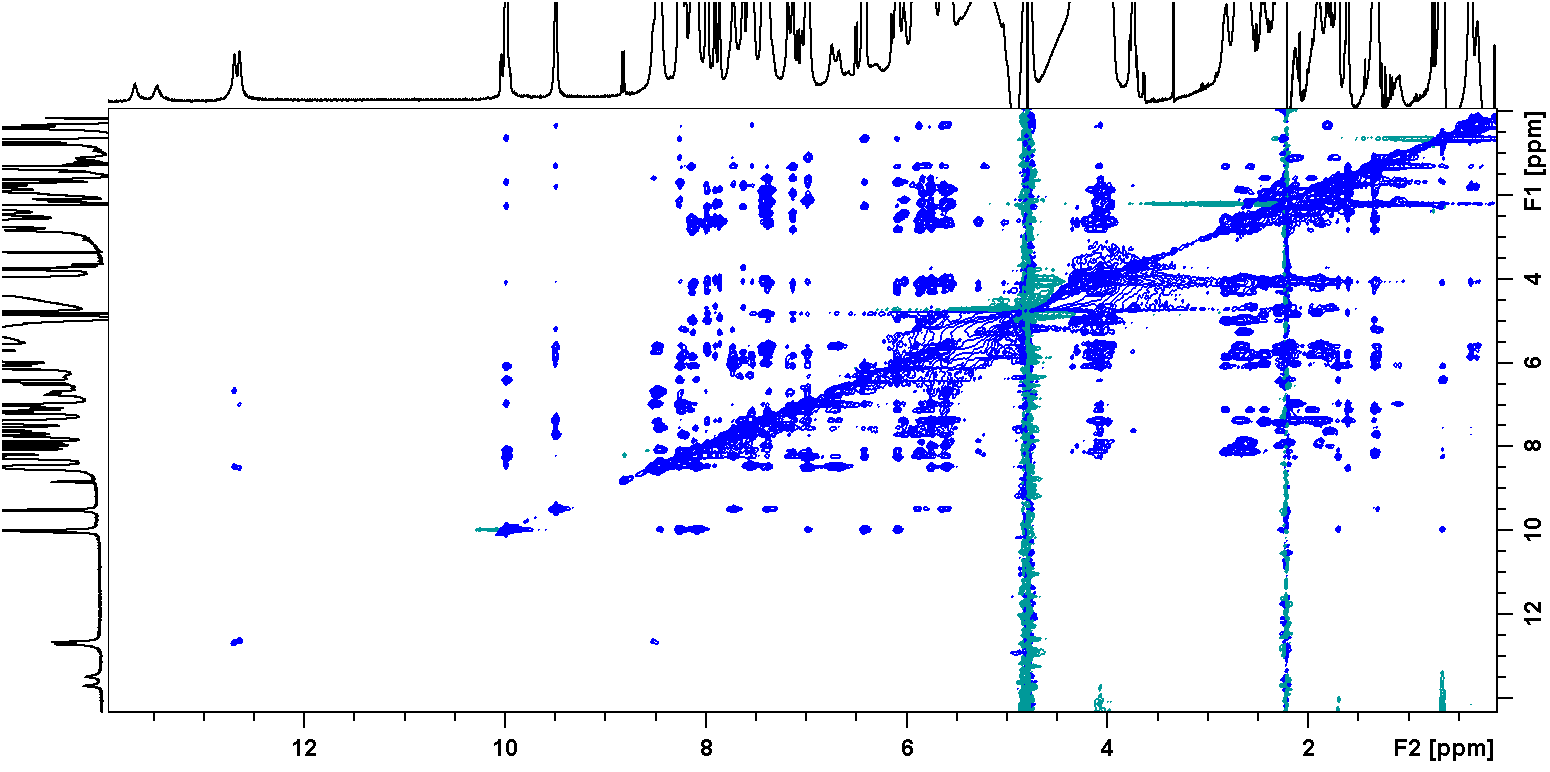


**Figure S51**: ^1^H–^1^H NOESY spectrum of d(5′-CGCGAATTGGCC-3′)_2_ upon addition of complex (**6a**) at r = 2 in H_2_O/ D_2_O 9:1 (buffer phosphate 100 mM, pH = 7.0) at 298 K, 500 MHz.

**
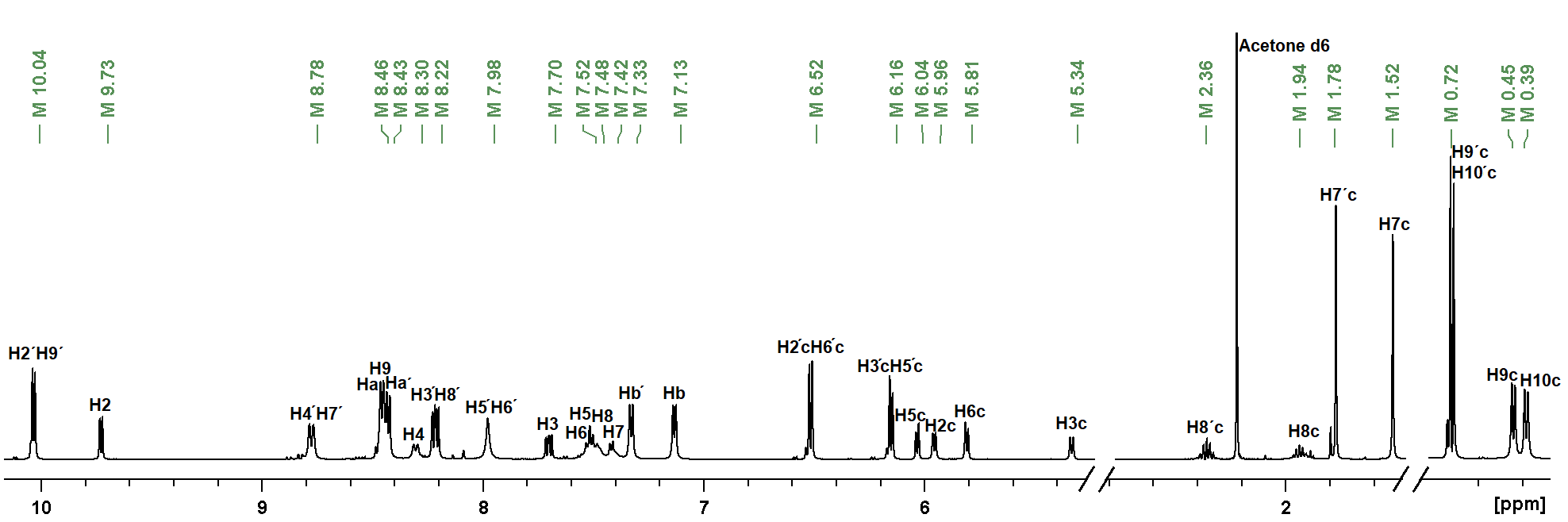
**

**Figure S52**: ^1^H NMR spectrum of the complex (**4a**) in D_2_O at 298 K.


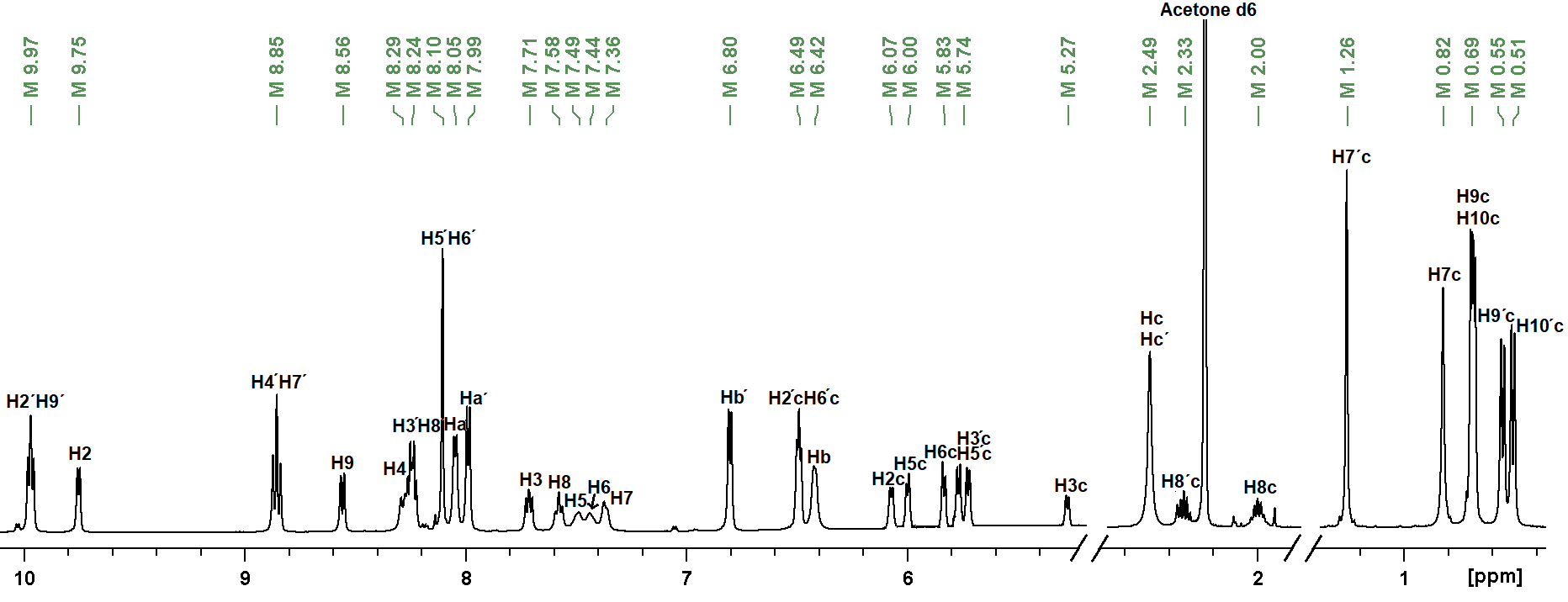


**Figure S53**: ^1^H NMR spectrum of the complex (**5a**) in D_2_O at 298 K.

**
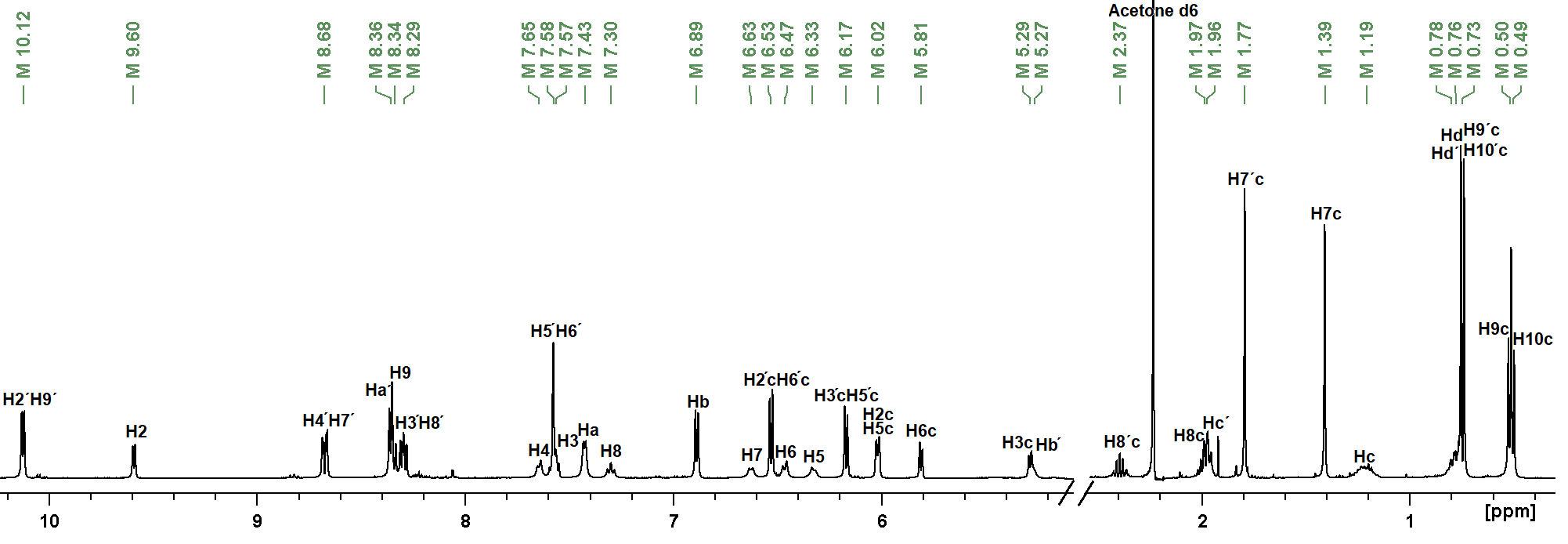
**

**Figure S54**: ^1^H NMR spectrum of the complex (**6a**) in D_2_O at 298 K.

**
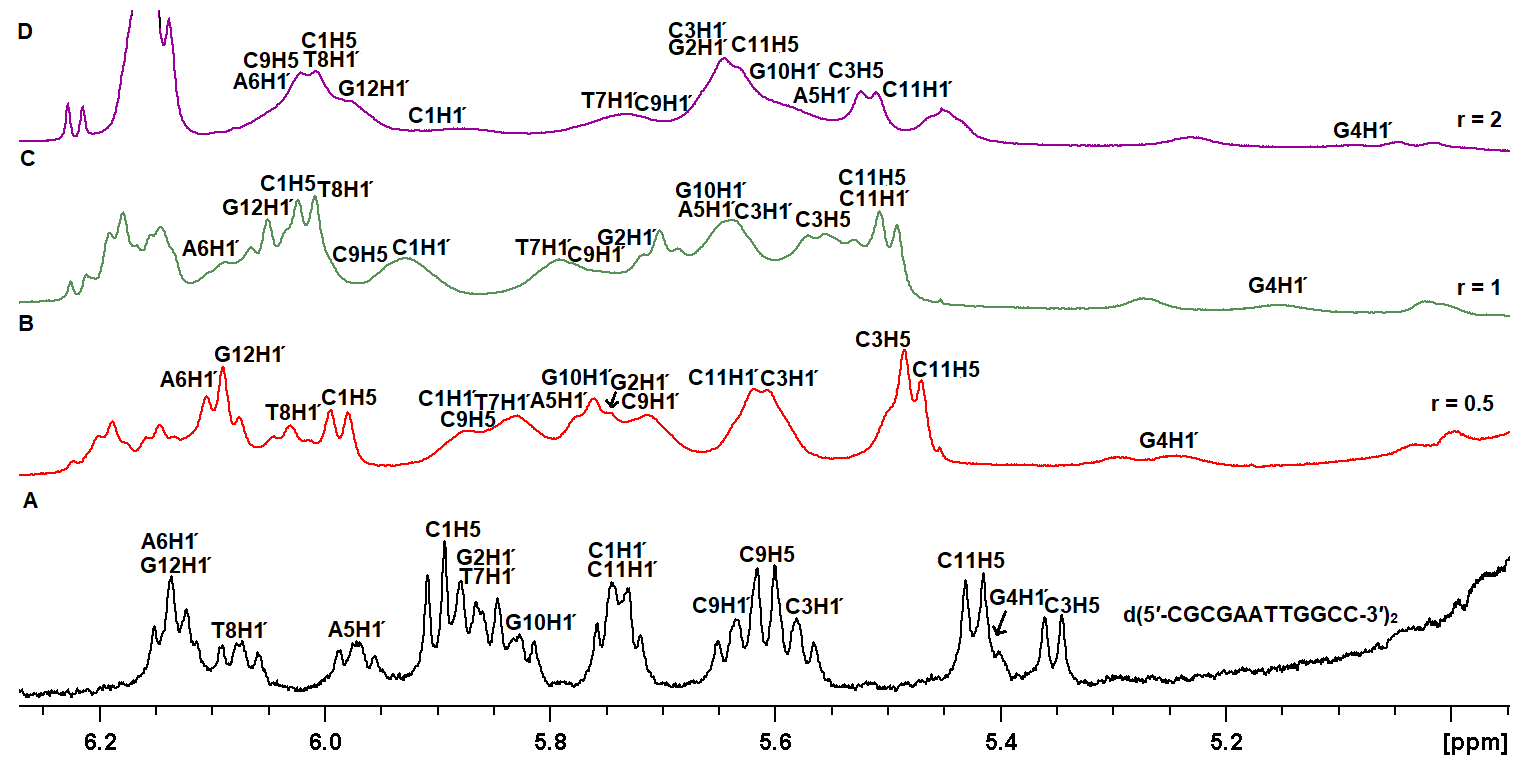
**

**Figure S55**: H5 and H1΄region of ¹H NMR spectra (H_2_O:D_2_O, 9 :1, 298 K, phosphate buffer 100 mM, pH = 7.0) with proton assignments, recorded during the titration of d(5′-CGCGAATTGGCC-3′)_2_ with complex (**4a**) at three different ratios: (A) free oligonucleotide, (B) r = 0.5, (C) r = 1, and (D) r = 2.

**
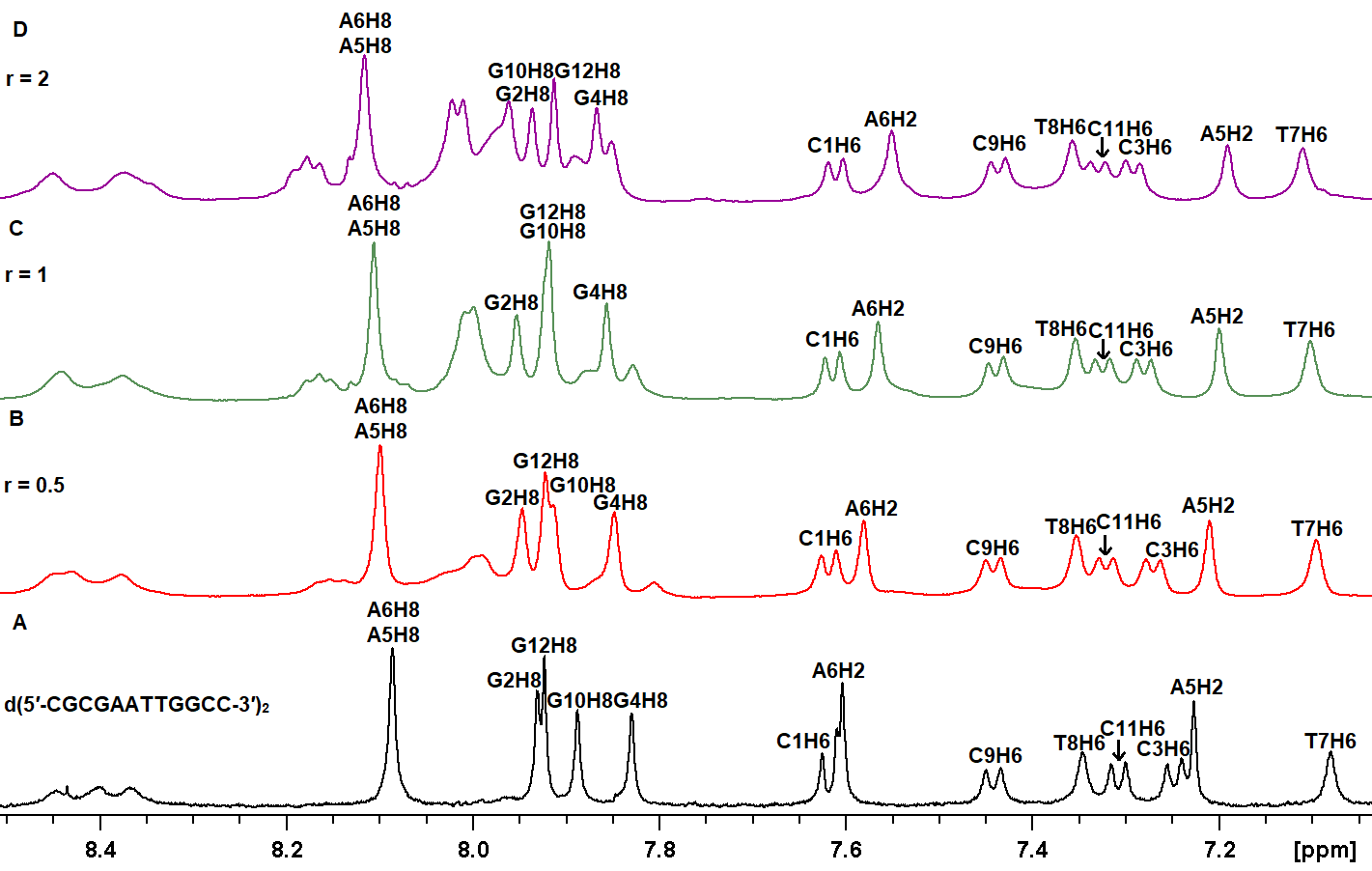
**

**Figure S56**: Part of ¹H NMR spectra (H_2_O:D_2_O, 9 :1, 298 K, phosphate buffer 100 mM, pH = 7.0) with proton assignments, recorded during the titration of d(5′-CGCGAATTGGCC-3′)_2_ with complex (**5a**) at three different ratios: (A) free oligonucleotide, (B) r = 0.5, (C) r = 1, and (D) r = 2.

**
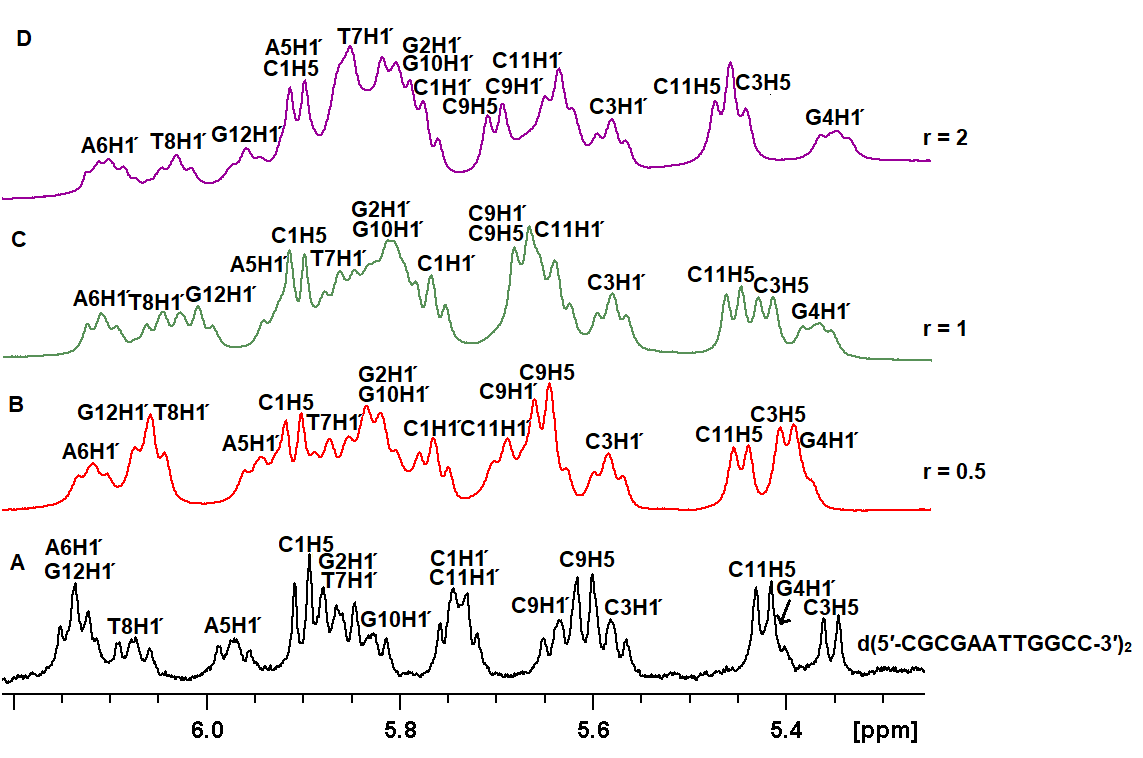
**

**Figure S57**: H5 and H1΄region of ¹H NMR spectra (H_2_O:D_2_O, 9 :1, 298 K, phosphate buffer 100 mM, pH = 7.0) with proton assignments, recorded during the titration of d(5′-CGCGAATTGGCC-3′)_2_ with complex (**5a**) at three different ratios: (A) free oligonucleotide, (B) r = 0.5, (C) r = 1, and (D) r = 2.

**
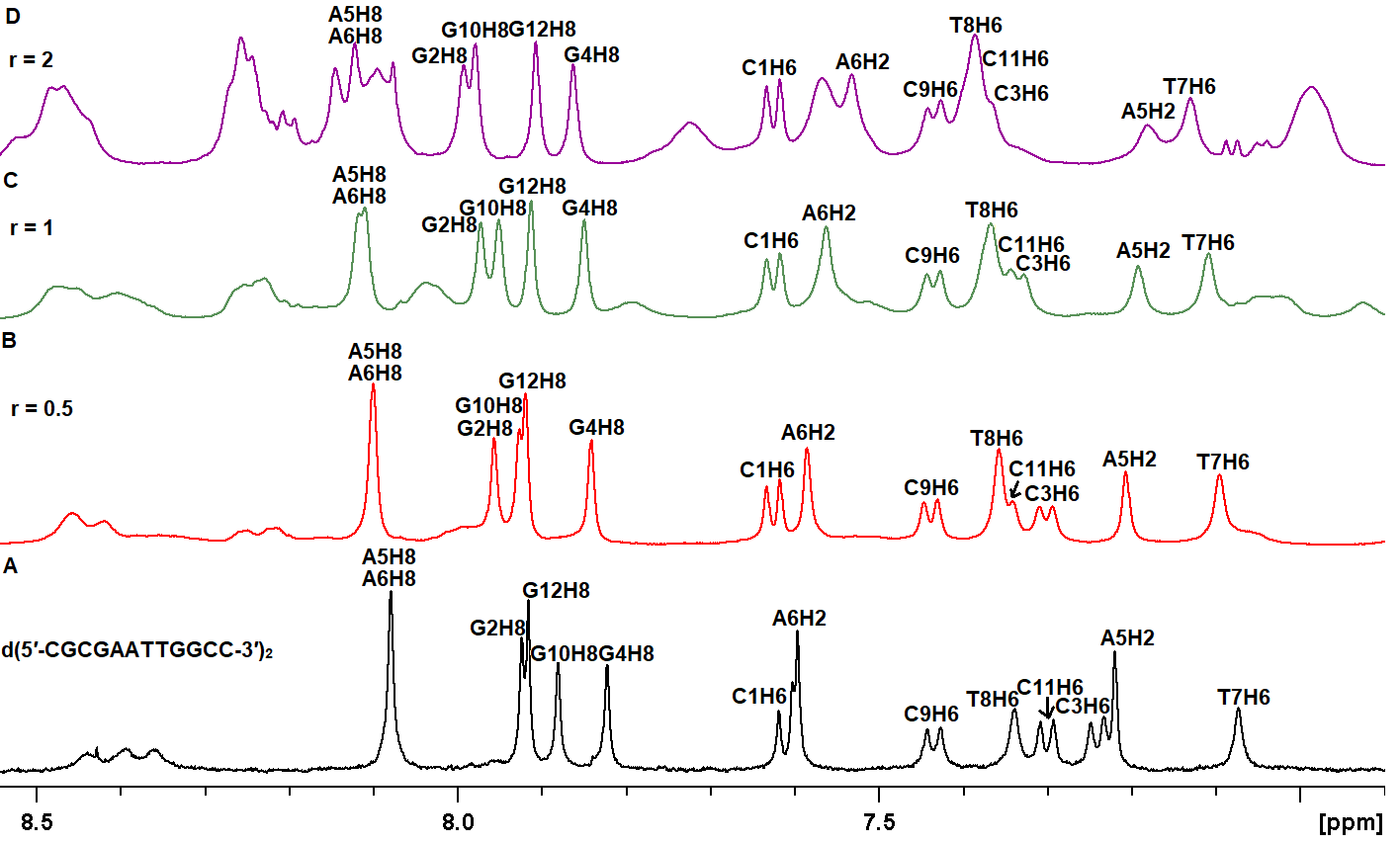
**

**Figure S58**: Part of ¹H NMR spectra (H_2_O:D_2_O, 9 :1, 298 K, phosphate buffer 100 mM, pH = 7.0) with proton assignments, recorded during the titration of d(5′-CGCGAATTGGCC-3′)_2_ with complex (**6a**) at three different ratios: (A) free oligonucleotide, (B) r = 0.5, (C) r = 1, and (D) r = 2.

**
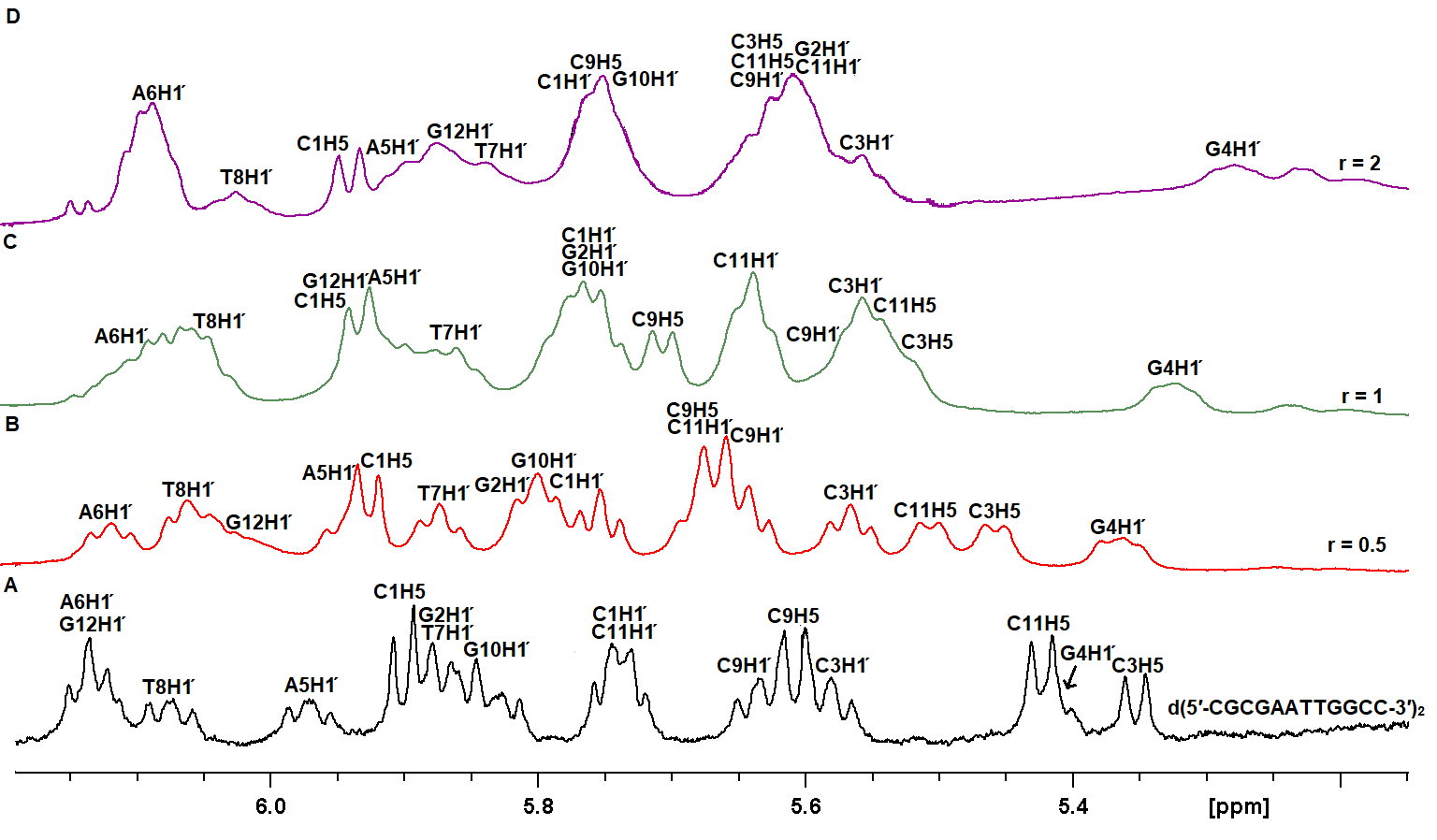
**

**Figure S59**: H5 and H1΄region of ¹H NMR spectra (H_2_O:D_2_O, 9 :1, 298 K, phosphate buffer 100 mM, pH = 7.0) with proton assignments, recorded during the titration of d(5′-CGCGAATTGGCC-3′)_2_ with complex (**6a**) at three different ratios: (A) free oligonucleotide, (B) r = 0.5, (C) r = 1, and (D) r = 2.

**Table S4** ^1^H NMR chemical shifts of the (**4a**) (H_2_O : D_2_O, 9 : 1, 298 K, buffer phosphates 100 mM, pH = 7.0) free, and upon the addition to the d(5′-CGCGAATTGGCC-3′)_2_ at r = 0.5, 1, and 2. Shifts are denoting in parenthesis (negative sign upfield and positive sign downfield shifts). A, B and C denotes the signals of the three moieties of (**4a**). n.o. = not observed.

|  |  |  | **r = 0.5** | | | **r = 1** | | | **r = 2** | | |
| --- | --- | --- | --- | --- | --- | --- | --- | --- | --- | --- | --- |
| Ligands | Protons | **4a** | A | B | C | A | B | C | A | B | C |
| phe | H_2΄/9΄_ | 10.04 | 9.94  (-0.10) | 9.72  (-0.32) | 9.65  (-0.39) | 9.96  (-0.08) | 9.78  (-0.26) | 9.69  (-0.35) | 9.99  (-0.05) | 9.92  (-0.12) | 9.82  (-0.22) |
|  | H_3΄/8΄_ | 8.22 | 8.05  (-0.17) | n.o. | 7.58  (-0.64) | 8.08  (-0.14) | - | - | 8.12  (-0.10) | - | - |
|  | H_4΄/7΄_ | 8.78 | 8.34  (-0.44) | - |  | 8.34  (-0.44) | - | - | 8.39  (-0.39) | - | - |
|  | H_5΄/6΄_ | 7.98 | n.o. | n.o. | n.o. | n.o. | n.o. | n.o. | n.o. | n.o. | n.o. |
| bq | H_2_ | 9.73 | 9.59  (-0.14) | 9.25  (-0.48) | 9.60  (-0.13) | 9.59  (-0.14) | 9.28  (-0.45) | - | 9.61  (-0.12) | 9.40  (-0.33) | - |
|  | H_3_ | 7.70 | 7.49  (-0.21) | - | - | 7.51  (-0.19) | - | - | 7.56  (-0.14) | - | - |
|  | H_4_ | 8.30 | 7.80  (-0.50) | 8.12  (-0.18) | - | 7.88  (-0.42) | - | - | 7.99  (-0.31) | - | - |
|  | H_5_ | 7.51 | n.o. | n.o. | n.o. | n.o. | n.o. | n.o. | n.o. | n.o. | n.o. |
|  | H_6_ | 7.53 | n.o. | n.o. | n.o. | n.o. | n.o. | n.o. | n.o. | n.o. | n.o. |
|  | H_7_ | 7.42 | n.o. | n.o. | n.o. | n.o. | n.o. | n.o. | n.o. | n.o. | n.o. |
|  | H_8_ | 7.50 | 7.18  (-0.32) | - | - | 7.20  (-0.30) | - | - | 7.23  (-0.27) | - | - |
|  | H_9_ | 8.45 | 8.30  (-0.15) | - | - | 8.32  (-0.13) | - | - | 8.34  (-0.11) | - | - |
| Cym_΄_ | H_2΄c/6΄c_ | 6.52 | 6.42  (-0.10) | 6.38  (-0.14) | - | 6.44  (-0.08) | 6.42  (-0.10) | - | 6.48  (-0.04) | 6.45  (-0.07) | - |
|  | H_3΄c/5΄c_ | 6.16 | 6.19  (+0.03) | 6.16  (0.00) | - | 6.18  (+0.02) | 6.16  (0.00) | - | 6.17  (+0.01) | 6.16  (0.00) | - |
|  | H_7΄c_ | 1.77 | 1.80  (+0.03) | - | - | 1.79  (+0.02) | - | - | 1.78  (+0.01) | - | - |
|  | H_8΄c_ | 2.36 | 2.31  (-0.05) | - | - | 2.31  (-0.05) | - | - | 2.31  (-0.05) | - | - |
|  | H_9΄c/10΄c_ | 0.72 | 0.67  (-0.05) | - | - | 0.68  (-0.04) | - | - | 0.69  (-0.03) | - | - |
| Cym | H_2c_ | 5.96 | 5.89  (-0.07) | - | - | 5.80  (-0.16) | - | - | 5.74  (-0.22) | - | - |
|  | H_3c_ | 5.33 | 5.30  (-0.03) | - | - | 5.27  (-0.06) | - | - | 5.24  (-0.09) | - | - |
|  | H_5c_ | 6.03 | 6.10  (+0.07) | - | - | 6.05  (+0.02) | - | - | 5.97  (-0.06) | - | - |
|  | H_6c_ | 5.81 | 5.61  (-0.20) | - | - | 5.59  (-0.22) | - | - | 5.59  (-0.22) | - | - |
|  | H_7c_ | 1.52 | 1.54  (+0.02) | - | - | 1.56  (+0.04) | - | - | 1.57  (+0.05) | - | - |
|  | H_8c_ | 1.94 | 1.97  (+0.03) | - | - | 2.00  (+0.06) | - | - | 2.04  (+0.10) | - | - |
|  | H_9c_ | 0.47 | 0.46  (-0.01) | - | - | 0.38  (-0.09) | - | - | 0.30  (-0.17) | - | - |
|  | H_10c_ | 0.38 | 0.34  (-0.04) | - | - | 0.32  (-0.06) | - | - | 0.25  (-0.13) | - | - |
| BL-1 | H_a’_ | 8.43 | 8.35  (-0.08) | 8.39  (-0.04) | 8.35  (-0.08) | 8.36  (-0.07) | 8.39  (-0.04) | 8.35  (-0.08) | 8.39  (-0.04) | 8.43  (0.00) | 8.39  (-0.04) |
|  | H_b’_ | 7.33 | 7.38  (+0.05) | - | 7.39  (+0.04) | 7.37  (+0.04) | 7.43  (+0.10) | 7.37  (+0.04) | 7.37  (+0.04) | 7.44  (+0.11) | 7.36  (+0.05) |
|  | H_a_ | 8.46 | 8.31  (-0.15) | 8.31  (-0.15) | - | 8.33  (-0.13) | 8.33  (-0.13) | - | 8.35  (-0.11) | 8.33  (-0.13) | - |
|  | H_b_ | 7.13 | 7.16  (+0.03) | - | - | 7.16  (+0.03) | 7.15  (+0.02) | - | 7.16  (+0.03) | 7.13  (+0.00) | - |

**Table S5**: Differences in ^1^H chemical shifts of the d(5′-CGCGAATTGGCC-3′)_2_ (buffer phosphate 100 mM, pH = 7.0) upon the addition of complex (**4a**) in various [Ru]/nucleotide ratios at 298 K, 500 MHz. Values in parenthesis denote upfield (-) or downfield (+) shifts from the free oligonucleotide under the same conditions.

|  | **r = 0** | | | | | | | | **r = 0.5** | | | | | | | | **r = 1** | | | | | | | | **r = 2** | | | | | | | |
| --- | --- | --- | --- | --- | --- | --- | --- | --- | --- | --- | --- | --- | --- | --- | --- | --- | --- | --- | --- | --- | --- | --- | --- | --- | --- | --- | --- | --- | --- | --- | --- | --- |
|  | **H8/6** | **H5/H2**  **-CH_3_** | **H1’** | **H2’** | **H2’’** | **H3’** | **H4’** | **H5’5’’** | **H8/6** | **H5/H2**  **-CH_3_** | **H1’** | **H2’** | **H2’’** | **H3’** | **H4’** | **H5’5’’** | **H8/6** | **H5/H2**  **-CH_3_** | **H1’** | **H2’** | **H2’’** | **H3’** | **H4’** | **H5’5’’** | **H8/6** | **H5/H2**  **-CH_3_** | **H1’** | **H2’** | **H2’’** | **H3’** | **H4’** | **H5’5’’** |
| **C1** | 7.62 | 5.88 | 5.72 | 1.89 | 2.37 | 4.60 | 3.91 | 3.69  3.72 | 7.62  (0.00) | 6.00  (+0.12) | 5.88  (+0.16) | 1.87  (-0.02) | 2.39  (+0.02) | 4.73  (+0.13) | 4.06  (+0.15) | 3.76  (+0.07)  3.79  (+0.07) | 7.61  (-0.01) | 6.02  (+0.14) | 5.93  (+0.21) | 1.80  (-0.09) | 2.36  (-0.01) | 4.74  (+0.14) | 4.09  (+0.18) | 3.78  (+0.09)  3.81  (+0.09) | 7.57  (-0.05) | 6.02  (+0.14) | 5.89  (+0.17) | 1.71  (-0.18) | 2.28  (-0.09) | 4.74  (+0.14) | 4.09  (+0.18) | 3.79  (+0.10)  3.82  (+0.10) |
| **G2** | 7.93 | - | 5.88 | 2.53 | 2.65 | 4.97 | 4.36 | 3.97  4.08 | 8.03  (+0.10) | - | 5.75  (-0.13) | 2.40  (-0.13) | 2.69  (+0.04) | 4.89  (-0.08) | 4.42  (+0.06) | 4.07  (+0.10)  4.18  (+0.10) | 8.07  (+0.14) | - | 5.71  (-0.17) | 2.36  (-0.17) | 2.69  (+0.04) | 5.03  (+0.06) | 4.43  (+0.07) | 4.08  (+0.11)  4.18  (+0.10) | 8.11  (+0.18) | - | 5.65  (-0.23) | 2.29  (-0.24) | 2.70  (+0.05) | 5.04  (+0.07) | 4.43  (+0.07) | 4.09  (+0.12)  4.18  (+0.10) |
| **C3** | 7.25 | 5.35 | 5.56 | 1.81 | 2.22 | 4.77 | 4.10 | 4.12  4.15 | 7.33  (+0.08) | 5.50  (+0.15) | 5.62  (+0.06) | 1.98  (+0.17) | 2.24  (+0.02) | 4.85  (+0.08) | 4.10  (0.00) | 4.12  (0.00)  4.15  (0.00) | 7.36  (+0.11) | 5.57  (+0.22) | 5.65  (+0.09) | 2.06  (+0.25) | 2.25  (+0.03) | 4.89  (+0.12) | 4.12  (+0.02) | 4.14  (+0.02)  4.17  (+0.02) | 7.38  (+0.13) | 5.54  (+0.19) | 5.65  (+0.09) | 2.07  (+0.26) | 2.25  (+0.03) | 4.92  (+0.15) | 4.13  (+0.03) | 4.15  (+0.03)  4.18  (+0.03) |
| **G4** | 7.83 | - | 5.42 | 2.63 | 2.74 | 4.97 | 4.39 | 3.97  4.06 | 7.92  (+0.09) | - | 5.25  (-0.17) | 2.62  (-0.01) | 2.68  (-0.06) | 4.93  (-0.04) | 4.36  (-0.03) | 4.03  (+0.06)  4.12  (+0.06) | 7.96  (+0.13) | - | 5.15  (-0.27) | 2.61  (-0.02) | 2.65  (-0.09) | 4.91  (-0.06) | 4.36  (-0.03) | 4.07  (+0.10)  4.18  (+0.12) | 7.99  (+0.16) | - | 5.09  (-0.33) | 2.58  (-0.05) | 2.64  (-0.10) | 4.90  (-0.07) | 4.36  (-0.03) | 4.08  (+0.11)  4.18  (+0.12) |
| **A5** | 8.09 | 7.23 | 5.97 | 2.67 | 2.89 | 5.03 | 4.45 | 4.15  4.19 | 8.16  (+0.07) | **n.o.** | 5.78  (-0.19) | 2.68  (+0.01) | 2.80  (-0.07) | 5.03  (0.00) | 4.35  (-0.10) | 4.11  (-0.04)  4.19  (0.00) | 8.18  (+0.09) | **n.o.** | 5.66  (-0.31) | 2.68  (+0.01) | 2.80  (-0.07) | 5.04  (+0.01) | 4.27  (-0.18) | 4.11  (-0.04)  4.18  (-0.01) | 8.20  (+0.11) | **n.o.** | 5.56  (-0.41) | 2.68  (+0.01) | 2.75  (-0.12) | 5.04  (+0.01) | 4.27  (-0.18) | 4.09  (-0.06)  4.18  (-0.01) |
| **A6** | 8.09 | 7.60 | 6.13 | 2.57 | 2.90 | 4.99 | 4.45 | n.o.  4.25 | 8.19  (+0.10) | **n.o.** | 6.11  (-0.02) | 2.62  (+0.05) | 2.85  (-0.05) | 5.00  (+0.01) | 4.46  (+0.01) | n.o.  4.21  (-0.04) | 8.23  (+0.14) | **n.o.** | 6.11  (-0.02) | 2.66  (+0.09) | 2.85  (-0.05) | 5.00  (+0.01) | 4.46  (+0.01) | n.o.  4.19  (-0.06) | 8.28  (+0.19) | **n.o.** | 6.05  (-0.08) | 2.67  (+0.10) | 2.82  (-0.08) | 5.00  (+0.01) | 4.46  (+0.01) | n.o.  4.17  (-0.08) |
| **T7** | 7.09 | 1.25 | 5.88 | 1.95 | 2.53 | 4.81 | 4.14 | 4.15 | 7.16  (+0.07) | 1.34  (+0.09) | 5.83  (-0.05) | 2.02  (+0.07) | 2.48  (-0.05) | 4.83  (+0.02) | 4.27  (+0.13) | 4.15  (0.00) | 7.18  (+0.09) | 1.38  (+0.13) | 5.80  (-0.08) | 2.03  (+0.08) | 2.44  (-0.09) | 4.84  (+0.03) | 4.27  (+0.13) | 4.12  (-0.03) | 7.19  (+0.10) | 1.45  (+0.20) | 5.74  (-0.14) | 2.03  (+0.08) | 2.37  (-0.16) | 4.84  (+0.03) | 4.40  (+0.26) | 4.06  (-0.09) |
| **T8** | 7.35 | 1.51 | 6.08 | 2.14 | 2.53 | 4.88 | 4.19 | 4.09 | 7.39  (+0.04) | 1.64  (+0.13) | 6.03  (-0.05) | 2.06  (-0.08) | 2.47  (-0.06) | 4.87  (-0.01) | 4.24  (+0.05) | 4.07  (-0.02) | 7.39  (+0.04) | 1.70  (+0.19) | 6.00  (-0.08) | 2.05  (-0.09) | 2.43  (-0.10) | 4.87  (-0.01) | 4.24  (+0.05) | 4.07  (-0.02) | 7.43  (+0.08) | 1.75  (+0.24) | 6.02  (-0.06) | 2.05  (-0.09) | 2.40  (-0.13) | 4.87  (-0.01) | 4.24  (+0.05) | 4.06  (-0.03) |
| **C9** | 7.44 | 5.60 | 5.64 | 2.04 | 2.39 | 4.86 | 4.14 | 4.10  4.17 | 7.45  (+0.01) | 5.84  (+0.24) | 5.73  (+0.09) | 2.03  (-0.01) | 2.43  (+0.04) | 4.91  (+0.05) | 4.12  (-0.02) | 4.09  (-0.01)  4.16  (-0.01) | 7.44  (0.00) | 5.94  (+0.34) | 5.76  (+0.12) | 2.02  (-0.02) | 2.43  (+0.04) | 4.95  (+0.09) | 4.12  (-0.02) | 4.06  (-0.04)  4.12  (-0.05) | 7.44  (0.00) | 6.03  (+0.43) | 5.73  (+0.11) | 2.00  (-0.04) | 2.43  (+0.04) | 4.95  (+0.09) | 4.12  (-0.02) | 4.03  (-0.07)  4.10  (-0.07) |
| **G10** | 7.89 | - | 5.83 | 2.54 | 2.67 | 4.97 | 4.41 | 4.04  4.15 | 8.03  (+0.14) | - | 5.77  (-0.06) | 2.41  (-0.13) | 2.69  (+0.02) | 5.00  (+0.03) | 4.42  (+0.01) | 4.07  (+0.03)  4.18  (+0.03) | 8.08  (+0.19) | - | 5.66  (-0.17) | 2.41  (-0.13) | 2.70  (+0.03) | 5.02  (+0.05) | 4.47  (+0.06) | 4.09  (+0.05)  4.20  (+0.05) | 8.14  (+0.25) | - | 5.61  (-0.22) | 2.38  (-0.16) | 2.70  (+0.03) | 5.01  (+0.04) | 4.44  (+0.03) | 4.08  (+0.04)  4.18  (+0.03) |
| **C11** | 7.31 | 5.42 | 5.72 | 1.85 | 2.31 | 4.78 | 4.18 | 4.09  n.o. | 7.35  (+0.04) | 5.48  (+0.06) | 5.62  (-0.10) | 2.03  (+0.18) | 2.29  (-0.02) | 4.87  (+0.09) | 4.15  (-0.03) | 4.07  (-0.02)  n.o. | 7.38  (+0.07) | 5.51  (+0.09) | 5.51  (-0.21) | 2.06  (+0.21) | 2.26  (-0.05) | 4.88  (+0.10) | 4.15  (-0.03) | 4.07  (-0.02)  n.o. | 7.39  (+0.08) | 5.63  (+0.21) | 5.48  (-0.24) | 2.07  (+0.22) | 2.22  (-0.09) | 4.92  (+0.14) | 4.15  (-0.03) | 4.06  (-0.03)  n.o. |
| **G12** | 7.92 | - | 6.13 | 2.37 | 2.58 | 4.66 | 4.19 | n.o.  n.o. | 7.98  (+0.06) | - | 6.10  (-0.03) | 2.41  (+0.04) | 2.69  (+0.11) | 4.68  (+0.02) | 4.14  (-0.05) | n.o.  n.o. | 7.99  (+0.07) | - | 6.06  (-0.07) | 2.42  (+0.05) | 2.73  (+0.15) | 4.68  (+0.02) | 4.14  (-0.05) | n.o.  n.o. | 7.99  (+0.07) | - | 5.99  (-0.14) | 2.42  (+0.05) | 2.74  (+0.16) | 4.68  (+0.02) | 4.14  (-0.05) | n.o.  n.o. |

**Table S6** ^1^H NMR chemical shifts of the (**5a**) (H_2_O : D_2_O, 9 : 1, 298 K, buffer phosphates 100 mM, pH = 7.0) free, and upon the addition to the d(5′-CGCGAATTGGCC-3′)_2_ at r = 0.5, 1, and 2. Shifts are denoting in parenthesis (negative sign upfield and positive sign downfield shifts). A and B denotes the signals of the two moieties of (**5a**). n.o. = not observed.

|  |  |  | **r = 0.5** | | **r = 1** | | **r = 2** | |
| --- | --- | --- | --- | --- | --- | --- | --- | --- |
| Ligands | Protons | (**5**)Cl_3_ | A | B | A | B | A | B |
| phe | H_2΄/9΄_ | 9.97 | 9.92  (-0.05) | 9.89  (-0.08) | 9.93  (-0.06) | 9.90  (-0.07) | 9.97  (0.00) | 9.92  (-0.05) |
|  | H_3΄/8΄_ | 8.24 | 8.16  (-0.08) | 8.07  (-0.17) | 8.17  (-0.07) | 8.10  (-0.14) | 8.18  (-0.06) | 8.12  (-0.05) |
|  | H_4΄/7΄_ | 8.85 | 8.67  (-0.18) | 8.62  (-0.23) | 8.68  (-0.17) | 8.64  (-0.21) | 8.70  (-0.15) | 8.66  (-0.19) |
|  | H_5΄/6΄_ | 8.10 | 7.81  (-0.29) | 7.87  (-0.23) | 7.83  (-0.27) | 7.88  (-0.22) | 7.85  (-0.25) | 7.89  (-0.21) |
| bq | H_2_ | 9.75 | 9.63  (-0.12) | 9.59  (-0.16) | 9.62  (-0.13) | 9.58  (-0.17) | 9.61  (-0.14) | 9.58  (-0.17) |
|  | H_3_ | 7.71 | 7.55  (-0.16) | 7.41  (-0.30) | 7.55  (-0.16) | 7.42  (-0.29) | 7.55  (-0.16) | 7.43  (-0.28) |
|  | H_4_ | 8.29 | 8.06  (-0.23) | 7.99  (-0.30) | 8.02  (-0.27) | 7.98  (-0.31) | 8.00  (-0.29) | 7.95  (-0.34) |
|  | H_5_ | 7.49 | 7.23  (-0.26) | - | 7.20  (-0.29) | - | 7.20  (-0.29) | - |
|  | H_6_ | 7.44 | 7.23  (-0.21) | - | 7.21  (-0.23) | - | 7.18  (-0.26) | - |
|  | H_7_ | 7.36 | 7.17  (-0.19) | - | 7.14  (-0.22) | - | 7.10  (-0.26) |  |
|  | H_8_ | 7.58 | 7.34  (-0.24) | - | 7.30  (-0.28) | - | 7.31  (-0.27) | - |
|  | H_9_ | 8.56 | 8.39  (-0.17) | - | 8.38  (-0.18) | - | 8.36  (-0.20) | - |
| Cym΄ | H_2΄c/6΄c_ | 6.49 | 6.42  (-0.07) | - | 6.42  (-0.07) | - | 6.44  (-0.05) | - |
|  | H_3΄c/5΄c_ | 5.70/5.76 | 5.82  (+0.12/+0.06) | - | 5.84  (+0.14/+0.08) | - | 5.85  (+0.15/+0.07) | - |
|  | H_7΄c_ | 0.82 | 1.04  (+0.22) | - | 1.05  (+0.23) | - | 1.08  (+0.26) | - |
|  | H_8΄c_ | 2.33 | 2.28  (-0.05) | - | 2.28  (-0.05) | - | 2.28  (-0.05) | - |
|  | H_9΄c/10΄c_ | 0.68/0.69 | 0.64  (-0.04/-0.05) | - | 0.64  (-0.04/-0.05) | - | 0.64  (-0.04) | 0.65  (-0.04) |
| Cym | H_2c_ | 6.07 | 5.91  (-0.16) | - | 5.89  (-0.18) | - | 5.85  (-0.22) | - |
|  | H_3c_ | 5.27 | 5.22  (-0.04) | - | 5.22  (-0.05) | - | 5.20  (-0.07) | - |
|  | H_5c_ | 6.00 | 5.90  (-0.10) | - | 5.89  (-0.11) | - | 5.87  (-0.13) | - |
|  | H_6c_ | 5.83 | 5.70  (-0.13) | - | 5.69  (-0.14) | - | 5.67  (-0.16) | - |
|  | H_7c_ | 1.26 | 1.24  (-0.02) | - | 1.22 (-0.04) | - | 1.19  (-0.07) | - |
|  | H_8c_ | 2.00 | 1.93  (-0.07) | - | 1.89  (-0.11) | - | 1.85  (-0.15) | - |
|  | H_9c_ | 0.55 | 0.43  (-0.12) | - | 0.39  (-0.16) | - | 0.38  (-0.17) | - |
|  | H_10c_ | 0.51 | 0.40  (-0.11) | - | 0.39  (-0.12) | - | 0.37  (-0.14) | - |
| BL-2 | H_a’_ | 7.99 | 8.00  (+0.01) | - | 8.01  (+0.02) | - | 8.02  (+0.03) | - |
|  | H_b’_ | 6.80 | 6.87  (+0.07) | - | 6.85  (+0.05) | - | 6.83  (+0.03) | - |
|  | H_a_ | 8.05 | 8.04  (-0.01) | 8.02  (-0.03) | 8.03  (-0.02) | 8.00  (-0.05) | 7.99  (-0.06) | 7.98  (-0.07) |
|  | H_b_ | 6.42 | 6.61  (+0.19) | 6.56  (+0.14) | 6.55  (+0.13) | 6.50  (+0.08) | 6.49  (+0.07) | 6.44  (+0.02) |
|  | H_c_/H_c’_ | 2.49 | 2.50  (+0.01) | - | 2.49  (0.00) | - | 2.49  (0.00) | - |

**Table S7**: Differences in ^1^H chemical shifts of the d(5′-CGCGAATTGGCC-3′)_2_ (buffer phosphate 100 mM, pH = 7.0) upon the addition of complex (**5a**) in various [Ru]/nucleotide ratios at 298 K, 500 MHz. Values in parenthesis denote upfield (-) or downfield (+) shifts from the free oligonucleotide under the same conditions.

|  | **r = 0** | | | | | | | | **r = 0.5** | | | | | | | | **r = 1** | | | | | | | | **r = 2** | | | | | | | |
| --- | --- | --- | --- | --- | --- | --- | --- | --- | --- | --- | --- | --- | --- | --- | --- | --- | --- | --- | --- | --- | --- | --- | --- | --- | --- | --- | --- | --- | --- | --- | --- | --- |
|  | **H8/6** | **H5/H2**  **-CH_3_** | **H1’** | **H2’** | **H2’’** | **H3’** | **H4’** | **H5’5’’** | **H8/6** | **H5/H2**  **-CH_3_** | **H1’** | **H2’** | **H2’’** | **H3’** | **H4’** | **H5’5’’** | **H8/6** | **H5/H2**  **-CH_3_** | **H1’** | **H2’** | **H2’’** | **H3’** | **H4’** | **H5’5’’** | **H8/6** | **H5/H2**  **-CH_3_** | **H1’** | **H2’** | **H2’’** | **H3’** | **H4’** | **H5’5’’** |
| **C1** | 7.62 | 5.88 | 5.72 | 1.89 | 2.37 | 4.60 | 3.91 | 3.69  3.72 | 7.63  (+0.01) | 5.92  (+0.04) | 5.77  (+0.05) | 1.93  (+0.04) | 2.39  (+0.02) | 4.70  (+0.10) | 4.07  (+0.16) | 3.71  (+0.02)  3.73  (+0.01) | 7.62  (0.00) | 5.91  (+0.03) | 5.77  (+0.05) | 1.94  (+0.05) | 2.39  (+0.02) | 4.71  (+0.11) | 4.07  (+0.16) | 3.71  (+0.02)  3.73  (+0.01) | 7.61  (-0.01) | 5.91  (+0.03) | 5.77  (+0.05) | 1.94  (+0.05) | 2.39  (+0.02) | 4.71  (+0.11) | 4.07  (+0.16) | 3.71  (+0.02)  3.73  (+0.01) |
| **G2** | 7.93 | - | 5.88 | 2.53 | 2.65 | 4.97 | 4.36 | 3.97  4.08 | 7.95  (+0.02) | - | 5.83  (-0.05) | 2.65  (+0.12) | 2.70  (+0.05) | 5.05  (+0.08) | 4.42  (+0.06) | 4.16  (+0.19)  4.25  (+0.17) | 7.96  (+0.03) | - | 5.82  (-0.06) | 2.65  (+0.12) | 2.70  (+0.05) | 5.05  (+0.08) | 4.40  (+0.04) | 4.16  (+0.19)  4.25  (+0.17) | 7.96  (+0.03) | - | 5.81  (-0.07) | 2.65  (+0.12) | 2.70  (+0.05) | 5.05  (+0.08) | 4.40  (+0.04) | 4.16  (+0.19)  4.25  (+0.17) |
| **C3** | 7.25 | 5.35 | 5.56 | 1.81 | 2.22 | 4.77 | 4.10 | 4.12  4.15 | 7.28  (+0.03) | 5.41  (+0.06) | 5.59  (+0.03) | 1.89  (+0.07) | 2.26  (+0.04) | 4.70  (-0.07) | 4.07  (-0.03) | 4.00  (-0.12)  3.96  (-0.19) | 7.28  (+0.03) | 5.43  (+0.08) | 5.59  (+0.03) | 1.90  (+0.08) | 2.26  (+0.04) | 4.70  (-0.07) | 4.07  (-0.03) | 4.04  (-0.08)  4.00  (-0.15) | 7.29  (+0.04) | 5.45  (+0.10) | 5.59  (+0.03) | 1.91  (+0.09) | 2.26  (+0.04) | 4.72  (-0.05) | 4.07  (-0.03) | 4.04  (-0.08)  4.00  (-0.15) |
| **G4** | 7.83 | - | 5.42 | 2.63 | 2.74 | 4.97 | 4.39 | 3.97  4.06 | 7.85  (+0.02) | - | 5.40  (-0.02) | 2.62  (-0.01) | 2.67  (-0.07) | 4.97  (0.00) | 4.40  (+0.01) | 4.04  (+0.07)  4.14  (+0.08) | 7.86  (+0.03) | - | 5.38  (-0.04) | 2.66  (+0.03) | 2.70  (-0.04) | 4.98  (+0.01) | 4.40  (+0.01) | 4.19  (+0.22)  4.24  (+0.18) | 7.87  (+0.04) | - | 5.35  (-0.07) | 2.61  (-0.02) | 2.69  (-0.05) | 4.98  (+0.01) | 4.40  (+0.01) | 4.19  (+0.22)  4.24  (+0.18) |
| **A5** | 8.09 | 7.23 | 5.97 | 2.67 | 2.89 | 5.03 | 4.45 | 4.15  4.19 | 8.10  (+0.01) | 7.21  (-0.02) | 5.96  (-0.01) | 2.70  (+0.03) | 2.88  (-0.01) | 5.05  (+0.02) | 4.43  (-0.02) | 4.19  (+0.04)  4.23  (+0.04) | 8.11  (+0.02) | 7.20  (-0.03) | 5.93  (-0.04) | 2.71  (+0.04) | 2.88  (-0.01) | 5.05  (+0.02) | 4.42  (-0.03) | 4.19  (+0.04)  4.24  (+0.05) | 8.12  (+0.03) | 7.19  (-0.04) | 5.91  (-0.06) | 2.70  (+0.03) | 2.87  (-0.02) | 5.06  (+0.03) | 4.41  (-0.04) | 4.19  (+0.04)  4.24  (+0.05) |
| **A6** | 8.09 | 7.60 | 6.13 | 2.57 | 2.90 | 4.99 | 4.45 | n.o.  4.25 | 8.10  (+0.01) | 7.59  (-0.01) | 6.12  (-0.01) | 2.55  (-0.02) | 2.90  (0.00) | 4.99  (0.00) | 4.43  (-0.02) | n.o.  4.24  (-0.01) | 8.11  (+0.02) | 7.57  (-0.03) | 6.12  (-0.01) | 2.54  (-0.03) | 2.89  (-0.01) | 5.00  (+0.01) | 4.42  (-0.03) | n.o.  4.25  (0.00) | 8.12  (+0.03) | 7.57  (-0.03) | 6.12  (-0.01) | 2.53  (-0.04) | 2.86  (-0.04) | 4.99  (+0.00) | 4.39  (-0.06) | n.o.  4.22  (-0.03) |
| **T7** | 7.09 | 1.25 | 5.88 | 1.95 | 2.53 | 4.81 | 4.14 | 4.15 | 7.10  (+0.01) | 1.28  (+0.03) | 5.88  (0.00) | 1.96  (+0.01) | 2.54  (+0.01) | n.o. | 4.15  (+0.01) | n.o.  4.15  (0.00) | 7.10  (+0.01) | 1.29  (+0.04) | 5.88  (0.00) | 1.99  (+0.04) | 2.54  (+0.01) | n.o. | 4.15  (+0.01) | n.o.  4.15  (0.00) | 7.11  (+0.02) | 1.30  (+0.05) | 5.86  (-0.02) | 1.99  (+0.04) | 2.51  (-0.02) | 4.87  (+0.06) | 4.15  (+0.01) | n.o.  4.15  (0.00) |
| **T8** | 7.35 | 1.51 | 6.08 | 2.14 | 2.53 | 4.88 | 4.19 | 4.09 | 7.36  (+0.01) | 1.55  (+0.04) | 6.07  (-0.01) | 2.09  (-0.05) | 2.51  (-0.02) | 4.88  (0.00) | 4.16  (-0.03) | n.o.  4.13  (+0.04) | 7.36  (+0.01) | 1.55  (+0.04) | 6.05  (-0.03) | 2.10  (-0.04) | 2.50  (-0.03) | 4.88  (0.00) | 4.14  (-0.05) | n.o.  4.13  (+0.04) | 7.36  (+0.01) | 1.56  (+0.05) | 6.05  (-0.03) | 2.09  (-0.05) | 2.49  (-0.04) | 4.88  (0.00) | 4.14  (-0.05) | n.o.  4.13  (+0.04) |
| **C9** | 7.44 | 5.60 | 5.64 | 2.04 | 2.39 | 4.86 | 4.14 | 4.10  4.17 | 7.44  (0.00) | 5.65  (+0.05) | 5.67  (+0.03) | 2.04  (0.00) | 2.36  (-0.03) | 4.86  (0.00) | 4.12  (-0.02) | 4.02  (-0.08)  4.08  (-0.09) | 7.44  (0.00) | 5.69  (+0.09) | 5.69  (+0.05) | 2.05  (+0.01) | 2.36  (-0.03) | 4.86  (0.00) | 4.09  (-0.05) | 3.98  (-0.12)  4.06  (-0.11) | 7.44  (0.00) | 5.71  (+0.11) | 5.69  (+0.05) | 2.04  (0.00) | 2.36  (-0.03) | 4.86  (0.00) | 4.08  (-0.06) | 3.98  (-0.12)  4.06  (-0.11) |
| **G10** | 7.89 | - | 5.83 | 2.54 | 2.67 | 4.97 | 4.41 | 4.04  4.15 | 7.92  (+0.03) | - | 5.83  (0.00) | 2.64  (+0.10) | 2.70  (+0.03) | 5.04  (+0.07) | 4.43  (+0.02) | 4.18  (+0.14)  4.26  (+0.11) | 7.92  (+0.03) | - | 5.82  (-0.01) | 2.65  (+0.11) | 2.70  (+0.03) | 5.05  (+0.08) | 4.43  (+0.02) | 4.18  (+0.14)  4.27  (+0.12) | 7.93  (+0.04) | - | 5.81  (-0.02) | 2.61  (+0.07) | 2.69  (+0.03) | 4.99  (+0.02) | 4.39  (-0.02) | 4.18  (+0.14)  4.27  (+0.12) |
| **C11** | 7.31 | 5.42 | 5.72 | 1.85 | 2.31 | 4.78 | 4.18 | 4.09  n.o. | 7.33  (+0.02) | 5.45  (+0.03) | 5.69  (-0.03) | 1.86  (+0.01) | 2.29  (-0.02) | 4.70  (-0.08) | 4.09  (-0.09) | 4.02  (-0.07)  n.ο. | 7.33  (+0.02) | 5.46  (+0.04) | 5.67  (-0.05) | 1.88  (+0.03) | 2.28  (-0.03) | 4.70  (-0.08) | 4.06  (-0.12) | 4.02  (-0.07)  n.ο. | 7.33  (+0.02) | 5.47  (+0.05) | 5.65  (-0.07) | 1.86  (+0.01) | 2.28  (-0.03) | 4.71  (-0.07) | 4.07  (-0.11) | 4.02  (-0.07)  n.ο. |
| **G12** | 7.92 | - | 6.13 | 2.37 | 2.58 | 4.66 | 4.19 | n.o.  n.o. | 7.92  (0.00) | - | 6.08  (-0.05) | 2.32  (-0.05) | 2.57  (-0.01) | 4.67  (+0.01) | 4.16  (-0.03) | n.o.  n.o. | 7.92  (0.00) | - | 6.02  (-0.11) | 2.32  (-0.05) | 2.57  (-0.01) | 4.67  (+0.01) | 4.16  (-0.03) | n.o.  n.o. | 7.92  (0.00) | - | 5.97  (-0.16) | 2.28  (-0.09) | 2.57  (-0.01) | 4.66  (0.00) | 4.14  (-0.05) | n.o.  n.o. |

**Table S8** ^1^H NMR chemical shifts of the (**6a**) (H_2_O : D_2_O, 9 : 1, 298 K, buffer phosphates 100 mM, pH = 7.0) free, and upon the addition to the d(5′-CGCGAATTGGCC-3′)_2_ at r = 0.5, 1, and 2. Shifts are denoting in parenthesis (negative sign upfield and positive sign downfield shifts). A and B denotes the signals of the two moieties of (**6a**). n.o. = not observed.

|  |  |  | **r = 0.5** | | | **r = 1** | | | **r = 2** | |
| --- | --- | --- | --- | --- | --- | --- | --- | --- | --- | --- |
| Ligands | Protons/r | (**6a**) | A | B | A | | B | A | | B |
| phen | H_2΄/9΄_ | 10.12 | 9.92b  (-0.20) | 9.94b  (-0.18) | 9.94b  (-0.18) | | 9.96b  (-0.16) | 9.98b  (-0.14) | | 10.00b  (-0.12) |
|  | H_3΄/8΄_ | 8.29 | 8.00  (-0.29) | - | 8.04  (-0.25) | | - | 8.09  (-0.20) | | - |
|  | H_4΄/7΄_ | 8.68 | 8.36  (-0.32) | 8.33  (-0.35) | 8.42  (-0.26) | | 8.38  (-0.30) | 8.48  (-0.20) | | 8.44  (-0.24) |
|  | H_5΄/6΄_ | 7.58 | 7.53  (-0.05) | - | 7.54  (-0.04) | | - | 7.56  (-0.02) | | - |
| bq | H_2_ | 9.60 | 9.46b  (-0.14) | 9.45b  (-0.15) | 9.47b  (-0.13) | | 9.46b  (-0.14) | 9.50b  (-0.10) | | 9.49b  (-0.11) |
|  | H_3_ | 7.57 | 7.30  (-0.27) | 7.17  (-0.40) | 7.36  (-0.21) | | 7.25  (-0.32) | 7.40  (-0.17) | | 7.32  (-0.25) |
|  | H_4_ | 7.65 | 7.61  (-0.04) | 7.50  (-0.15) | 7.63  (-0.02) | | 7.52  (-0.13) | 7.65  (0.00) | | 7.58  (-0.07) |
|  | H_5_ | 6.33 | n.o. | - | n.o. | | - | 6.31  (-0.02) | | - |
|  | H_6_ | 6.47 | n.o. | - | n.o. | | - | 6.33  (-0.14) | | 6.31  (-0.16) |
|  | H_7_ | 6.63 | n.o. | - | n.o. | | - | 6.59  (-0.04) | | - |
|  | H_8_ | 7.30 | 6.98  (-0.32) | - | 7.03  (-0.27) | | - | 7.08  (-0.22) | | - |
|  | H_9_ | 8.34 | 8.16  (-0.18) | - | 8.17  (-0.17) | | - | 8.20  (-0.14) | | - |
| Cym΄ | H_2΄c/6΄c_ | 6.53 | 6.36  (-0.17) | 6.38  (-0.15) | 6.39  (-0.14) | | 6.40  (-0.13) | 6.42  (-0.11) | | 6.43  (-0.10) |
|  | H_3΄c/5΄c_ | 6.17 | 6.04  (-0.13) | 6.06  (-0.11) | 6.06  (-0.11) | | 6.08  (-0.09) | 6.09  (-0.08) | | 6.10  (-0.07) |
|  | H_7΄c_ | 1.77 | 1.66  (-0.11) | - | 1.68  (-0.09) | | - | 1.70  (-0.07) | | - |
|  | H_8΄c_ | 2.37 | 2.22  (-0.15) | - | 2.25  (-0.12) | | - | 2.27  (-0.10) | | - |
|  | H_9΄c/10΄c_ | 0.73 | 0.63  (-0.10) | - | 0.65  (-0.08) | | - | 0.66  (-0.07) | | - |
| Cym | H_2c_ | 6.02 | 5.81  (-0.21) | - | 5.85  (-0.17) | | - | 5.87  (-0.15) | | - |
|  | H_3c_ | 5.29 | 5.25  (-0.04) | 5.20  (-0.09) | 5.24  (-0.05) | | 5.20  (-0.09) | 5.23  (-0.06) | | 5.19  (-0.10) |
|  | H_5c_ | 6.02 | 6.06  (+0.04) | - | 6.07  (+0.05) | | - | 6.08  (+0.06) | | - |
|  | H_6c_ | 5.81 | 5.61  (-0.20) | - | 5.62  (-0.19) | | - | 5.64  (-0.17) | | - |
|  | H_7c_ | 1.39 | 1.35  (-0.04) | 1.38  (-0.01) | 1.32  (-0.06) | | 1.35  (-0.04) | 1.31  (-0.08) | | 1.33  (-0.06) |
|  | H_8c_ | 1.97 | 1.85  (-0.12) | - | 1.84  (-0.13) | | - | 1.83  (-0.14) | | - |
|  | H_9c_ | 0.50 | 0.45  (-0.05) | - | 0.42  (-0.08) | | - | 0.38  (-0.12) | | - |
|  | H_10c_ | 0.49 | 0.40  (-0.09) | - | 0.32  (-0.17) | | - | 0.31  (-0.18) | | - |
| BL-3 | H_a’_ | 8.36 | 8.25  (-0.13) | 8.22  (-0.16) | 8.25  (-0.13) | | 8.24  (-0.14) | 8.26  (-0.12) | | 8.26  (-0.12) |
|  | H_b’_ | 6.89 | 7.09  (+0.20) | 7.06  (+0.17) | 7.04  (+0.15) | | 7.02  (+0.13) | 6.99  (+0.10) | | 6.99  (+0.10) |
|  | H_a_ | 7.43 | 7.86  (+0.43) | - | 7.79 | | - | 7.72  (+0.29) | | - |
|  | H_b_ | 5.27 | 6.37  (+1.10) | - | 6.19 | | - | 6.03  (+0.69) | | - |
|  | H_c΄_/H_c_ | 1.96/1.19 | 2.27/2.22  (+0.31/+1.03) | - | 2.19/2.22  (+0.23/+1.03) | | - | 2.12/2.22  (+0.16/+1.03) | | - |
|  | H_d΄_/H_d_ | 0.78/0.76 | 1.28  (+0.50/+0.52) | - | 1.18/1.32  (+0.40/+0.56) | | - | 1.11/1.34  (+0.33/+0.58) | | - |

**Table S9**: Differences in ^1^H chemical shifts of the d(5′-CGCGAATTGGCC-3′)_2_ (buffer phosphate 100 mM, pH = 7.0) upon the addition of complex (**6a**) in various [Ru]/nucleotide ratios at 298 K, 500 MHz. Values in parenthesis denote upfield (-) or downfield (+) shifts from the free oligonucleotide under the same conditions.

|  | **r = 0** | | | | | | | | **r = 0.5** | | | | | | | | **r = 1** | | | | | | | | **r = 2** | | | | | | | | |
| --- | --- | --- | --- | --- | --- | --- | --- | --- | --- | --- | --- | --- | --- | --- | --- | --- | --- | --- | --- | --- | --- | --- | --- | --- | --- | --- | --- | --- | --- | --- | --- | --- | --- |
|  | **H8/6** | **H5/H2**  **-CH_3_** | **H1’** | **H2’** | **H2’’** | **H3’** | **H4’** | **H5’5’’** | **H8/6** | **H5/H2**  **-CH_3_** | **H1’** | **H2’** | **H2’’** | **H3’** | **H4’** | **H5’5’’** | **H8/6** | **H5/H2**  **-CH_3_** | **H1’** | **H2’** | **H2’’** | **H3’** | **H4’** | **H5’5’’** | | **H8/6** | **H5/H2**  **-CH_3_** | **H1’** | **H2’** | **H2’’** | **H3’** | **H4’** | **H5’5’’** |
| **C1** | 7.60 | 5.88 | 5.72 | 1.89 | 2.37 | 4.60 | 3.91 | 3.69  3.72 | 7.63  (+0.03) | 5.92  (+0.04) | 5.76  (+0.04) | 1.88  (-0.01) | 2.35  (-0.02) | 4.70  (+0.10) | 4.07  (+0.16) | 3.72  (+0.03)  3.74  (+0.02) | 7.63  (+0.03) | 5.94  (+0.06) | 5.77  (+0.05) | 1.83  (-0.06) | 2.32  (-0.05) | 4.71  (+0.11) | 4.08  (+0.17) | 3.73  (+0.04)  3.75  (+0.03) | | 7.63  (+0.03) | 5.94  (+0.06) | 5.77  (+0.05) | 1.78  (-0.11) | 2.29  (-0.08) | 4.71  (+0.11) | 4.08  (+0.17) | 3.73  (+0.04)  3.75  (+0.03) |
| **G2** | 7.93 | - | 5.88 | 2.53 | 2.65 | 4.97 | 4.36 | 3.97  4.08 | 7.96  (+0.03) | - | 5.81  (-0.07) | 2.64  (+0.11) | 2.69  (+0.04) | 4.97  (0.00) | 4.31  (-0.05) | 3.98  (+0.01)  4.08  (0.00) | 7.97  (+0.04) | - | 5.77  (-0.11) | 2.65  (+0.12) | 2.70  (+0.05) | 4.98  (+0.01) | 4.31  (-0.05) | 4.03  (+0.06)  4.13  (+0.05) | | 7.99  (+0.06) | - | 5.60  (-0.28) | 2.65  (+0.12) | 2.70  (+0.05) | 5.01  (+0.04) | 4.30  (-0.06) | 4.04  (+0.07)  4.14  (+0.06) |
| **C3** | 7.25 | 5.35 | 5.56 | 1.81 | 2.22 | 4.77 | 4.10 | 4.12  4.15 | 7.30  (+0.05) | 5.46  (+0.11) | 5.56  (0.00) | 1.86  (+0.05) | 2.24  (+0.02) | 4.72  (-0.05) | 4.09  (-0.01) | 4.12  (0.00)  4.15  (0.00) | 7.34  (+0.09) | 5.53  (+0.18) | 5.56  (0.00) | 1.87  (+0.06) | 2.24  (+0.02) | 4.72  (-0.05) | 4.07  (-0.03) | 4.09  (-0.03)  4.12  (-0.03) | | 7.37  (+0.12) | 5.61  (+0.26) | 5.56  (0.00) | 1.89  (+0.08) | 2.24  (+0.02) | 4.72  (-0.05) | 4.07  (-0.03) | 4.07  (-0.05)  4.11  (-0.04) |
| **G4** | 7.83 | - | 5.42 | 2.63 | 2.74 | 4.97 | 4.39 | 3.97  4.06 | 7.84  (+0.01) | - | 5.37  (-0.05) | 2.61  (-0.02) | 2.71  (-0.03) | 4.95  (-0.02) | 4.27  (-0.12) | 3.99  (+0.02)  4.08  (+0.02) | 7.85  (+0.02) | - | 5.33  (-0.09) | 2.60  (-0.03) | 2.71  (-0.03) | 4.95  (-0.02) | 4.25  (-0.14) | 4.03  (+0.06)  4.13  (+0.07) | | 7.86  (+0.03) | - | 5.27  (-0.15) | 2.58  (-0.05) | 2.70  (-0.04) | 4.94  (-0.03) | 4.23  (-0.16) | 4.03  (+0.06)  4.13  (+0.07) |
| **A5** | 8.09 | 7.23 | 5.97 | 2.67 | 2.89 | 5.03 | 4.45 | 4.15  4.19 | 8.10  (+0.01) | 7.21  (-0.02) | 5.94  (-0.03) | 2.67  (0.00) | 2.89  (0.00) | 5.01  (-0.02) | 4.42  (-0.03) | 4.18  (+0.03)  4.23  (+0.04) | 8.11  (+0.02) | 7.19  (-0.04) | 5.92  (-0.05) | 2.67  (0.00) | 2.87  (-0.02) | 5.01  (-0.02) | 4.40  (-0.05) | 4.18  (+0.03)  4.23  (+0.04) | | 8.12  (+0.03) | 7.18  (-0.05) | 5.88  (-0.09) | 2.66  (-0.01) | 2.84  (-0.05) | 5.01  (-0.02) | 4.35  (-0.10) | 4.18  (+0.03)  4.23  (+0.04) |
| **A6** | 8.09 | 7.60 | 6.13 | 2.57 | 2.90 | 4.99 | 4.45 | n.o.  4.25 | 8.10  (+0.01) | 7.59  (-0.01) | 6.12  (-0.01) | 2.57  (0.00) | 2.88  (-0.02) | 5.01  (+0.02) | 4.41  (-0.04) | n.o.  4.19  (-0.06) | 8.12  (+0.03) | 7.56  (-0.04) | 6.11  (-0.02) | 2.57  (0.00) | 2.87  (-0.03) | 5.01  (+0.02) | 4.40  (-0.05) | n.o.  4.18  (-0.07) | | 8.15  (+0.06) | 7.54  (-0.06) | 6.09  (-0.04) | 2.57  (0.00) | 2.84  (-0.06) | 5.01  (+0.02) | 4.36  (-0.09) | n.o.  4.16  (-0.09) |
| **T7** | 7.09 | 1.25 | 5.88 | 1.95 | 2.53 | 4.81 | 4.14 | 4.15 | 7.10  (+0.01) | 1.27  (+0.02) | 5.88  (0.00) | 1.96  (+0.01) | 2.53  (0.00) | 4.95  (+0.14) | 4.27  (+0.13) | n.o.  4.12  (-0.03) | 7.10  (+0.01) | 1.30  (+0.06) | 5.87  (-0.01) | 1.97  (+0.02) | 2.50  (-0.03) | 4.98  (+0.17) | 4.23  (+0.09) | n.o.  4.12  (-0.03) | | 7.13  (+0.04) | 1.35  (+0.10) | 5.85  (-0.03) | 1.97  (+0.02) | 2.44  (-0.09) | 4.98  (+0.17) | 4.24  (+0.10) | n.o.  4.09  (-0.06) |
| **T8** | 7.35 | 1.51 | 6.08 | 2.14 | 2.53 | 4.88 | 4.19 | 4.09 | 7.36  (+0.01) | 1.54  (+0.03) | 6.06  (-0.02) | 2.13  (-0.01) | 2.51  (-0.02) | 4.86  (-0.02) | 4.16  (-0.03) | n.o.  4.14  (+0.05) | 7.36  (+0.01) | 1.57  (+0.06) | 6.05  (-0.03) | 2.13  (-0.01) | 2.48  (-0.05) | 4.86  (-0.02) | 4.14  (-0.05) | n.o.  4.14  (+0.05) | | 7.39  (+0.04) | 1.60  (+0.09) | 6.02  (-0.06) | 2.13  (-0.01) | 2.45  (-0.08) | 4.86  (-0.02) | 4.12  (-0.07) | n.o.  4.15  (+0.06) |
| **C9** | 7.44 | 5.60 | 5.64 | 2.04 | 2.39 | 4.86 | 4.14 | 4.10  4.17 | 7.44  (0.00) | 5.67  (+0.07) | 5.66  (+0.02) | 1.99  (-0.05) | 2.36  (-0.03) | 4.86  (0.00) | 4.11  (-0.03) | 4.07  (-0.03)  4.15  (-0.02) | 7.44  (0.00) | 5.71  (+0.11) | 5.60  (-0.04) | 1.94  (-0.10) | 2.33  (-0.06) | 4.85  (-0.01) | 4.08  (-0.06) | 4.04  (-0.06)  4.14  (-0.03) | | 7.44  (0.00) | 5.76  (+0.16) | 5.61  (-0.03) | 1.90  (-0.14) | 2.29  (-0.10) | 4.84  (-0.02) | 4.07  (-0.07) | 4.02  (-0.08)  4.12  (-0.05) |
| **G10** | 7.89 | - | 5.83 | 2.54 | 2.67 | 4.97 | 4.41 | 4.04  4.15 | 7.93  (+0.04) | - | 5.80  (-0.03) | 2.59  (+0.05) | 2.69  (+0.02) | 4.99  (+0.02) | 4.33  (-0.08) | 4.02  (-0.02)  4.14  (-0.01) | 7.95  (+0.06) | - | 5.77  (-0.06) | 2.60  (+0.06) | 2.72  (+0.04) | 5.00  (+0.03) | 4.32  (-0.09) | 4.02  (-0.02)  4.14  (-0.01) | | 7.98  (+0.09) | - | 5.75  (-0.08) | 2.62  (+0.08) | 2.74  (+0.06) | 5.00  (+0.03) | 4.30  (-0.11) | 4.02  (-0.02)  4.14  (-0.01) |
| **C11** | 7.31 | 5.42 | 5.72 | 1.85 | 2.31 | 4.78 | 4.18 | 4.09  n.o. | 7.35  (+0.04) | 5.51  (+0.09) | 5.67  (-0.05) | 1.90  (+0.05) | 2.26  (-0.05) | 4.86  (+0.08) | 4.19  (+0.01) | 4.09  (0.00)  n.ο. | 7.36  (+0.05) | 5.55  (+0.13) | 5.64  (-0.08) | 1.90  (+0.05) | 2.23  (-0.08) | 4.86  (+0.08) | 4.19  (+0.01) | 4.12  (+0.03)  n.ο. | | 7.39  (+0.08) | 5.61  (+0.19) | 5.60  (-0.12) | 1.90  (+0.05) | 2.18  (-0.13) | 4.86  (+0.08) | 4.19  (+0.01) | 4.12  (+0.03)  n.ο. |
| **G12** | 7.92 | - | 6.13 | 2.37 | 2.58 | 4.66 | 4.19 | n.o.  n.o. | 7.92  (0.00) | - | 6.02  (-0.11) | 2.30  (-0.07) | 2.60  (+0.02) | 4.67  (+0.01) | 4.09  (-0.10) | n.o.  n.o. | 7.91  (-0.01) | - | 5.93  (-0.20) | 2.25  (-0.12) | 2.60  (+0.02) | 4.67  (+0.01) | 4.07  (-0.12) | n.o.  n.o. | | 7.91  (-0.01) |  | 5.86  (-0.27) | 2.22  (-0.15) | 2.60  (+0.02) | 4.67  (+0.01) | 4.04  (-0.15) | n.o.  n.o. |

**Table S10**: ^1^H NMR chemical shifts of the exchangeable imino and amino protons of the free d(5′-CGCGAATTGGCC-3′)_2_ (H_2_O : D_2_O, 9 : 1, 298 K, buffer phosphates 100 mM, pH = 7.0), and induced shifts upon the addition (**4a**) at r = 0.5, 1, and 2. Negative sign for upfield shifts and positive sign for downfield shifts (in parenthesis). In bold indicated shifts which are higher than 0.05 ppm. n.o. = not observed.

|  | *r = 0* | | | *r = 0.5* | | | *r = 1* | | | *r = 2* | | |
| --- | --- | --- | --- | --- | --- | --- | --- | --- | --- | --- | --- | --- |
|  | N1H | N2H | N2H* | N1H | N2H | N2H* | N1H | N2H | N2H* | N1H | N2H | N2H* |
| C1G12 | - | n.o. | n.o. | - | n.o. | n.o. | - | n.o. | n.o. | - | n.o. | n.o. |
| G2C11 | 13.04 | 8.44 | 6.57 | 12.68  (-0.36) | 8.43  (-0.01) | 6.68  (+0.11) | 12.51  (-0.53) | 8.41  (-0.03) | 6.75  (+0.18) | 12.35  (-0.69) | 8.40  (-0.04) | 6.81  (+0.24) |
| C3G10 | 12.88 | 8.36 | 6.41 | 12.63  (-0.25) | 8.37  (+0.01) | 6.63  (+0.22) | 12.41  (-0.47) | 8.39  (+0.03) | 6.79  (+0.38) | 12.38  (-0.50) | 8.40  (+0.04) | 6.91  (+0.50) |
| G4C9 | 12.68 | 8.40 | 6.79 | 12.52  (-0.16) | 8.52  (+0.12) | 7.07  (+0.28) | 12.42  (-0.26) | 8.56  (+0.16) | 7.23  (+0.44) | 12.37  (-0.31) | 8.60  (+0.20) | 7.35  (+0.56) |
| A5T8 | 13.75 | - | - | n.o. | - | - | n.o. | - | - | n.o. | - | - |
| A6T7 | 13.62 | - | - | 13.35  (-0.27) |  |  | 13.18  (-0.44) |  |  | 13.06  (-0.56) | - | - |

**Table S11**: ^1^H NMR chemical shifts of the exchangeable imino and amino protons of the free d(5′-CGCGAATTGGCC-3′)_2_ (H_2_O : D_2_O, 9 : 1, 298 K, buffer phosphates 100 mM, pH = 7.0), and induced shifts upon the addition (**5a**) at r = 0.5, 1, and 2. Negative sign for upfield shifts and positive sign for downfield shifts (in parenthesis). In bold indicated shifts which are higher than 0.05 ppm. n.o. = not observed.

|  | *r* = 0 | | | *r* = 0.5 | | | *r* = 1 | | | *r* = 2 | | |
| --- | --- | --- | --- | --- | --- | --- | --- | --- | --- | --- | --- | --- |
|  | N1H | N2H | N2H* | N1H | N2H | N2H* | N1H | N2H | N2H* | N1H | N2H | N2H* |
| C1G12 |  | n.o. | n.o. |  |  |  |  |  |  |  |  | n.o. |
| G2C11 | 13.04 | 8.44 | 6.57 | 13.02  (-0.02) | 8.44  (0.00) | 6.61  (+0.04) | 12.99  (-0.05) | 8.46  (+0.02) | 6.63  (+0.06) | 12.96  (-0.08) | 8.47  (+0.03) | 6.64  (+0.07) |
| C3G10 | 12.88 | 8.36 | 6.41 | 12.84  (-0.04) | 8.39  (+0.03) | 6.48  (+0.07) | 12.81  (-0.07) | 8.39  (+0.03) | 6.50  (+0.09) | 12.78  (-0.10) | 8.39  (+0.03) | 6.52  (+0.11) |
| G4C9 | 12.68 | 8.40 | 6.79 | 12.67  (-0.01) | 8.43  (+0.03) | 6.85  (+0.06) | 12.65  (-0.03) | 8.44  (+0.04) | 6.86  (+0.07) | 12.64  (-0.04) | 8.45  (+0.05) | 6.90  (+0.11) |
| A5T8 | 13.75 | - | - | 13.75  (0.00) | - | - | 13.73  (-0.02) | - | - | 13.72  (-0.03) | - | - |
| A6T7 | 13.62 | - | - | 13.59  (-0.03) | - | - | 13.56  (-0.06) | - | - | 13.53  (-0.09) | - | - |

**Table S12**: ^1^H NMR chemical shifts of the exchangeable imino and amino protons of the free d(5′-CGCGAATTGGCC-3′)_2_ (H_2_O : D_2_O, 9 : 1, 298 K, buffer phosphates 100 mM, pH = 7.0), and induced shifts upon the addition (**6a**) at r = 0.5, 1, and 2. Negative sign for upfield shifts and positive sign for downfield shifts (in parenthesis). In bold indicated shifts which are higher than 0.05 ppm. n.o. = not observed.

|  | *r = 0* |  |  | *r = 0.5* | | | *r = 1* | | | *r = 2* | | |
| --- | --- | --- | --- | --- | --- | --- | --- | --- | --- | --- | --- | --- |
|  | N1H | N2H | N2H* | N1H | N2H | N2H* | N1H | N2H | N2H* | N1H | N2H | N2H* |
| C1G12 |  | n.o. | n.o. |  | n.o. | n.o. |  | n.o. | n.o. |  | n.o. | n.o. |
| G2C11 | 13.04 | 8.44 | 6.57 | 12.91 (-0.13) | 8.46  (+0.02) | 6.65  (+0.08) | 12.82 (-0.22) | 8.47  (+0.03) | 6.70  (+0.13) | 12.64 (-0.40) | 8.48  (+0.04) | 6.74  (+0.17) |
| C3G10 | 12.88 | 8.36 | 6.41 | 12.80  (-0.08) | 8.42  (+0.06) | 6.52  (+0.11) | 12.74  (-0.14) | 8.46  (+0.10) | 6.59  (+0.18) | 12.69  (-0.19) | 8.48  (+0.12) | 6.67  (+0.26) |
| G4C9 | 12.68 | 8.40 | 6.79 | 12.66  (-0.02) | 8.45  (+0.05) | 6.87  (+0.08) | 12.64  (-0.04) | 8.48  (+0.08) | 6.92  (+0.13) | 12.64  (-0.04) | 8.52  (+0.12) | 6.99  (+0.20) |
| A5T8 | 13.75 | **-** | **-** | 13.73  (-0.02) | **-** | **-** | 13.72  (-0.03) | **-** | **-** | 13.69  (-0.06) | **-** | **-** |
| A6T7 | 13.62 | **-** | **-** | 13.58  (-0.04) | **-** | **-** | 13.54  (-0.08) | **-** | **-** | 13.47  (-0.15) | **-** | **-** |

**
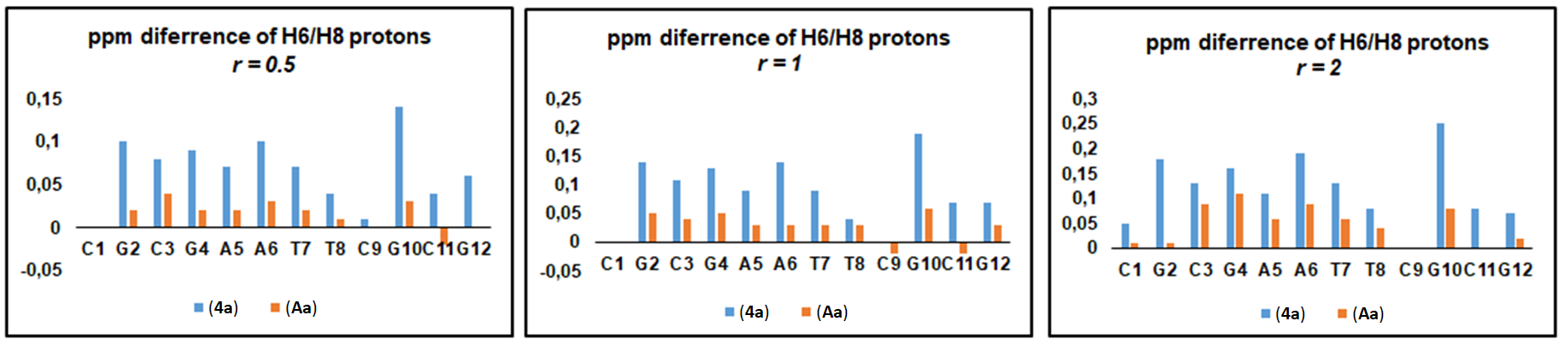
**

**Figure S60.** Comparison of chemical shift differences (Δδ, ppm) for the H6/H8 protons of the d(5′-CGCGAATTCGCG-3′)₂ duplex upon titration with complexes (**4a**) (blue) and the chloride salt of (**A**), (**Aa**), (orange) at three different ratios: (left) r = 0.5, (middle) r = 1, and (right) r = 2.


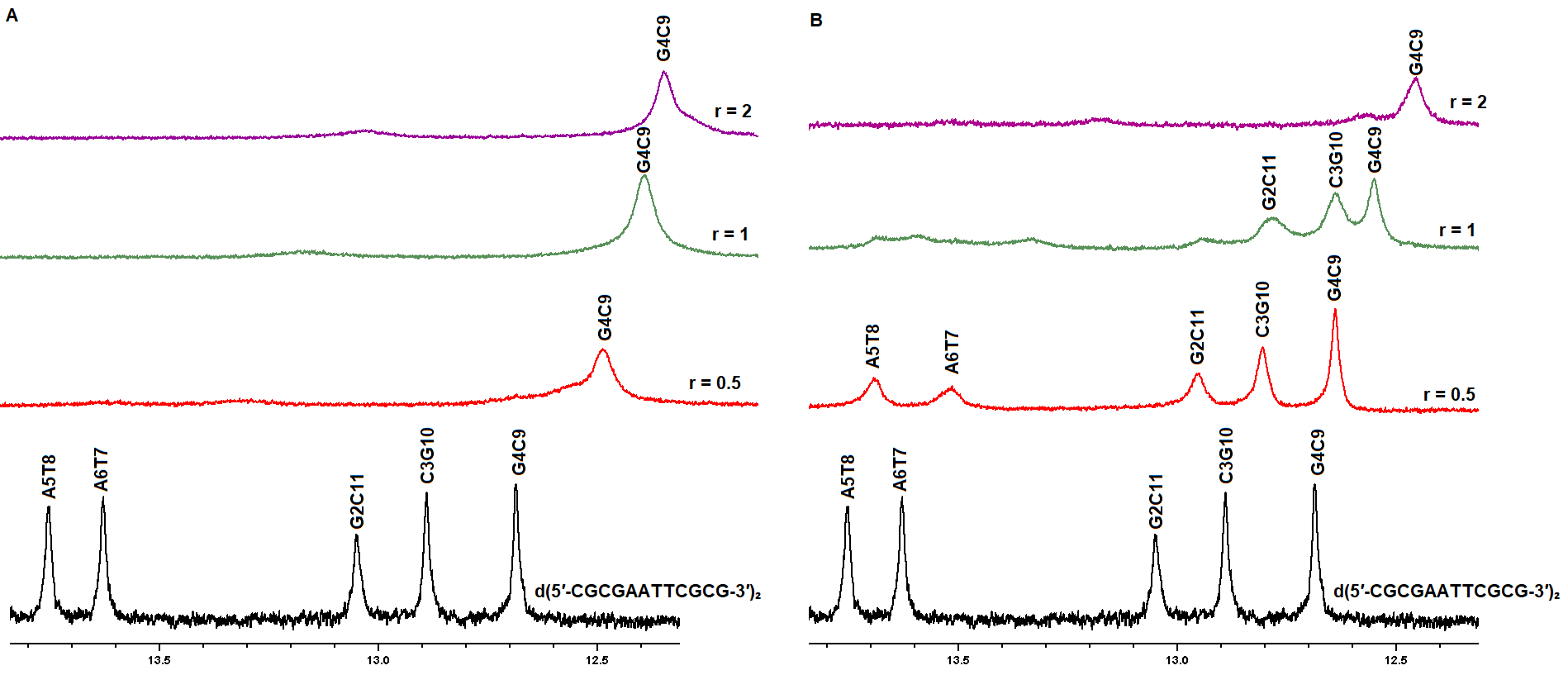


**Figure S61.** Imino protons regions of the ¹H NMR spectra (H₂O : D₂O, 9 : 1, 298 K, phosphate buffer 100 mM, pH = 7.0) with proton assignments, recorded during the titration of d(5′-CGCGAATTGGCC-3′)₂ with: (A) complex (**4a**) and (B) the chloride salt of (**B**), (**Ba**).


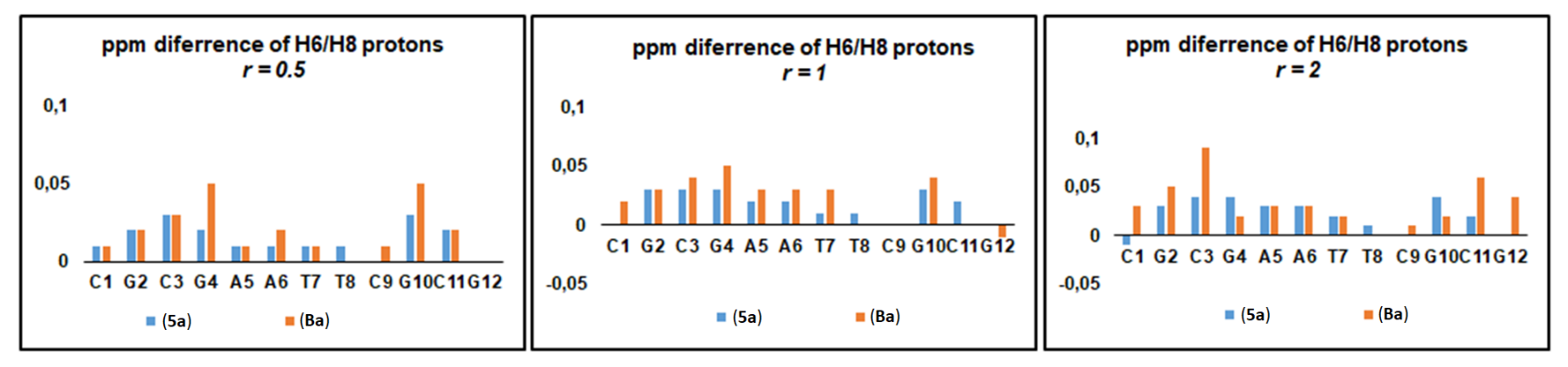
**Figure S62.** Comparison of chemical shift differences (Δδ, ppm) for the H6/H8 protons of the d(5′-CGCGAATTCGCG-3′)₂ duplex upon titration with complexes (**5a**) (blue) and the chloride salt of (**B**), (**Ba**), (orange) at three different ratios: (left) r = 0.5, (middle) r = 1, and (right) r = 2.

**
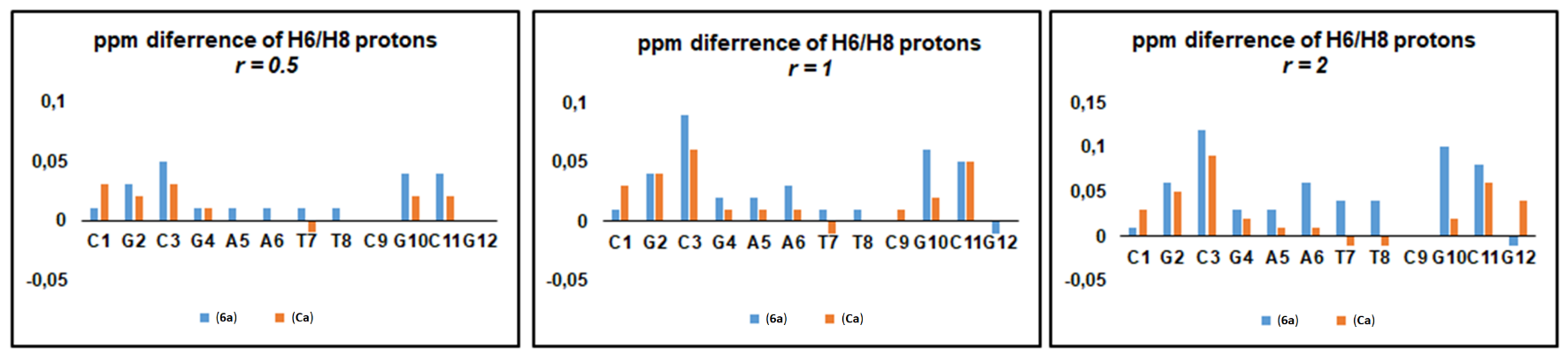
**

**Figure S63.** Comparison of chemical shift differences (Δδ, ppm) for the H6/H8 protons of the d(5′-CGCGAATTCGCG-3′)₂ duplex upon titration with complexes (**6a**) (blue) and the chloride salt of (**C**), (**Ca**) (orange) at three different ratios: (left) r = 0.5, (middle) r = 1, and (right) r = 2.


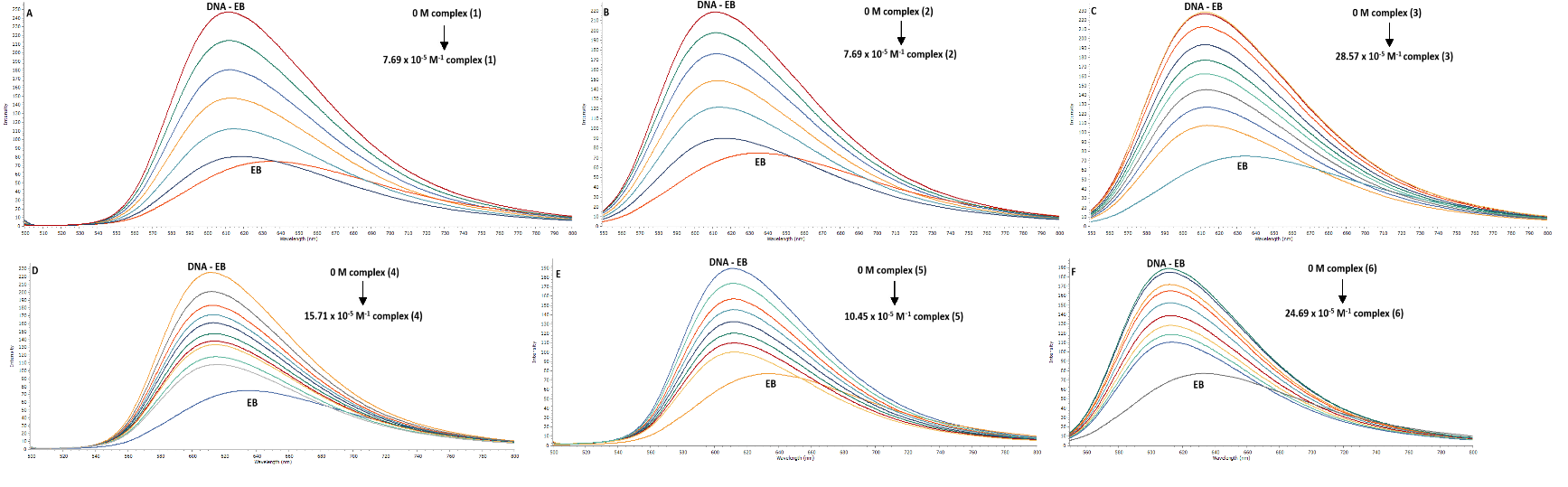


**Figure S64.** Fluorescence emission spectra of DNA–EtBr titrated with (**1a**)–(**6a**) at 298 K. [DNA] = 20 μM, [EB] = 5.2 μM, and [complex] = 0 to 30.10 μM. (A) (**1a**), (B) (**2a**), (C) (**3a**), (D) (**4a**), (E) (**5a**) and (F) (**6a**).


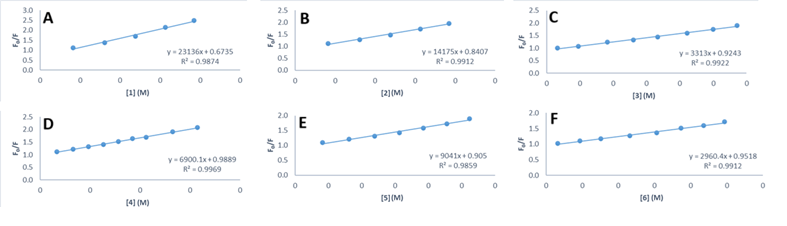


**Figure S65.** Stern–Volmer plots for the interaction of complexes with DNA–EB at 298 K. (A) (**1a**), (B) (**2a**), (C) (**3a**), (D) (**4a**), (E) (**5a**) and (F) (**6a**).


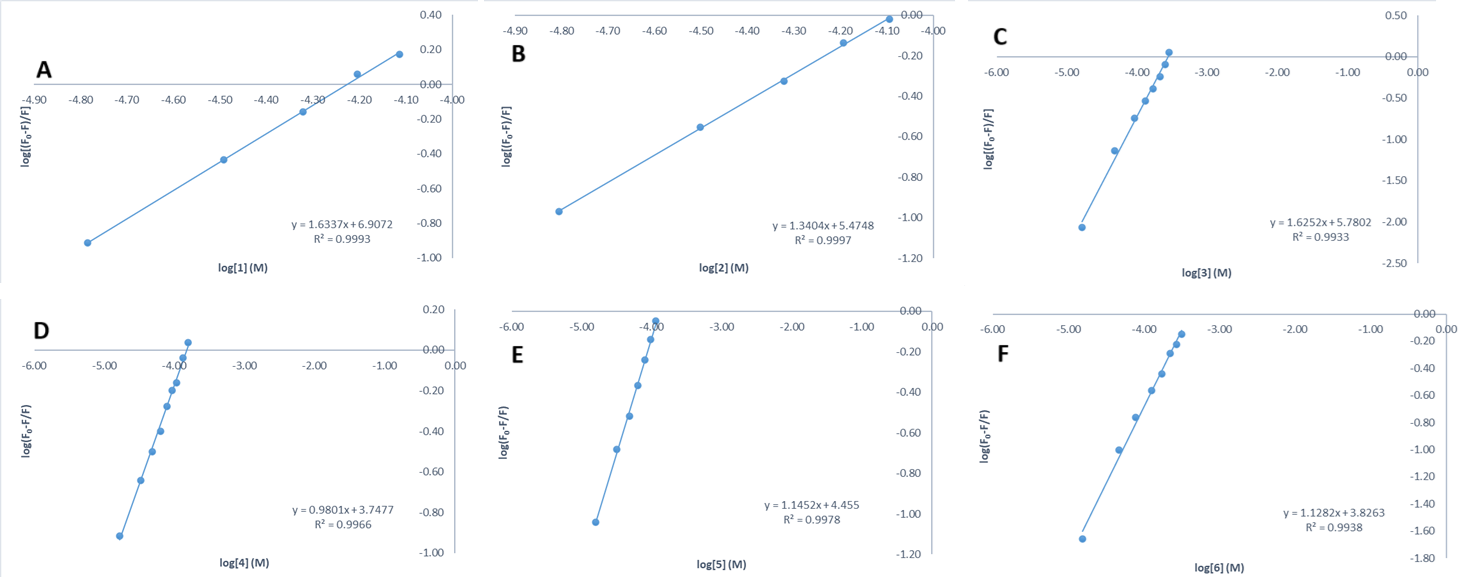


**Figure S66.** The double-log plot of complexes quenching effect on d(5′-CGCGAATTGGCC-3′)_2_-EB system fluorescence at 298 K. (A) (**1a**), (B) (**2a**), (C) (**3a**), (D) (**4a**), (E) (**5a**) and (F) (**6a**).
